# Supplementary material for: Optimizing differential expression analysis for proteomics data via high-performing rules and ensemble inference
Source: Nat Commun. 2024 May 9;15:3922. doi: 10.1038/s41467-024-47899-w (PMC11082229; doi:10.1038/s41467-024-47899-w)
Supplement: Supplementary file 1 — Supplementary Information [file 41467_2024_47899_MOESM1_ESM.pdf]

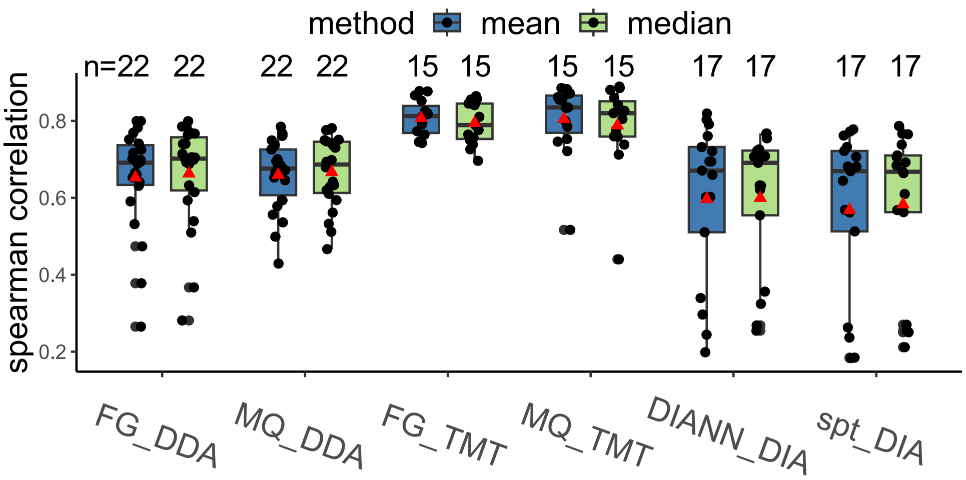

Supp. Fig. 1. The spearman correlations of the leave-one-dataset-out cross-validations using mean or median performance for workflow benchmarking. In the boxplots, the mean performances are marked by red triangles, centerline indicates the median, box limits indicate upper and lower quartiles, whiskers indicate the 1.5 interquartile range. The numbers of points for each boxplot (n) are shown above the boxplots.

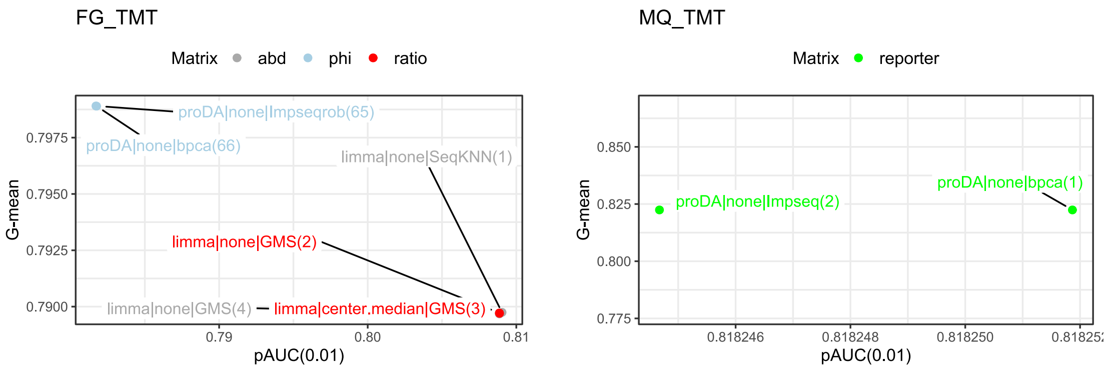

(a) (b)

Supp. Fig. 2 Top 2 workflows for each matrix type of settings FG_TMT and MQ_TMT. (a) shows the visualization of top-ranked workflows of setting FG_TMT for three types of available expression matrix types. The labels of the points show differential expression analysis tool names, normalization methods, imputation algorithms and rank positions (in the brackets) of the workflows. (b) shows the visualization of top-ranked workflows of setting MQ_TMT. Only reporter intensity is available.

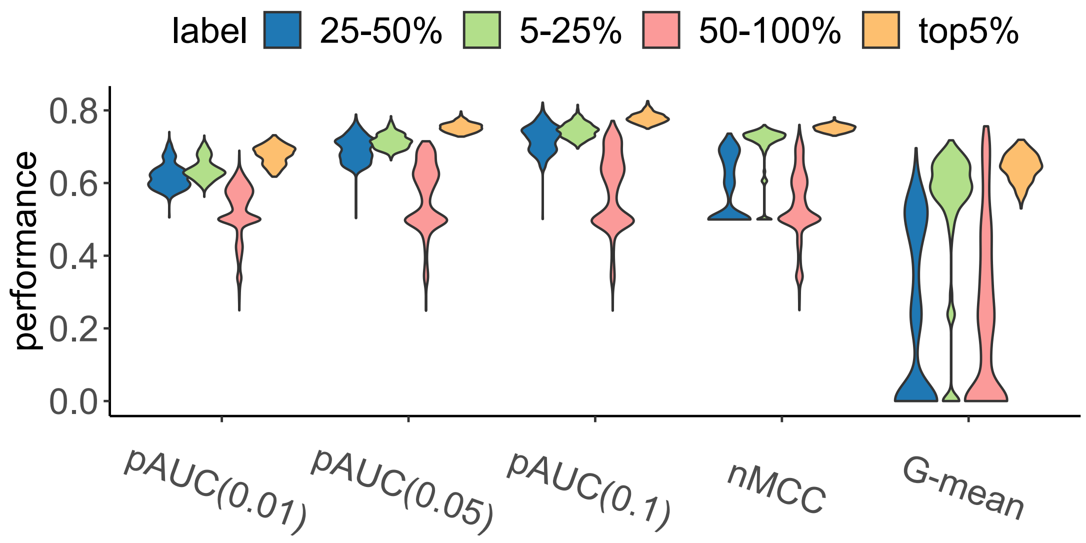

Supp. Fig. 3 Comparison of performance metric values of workflows ranked at top 5%, 5%-25%, 25%-50% and bottom 50% under setting FG_DDA.

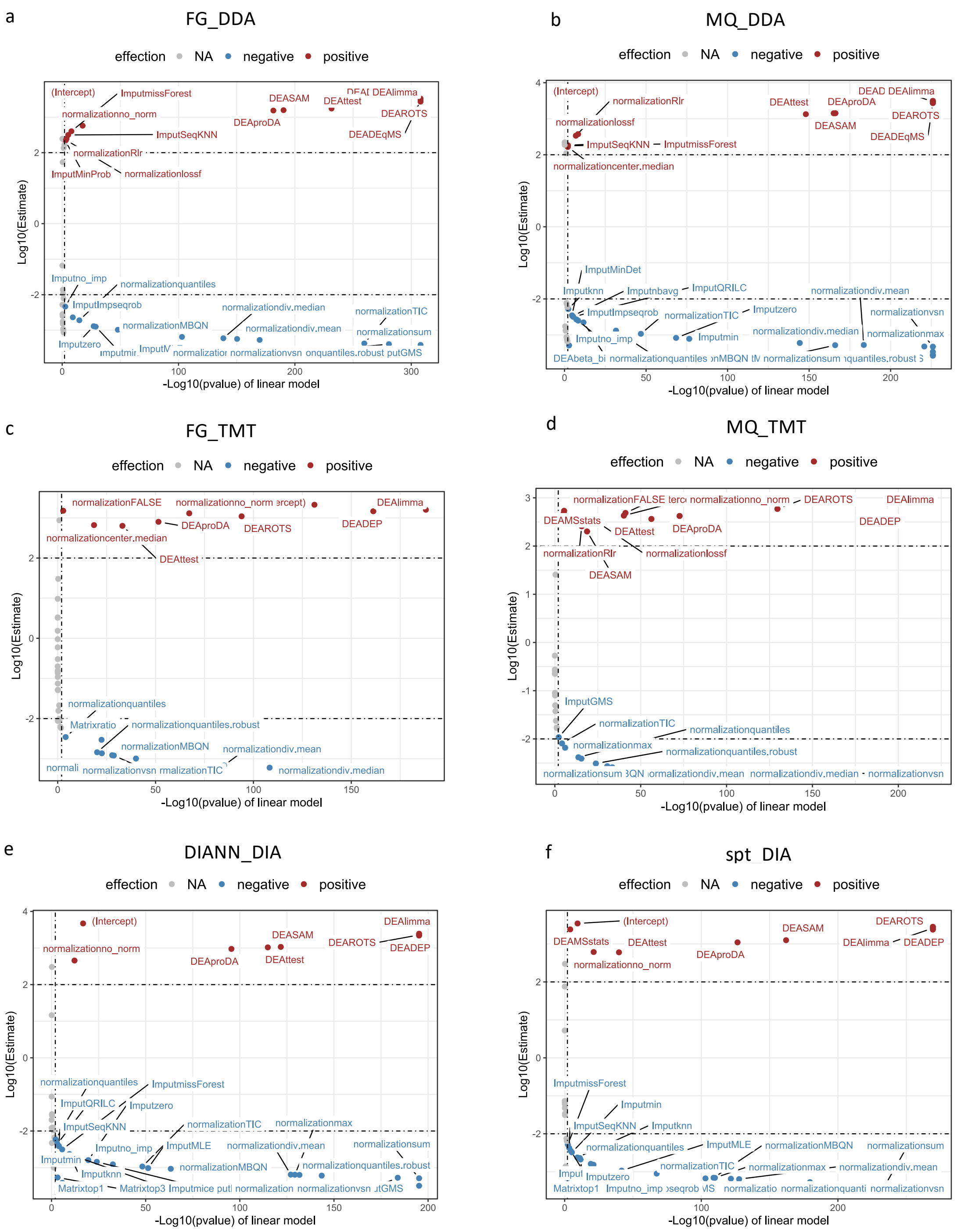

Supp. Fig. 4 Linear model fitting for checking affections of options in each workflow on the workflow ranking score. A-F visualize the linear model fitting results by plot the -log10(pvalue) against the log10(Estimate) with the data from settings FG_DDA (a), MQ_DDA (b), FG_TMT (c), MQ_TMT (d), DIANN_DIAN (e) and spt_DIA (f) respectively. Colors show the impact type where red means significant positive impact, blue means significant negative impact and gray means non-significant impact. The "Estimate" value provides the estimated coefficients for each predictor variable in the linear model. The p-value is calculated by a two-sided t-test.

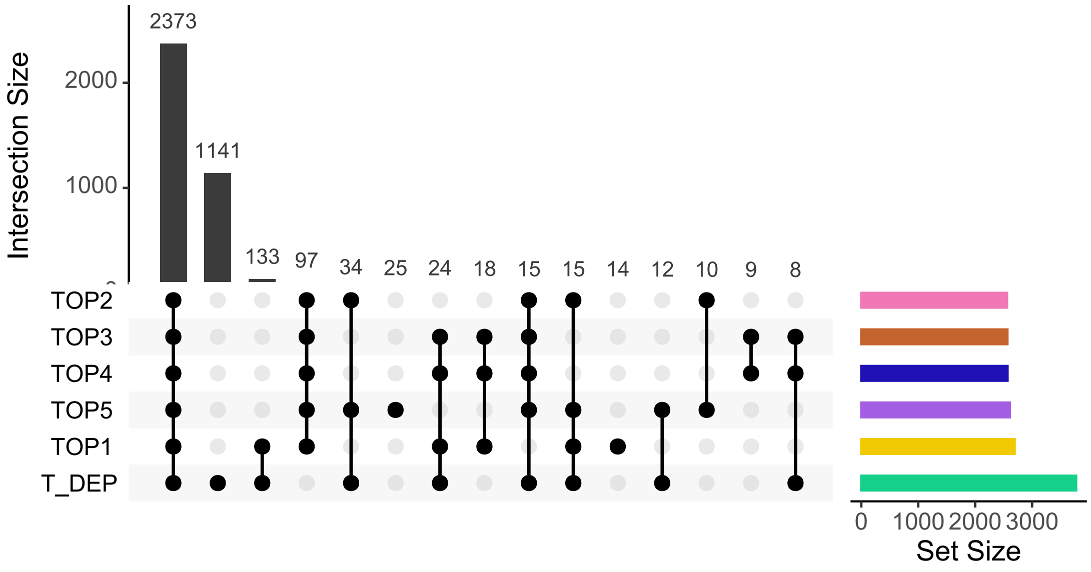

(a)

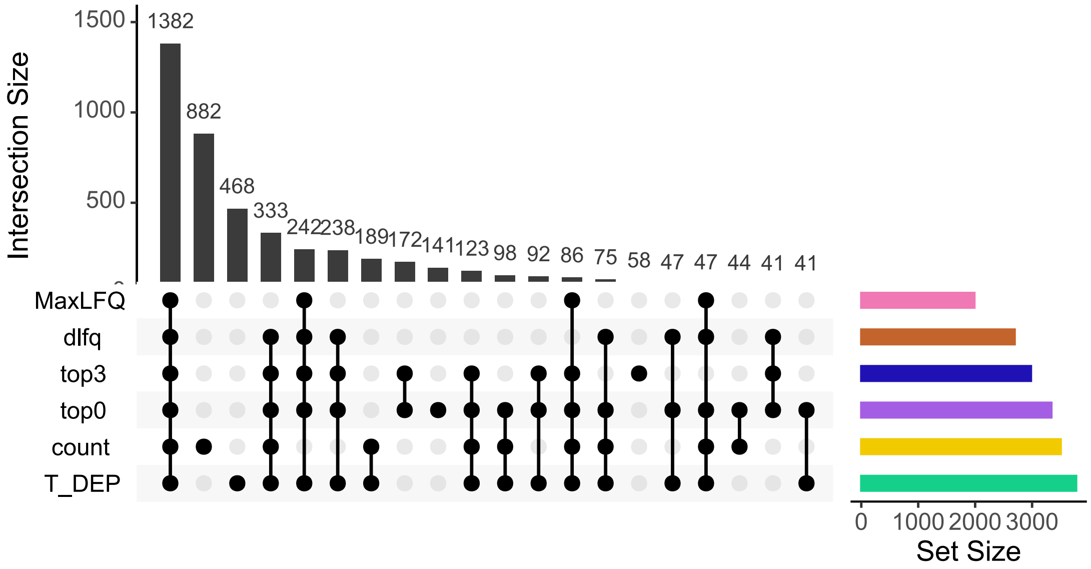

(b)

Supp. Fig. 5 comparison of DEPs detected by top ranked workflows. (a) compares the DEPs detected by 5 top-ranked workflows (top 5 of global ranking). (b) compares the DEPs detected by 5 best workflows that using the 5 available expression matrices as inputs (5 local best workflows). In the upset plots, only the top 20 intersections are shown in each plot.

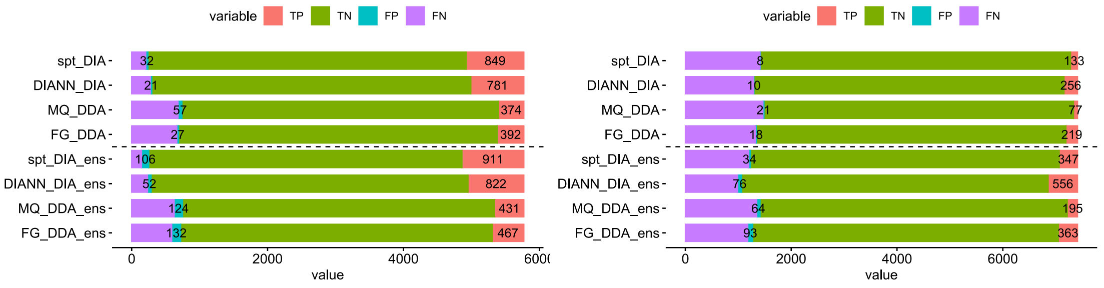

(a) (b)

Supp. Fig. 6 Cross-setting comparison with dataset pairs (HEqe408_LFQ, HEqe408_DIA) and (HYtims134_LFQ, HYtims134_DIA). (a) compares the best single workflows of DDA settings and DIA settings and their corresponding best ens_multi-quant settings with the dataset pair HEqe408_LFQ, HEqe408_DIA [1]. (b) compares the same workflows shown in (a) with dataset pair (HYtims134_LFQ, HYtims134_DIA [2]).

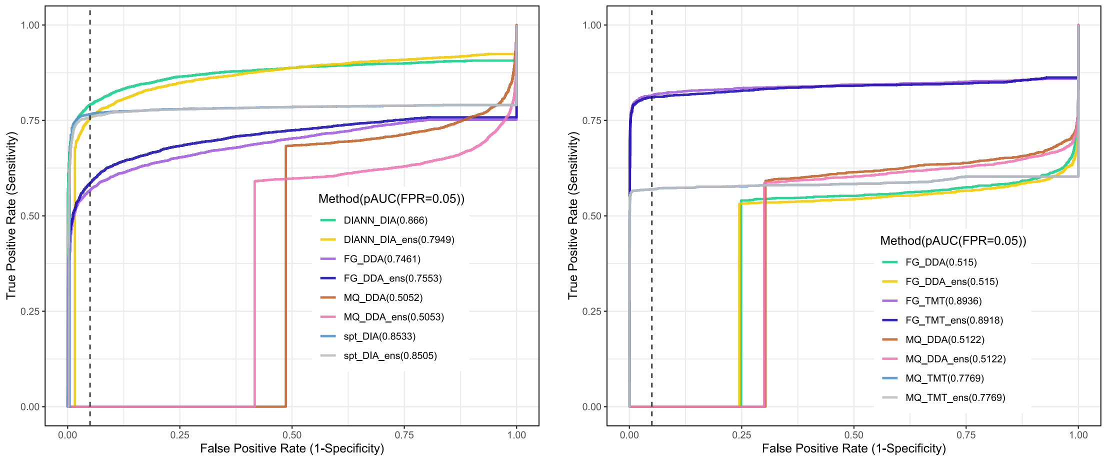

(a) (b)

Supp. Fig. 7 The Receiver Operating Characteristic (ROC) curves showing the performances of the best single workflows and best ens_multi-quant (for MQ_TMT, the ens_topk was tested instead) workflows under different settings. (a) shows the ROC curves of settings FG_DDA, MQ_DDA, DIANN_DIA and spt_DIA. The partial area under ROC curves with FPR<=0.05 (pAUC(0.05)s) are shown in the legend of the figure. (b) shows the ROC curves of settings FG_DDA, MQ_DDA, FG_TMT and MQ_TMT. The pAUC(0.05) scores can be found from the legend.

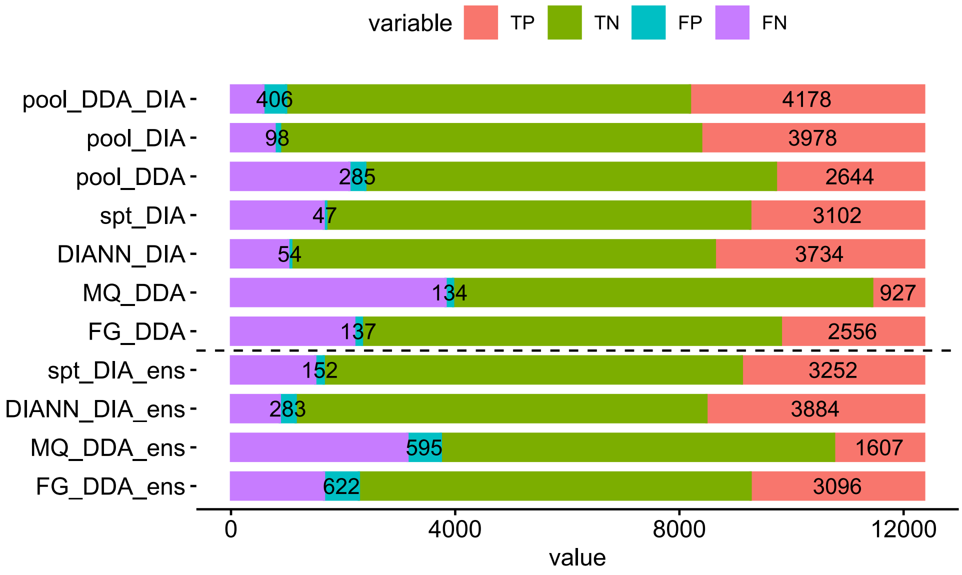

Supp. Fig. 8 Evaluation of the method of pooling detected DEPs from different quantification platforms. The method pool_DDA pools the DEPs detected by FG_DDA (the best workflow under setting FG_DDA) and MQ_DDA (the best workflow under setting MQ_DDA). The method pool_DIA pools the DEPs detected by DIANN_DIA and spt_DIA. Similarly, the method pool_DDA_DIA pools the DEPs detected by methods FG_DDA, MQ_DDA, DIANN_DIA and spt_DIA.

Supp. Tab. 1 The top-ranked workflows shown in Figure 2e and supp. Fig. 2

DEA	Matrix	Imput	norm	mean_ pauc001	mean_ pauc005	mean_ pauc01	mean_ nMCC	mean_ G-mean	setting	label
DEqMS	dlfq	missForest	none	0.7133	0.7794	0.8062	0.7653	0.6940	FG_DDA	DEqMS|none|missForest(1)
DEqMS	dlfq	SeqKNN	none	0.7159	0.7808	0.7986	0.7641	0.6787	FG_DDA	DEqMS|none|SeqKNN(2)
DEqMS	LFQ	Impseq	none	0.7225	0.7738	0.7905	0.7566	0.6489	FG_DDA	DEqMS|none|Impseq(18)
DEqMS	LFQ	SeqKNN	none	0.7315	0.7788	0.7896	0.7644	0.6366	FG_DDA	DEqMS|none|SeqKNN(20)
DEqMS	top0	missForest	none	0.6601	0.7440	0.7812	0.7558	0.7146	FG_DDA	DEqMS|none|missForest(103)
ROTS	top0	nbavg	none	0.6354	0.7580	0.7958	0.7569	0.7003	FG_DDA	ROTS|none|nbavg(129)
DEqMS	top3	SeqKNN	none	0.6551	0.7433	0.7767	0.7479	0.6686	FG_DDA	DEqMS|none|SeqKNN(200)
DEqMS	top3	nbavg	none	0.6511	0.7368	0.7729	0.7534	0.6817	FG_DDA	DEqMS|none|nbavg(222)
plgem	count	none	none	0.6284	0.6830	0.7081	0.6850	0.5467	FG_DDA	plgem|none|none(1926)
plgem	count	QRILC	none	0.6284	0.6830	0.7081	0.6850	0.5467	FG_DDA	plgem|none|QRILC(1929)
DEqMS	dlfq	Impseq	none	0.7188	0.7937	0.8173	0.7676	0.6826	MQ_DDA	DEqMS|none|Impseq(1)
limma	dlfq	Impseq	none	0.7147	0.7935	0.8173	0.7679	0.6809	MQ_DDA	limma|none|Impseq(2)
DEqMS	LFQ	SeqKNN	lossf	0.7276	0.7681	0.7795	0.7783	0.6483	MQ_DDA	DEqMS|lossf|SeqKNN(18)
limma	LFQ	SeqKNN	lossf	0.7259	0.7681	0.7800	0.7774	0.6448	MQ_DDA	limma|lossf|SeqKNN(22)
limma	top3	Impseq	Rlr	0.6751	0.7537	0.7816	0.7419	0.6407	MQ_DDA	limma|Rlr|Impseq(97)
ROTS	top3	SeqKNN	Rlr	0.6496	0.7640	0.7970	0.7585	0.6636	MQ_DDA	ROTS|Rlr|SeqKNN(99)
ROTS	top0	SeqKNN	Rlr	0.6493	0.7479	0.7765	0.7435	0.6227	MQ_DDA	ROTS|Rlr|SeqKNN(233)
ROTS	top0	missForest	Rlr	0.6440	0.7450	0.7748	0.7433	0.6306	MQ_DDA	ROTS|Rlr|missForest(259)
plgem	count	none	none	0.6044	0.6499	0.6721	0.6497	0.4125	MQ_DDA	plgem|none|none(3341)
plgem	count	QRILC	none	0.6044	0.6499	0.6721	0.6497	0.4125	MQ_DDA	plgem|none|QRILC(3344)
limma	dlfq	MinDet	none	0.7671	0.8212	0.8416	0.7898	0.6511	DIANN_DIA	limma|none|MinDet(1)
limma	dlfq	Impseqrob	none	0.7615	0.8215	0.8437	0.7886	0.6471	DIANN_DIA	limma|none|Impseqrob(2)
limma	LFQ	Impseq	none	0.7680	0.8236	0.8458	0.7893	0.6340	DIANN_DIA	limma|none|Impseq(3)
ROTS	LFQ	Impseq	none	0.7351	0.8285	0.8532	0.7906	0.6471	DIANN_DIA	ROTS|none|Impseq(7)
limma	top3	Impseq	none	0.6896	0.7542	0.7798	0.7414	0.6214	DIANN_DIA	limma|none|Impseq(316)
ROTS	top3	Impseq	none	0.6543	0.7529	0.7877	0.7375	0.6211	DIANN_DIA	ROTS|none|Impseq(380)
limma	top1	Impseq	none	0.6321	0.7027	0.7309	0.7013	0.5925	DIANN_DIA	limma|none|Impseq(788)
limma	top1	bpca	none	0.6265	0.6970	0.7241	0.6939	0.5810	DIANN_DIA	limma|none|bpca(883)
ROTS	dlfq	MinDet	none	0.7426	0.8072	0.8299	0.7856	0.6524	spt_DIA	ROTS|none|MinDet(1)
ROTS	dlfq	Impseq	none	0.7456	0.8113	0.8352	0.7823	0.6453	spt_DIA	ROTS|none|Impseq(2)
ROTS	LFQ	Impseq	none	0.7207	0.8221	0.8506	0.7845	0.6593	spt_DIA	ROTS|none|Impseq(9)
ROTS	LFQ	bpca	none	0.7205	0.8201	0.8487	0.7833	0.6587	spt_DIA	ROTS|none|bpca(10)
ROTS	top3	nbavg	none	0.6927	0.7856	0.8139	0.7793	0.6489	spt_DIA	ROTS|none|nbavg(68)
ROTS	top3	MinDet	none	0.6940	0.7831	0.8112	0.7651	0.6255	spt_DIA	ROTS|none|MinDet(90)
limma	top1	Impseq	none	0.6639	0.7407	0.7682	0.7315	0.6278	spt_DIA	limma|none|Impseq(411)
DEP	top1	Impseq	none	0.6554	0.7393	0.7683	0.7123	0.5847	spt_DIA	DEP|none|Impseq(585)

Supp. Tab. 2 The top-ranked workflows shown in supp. Fig. 2

DEA	Matrix	Imput	norm	mean_ pauc001	mean_ pauc005	mean_ pauc01	mean_ nMCC	mean_ G-mean	setting	label
**proDA**	reporter	bpca	none	0.8183	0.9211	0.9396	0.8865	0.8224	MQ_TMT	proDA|none|bpca(1)
**proDA**	reporter	Impseq	none	0.8182	0.9211	0.9396	0.8865	0.8224	MQ_TMT	proDA|none|Impseq(2)
**limma**	abd	SeqKNN	none	0.8090	0.9073	0.9291	0.8753	0.7897	FG_TMT	limma|none|SeqKNN(1)
**limma**	abd	GMS	none	0.8089	0.9073	0.9290	0.8753	0.7897	FG_TMT	limma|none|GMS(4)
**limma**	ratio	GMS	none	0.8089	0.9073	0.9290	0.8753	0.7897	FG_TMT	limma|none|GMS(2)
**limma**	ratio	GMS	center.median	0.8089	0.9073	0.9290	0.8753	0.7897	FG_TMT	limma|center.median|GMS(3)
**proDA**	phi	Impseqrob	none	0.7817	0.8910	0.9168	0.8682	0.7989	FG_TMT	proDA|none|Impseqrob(65)
**proDA**	phi	bpca	none	0.7818	0.8910	0.9168	0.8681	0.7989	FG_TMT	proDA|none|bpca(66)

Supp. Tab. 3 ANOVA F-value and p-values extracted from the linear model and the feature importance obtained from the workflow performance level classification.

		**DEA**	**Imputation**	**normalization**	**Matrix**
**FG_DDA**	F value	776.52	371.97	695.85	131.02
	Pr(>F)	0	0	0	1.78E-108
	importance	29.02	21.63	33.71	15.65
**MQ_DDA**	F value	912.11	415.15	703.03	87.96
	Pr(>F)	0	0	0	3.14E-73
	importance	29.50	23.39	33.16	13.95
**FG_TMT**	F value	261.42	1.03	217.04	23.31
	Pr(>F)	0	0.42	0	3.36E-23
	importance	28.46	9.49	38.31	23.74
**MQ_TMT**	F value	321.83	1.27	165.13	NA
	Pr(>F)	2.11E-296	0.21	1.47E-294	NA
	importance	40.99	13.8	45.22	NA
**DIANN_DIA**	F value	707.44	238.53	311.67	887.51
	Pr(>F)	0	0	0	0
	importance	25.68	20.35	28.62	25.36
**spt_DIA**	F value	1012.35	63.28	388.16	690.18
	Pr(>F)	0	7.5E-147	0	0
	importance	30.84	15.97	31.47	21.73

Supp. Tab. 4 The compared top-ranked single workflows and ensemble inference workflows

setting	workflow	ensembele type	p-value integration method
**FG_DDA**	DEqMS|FragPipe|dlfq|missForest|blank	-	-
**MQ_DDA**	DEqMS|Maxquant|dlfq|Impseq|blank	-	-
**FG_TMT**	limma|FragPipe|abd|SeqKNN|blank	-	-
**MQ_TMT**	proDA|Maxquant|intensity|bpca|blank	-	-
**DIANN_DIA**	limma|DIANN|dlfq|MinDet|blank	-	-
**spt_DIA**	ROTS|spt|dlfq|MinDet|blank	-	-
**FG_DDA**	ensemble_hurdle||LFQ|dlfq|top0	ens_multi-quant	hurdle
**MQ_DDA**	ensemble_hurdle||LFQ|dlfq|top0	ens_multi-quant	hurdle
**FG_TMT**	ensemble_fisher||abd|phi	ens_multi-quant	fisher
**DIANN_DIA**	ensemble_hurdle||LFQ|dlfq|top3	ens_multi-quant	hurdle
**spt_DIA**	ensemble_hurdle||LFQ|dlfq|top3	ens_multi-quant	hurdle
**FG_DDA**	ensemble_set_median||top18	ens_topk	set/median; k=18
**MQ_DDA**	ensemble_set_min||top19	ens_topk	set/min; k=19
**FG_TMT**	ensemble_hurdle||top2	ens_topk	hurdle; k=2
**MQ_TMT**	ensemble_fisher||top2	ens_topk	fisher; k=2
**DIANN_DIA**	ensemble_set_min||top5	ens_topk	set/min; k=5
**spt_DIA**	ensemble_set_min||top12	ens_topk	set/min; k=12

Supp. Tab. 5 The performance gains obtained by ensemble inference workflows compared to single workflows. TOP1 refers to the best single workflow for each setting.

**setting**	**compare**	**performance gains**									
		Mean pAUC0.01	Median pAUC0.01	Mean pAUC0.05	Median pAUC0.05	Mean pAUC0.1	Median pAUC0.1	Mean nMCC	Median nMCC	Mean Gmean	Median_G-mean
FG_DDA	ens_multi_quant - TOP1	0.0350	0.0376	0.0305	0.0376	0.0256	0.0281	-0.0151	-0.0396	0.0702	0.0195
	ens_topk - TOP1	0.0046	0.0238	0.0066	0.0179	0.0035	0.0112	0.0018	0.0030	-0.0144	0.0176
MQ_DDA	ens_multi_quant - TOP1	0.0461	0.0615	0.0324	0.0398	0.0316	0.0575	0.0100	0.0132	0.0912	0.0560
	ens_topk - TOP1	0.0087	0.0270	0.0163	0.0443	0.0204	0.0510	0.0069	0.0095	0.0513	0.0275
FG_TMT	ens_multi_quant - TOP1	0.0117	0.0130	0.0095	0.0087	0.0093	0.0043	0.0174	0.0058	0.0579	0.0047
	ens_topk - TOP1	0.0004	-0.0001	0.0001	0.0000	0.0000	0.0000	0.0001	0.0000	0.0013	0.0000
MQ_TMT	ens_topk - TOP1	0.0012	-0.0018	0.0002	0.0014	0.0001	0.0007	0.0104	-0.0008	0.0266	0.0002
DIANN_DIA	ens_multi_quant - TOP1	0.0229	0.0217	0.0344	0.0409	0.0387	0.0352	0.0100	-0.0650	0.1114	0.1163
	ens_topk - TOP1	0.0280	0.0466	0.0361	0.0466	0.0379	0.0452	0.0258	0.0019	0.0903	0.0900
spt_DIA	ens_multi_quant - TOP1	0.0415	0.0450	0.0446	0.0447	0.0445	0.0411	0.0110	-0.0271	0.0969	0.1564
	ens_topk - TOP1	0.0293	0.0044	0.0345	0.0197	0.0342	0.0233	-0.0667	-0.0787	-0.1587	-0.2041

Supp. Tab. 6 Testing the pooling strategy-based cross-platform DEA integration

Method	pAUC(0.01)	pAUC(0.05)	pAUC(0.1)	TP	TN	FP	FN	Recall	Precision	Specificity	G-mean	nMCC
**FG_DDA**	0.6855	0.7461	0.7611	2556	7474	137	2199	0.5375	0.9491	0.9820	0.7265	0.8062
MQ_DDA	0.5010	0.5052	0.5107	927	7477	134	3828	0.1950	0.8737	0.9824	0.4376	0.6540
DIANN_DIA	0.8180	0.8660	0.8803	3734	7557	54	1021	0.7853	0.9857	0.9929	0.8830	0.9106
spt_DIA	0.7688	0.8533	0.8643	3102	7564	47	1653	0.6524	0.9851	0.9938	0.8052	0.8608
FG_DDA_ens	0.7050	0.7553	0.7723	3096	6989	622	1659	0.6511	0.8327	0.9183	0.7732	0.8020
MQ_DDA_ens	0.5010	0.5053	0.5110	1607	7016	595	3148	0.3380	0.7298	0.9218	0.5582	0.6652
DIANN_DIA_ens	0.6031	0.7949	0.8360	3884	7328	283	871	0.8168	0.9321	0.9628	0.8868	0.9012
spt_DIA_ens	0.7772	0.8505	0.8614	3252	7459	152	1503	0.6839	0.9553	0.9800	0.8187	0.8616
pool_DDA	0.6594	0.7283	0.7480	2644	7326	285	2111	0.5560	0.9027	0.9626	0.7316	0.7967
pool_DIA	0.7789	0.8785	0.8967	3978	7513	98	777	0.8366	0.9760	0.9871	0.9087	0.9262
pool_DDA_DIA	0.7772	0.8825	0.9031	4178	7205	406	577	0.8787	0.9114	0.9467	0.9120	0.9156

Supp. Tab. 7 Top-ranked workflow running time comparison

Setting	Method	Dataset	Workflows involved	Running time
DIANN_DIA	TOP1	HEqe408_DIA	1	27.94s
DIANN_DIA	ens_multi-quant	HEqe408_DIA	3	207.03s
spt_DIA	TOP1	HEqe408_DIA	1	103.4s
spt_DIA	ens_multi-quant	HEqe408_DIA	3	254.48s

Supplementary Note 1

Leave-One-Dataset-out cross-validation proves the good generalizability of our benchmarking

A workflow's performance is indicated by 5 different metrics such as pAUC(0.01), pAUC(0.05), pAUC(0.1) [3], nMCC [4] and G-mean [5] (see our Methods in main text). Multiple datasets were used to evaluate each workflow, e.g., 12 DDA datasets with 22 contrasts were used to evaluate 7852 candidate workflows. For a given metric type, e.g., pAUC(0.01), both the mean and median pAUC(0.01) values calculated from the 22 contrasts can be used to rank the 7852 workflows. For each workflow, five rank positions can be obtained from the five metrics. The final rank position of a workflow is calculated as the average value of the five rank positions (see Methods).

To measure the generalizability of our benchmarking results and to check whether our benchmarking could be used for guiding optimal workflow selection for newcoming data, we conducted the Leave-One-Dataset-Out cross-validation (LODOCV). In each round of the LODOCV, we leave one dataset as testing data and the remaining datasets are used for benchmarking (see Methods). We checked the LODOCV results obtained by using mean metric value or median metric value to rank workflows during the benchmarking. supp. Fig. 1 shows the performance distributions of the LODOCVs under 6 quantification settings. The detailed spearman correlation coefficients (R) can be found in Supplementary Data 1.

We can see, mean metric value based benchmarking and median metric value-based benchmarking obtain similar performances in the LODOCVs when using the average spearman correlation as the indicator. Under setting FG_DDA [6], the average spearman correlation coefficients (R) are 0.65 and 0.66 for mean and median based benchmarking respectively. Under setting MQ_DDA [7], averagely R=0.66 and R=0.67 are achieved by mean and median-based benchmarking. For settings FG_TMT [6,8] and MQ_TMT [7], the average correlation coefficients are as high 0.8 no matter using mean (R=0.81 and R=0.80 for FG_TMT and MQ_TMT) or median (R=0.79 and R=0.79) for benchmarking. For DIANN_DIA [9] workflows, average R=0.60 and R=0.60 are obtained by mean and median while for spt_DIA [10] R=0.57 and R=0.58 are achieved averagely by them.

Visualization of Top 2 workflows for each matrix type of settings FG_TMT and MQ_TMT

We plot the top 2 workflows with different expression matrices as inputs under settings of FG_TMT and MQ_TMT. Under setting FG_TMT, three types of expression matrices are available such as TMT-Integrator abundance (abd), TMT-Integrator ratio (ratio) and philosopher intensity (phi) [11]. Under setting MQ_TMT, only the reporter intensity was obtained for protein quantification. The top ranked workflows are visualized by plot their mean pAUC(0.01) values against their mean G-mean values. The differences between the performances of these top-ranked workflows are small (some points are nearly overlapped, see detailed values in our Supplementary Data 2).

In supp. Tab. 1, the top-ranked workflows shown in Fig. 2e and their metric values are listed. In supp. Tab. 2, the top-ranked workflows under settings FG_TMT and MQ_TMT (shown in supp.

Fig2) and their metric values are shown.

We can observe big performance gap between top-ranked workflows and bottom-ranked ones. In supp. Fig. 3, we plot the performance metric values of FG_DDA workflows ranked at top 5%, between 5% and 25%, between 25% and 50% and bottom 50% respectively. We can see, comparing the five performance metric values of the top 5% workflows (colored in orange) with the bottom 50% workflows (colored in red), there are big differences.

Fitting linear model to check the interactions between options in workflow steps and the workflow performance ranking

In each of the four workflow steps such as choosing expression matrix type (Matrix), matrix normalization (normalization), missing data imputation (Imputation) and differential expression analysis with a statistic tool (DEA tool), multiple options are available. To investigate the affection of option changes on workflow performance rankings, we fitted linear models by regarding the workflow ranking score (N – rank position, N means the total number of workflows, see Methods) as response variable and the four workflow steps as predictor variables (see Methods). The variables "Matrix", "normalization", "Imputation" and "DEA tool" are category variables with multiple levels (workflow step options). A categorical variable with n levels will be transformed into n-1 variables each with two levels via a dummy encoding. Then, these n-1 new variables contain the same information than the single variable (see the tutorial in http://www.sthda.com/english/articles/40-regression-analysis/163-regression-with-categorical-variables-dummy-coding-essentials-in-r/#google_vignette). We implemented the linear model fitting with the following R code (R-4.3.1):

model <- lm (ranking_score ~ DEA + Imput + normalization + Matrix, data = FG_DDA) (1)

The four categorical variables can be dummy encoded into four sets of predictor variables automatically. Then, the affection of each predictor variable (x), i.e., an option in a workflow step, on the response variable (y), i.e., workflow ranking score, can be measured with the "Estimate" value (also known as the coefficient) and p-value obtained from the fitted linear model. The "Estimate" value indicates the average increase in the response variable associated with a one unit increase in the predictor variable, assuming all other predictor variables are held constant (https://www.statology.org/interpret-regression-output-in-r/). Positive "Estimate" value means positive impact of the predictor variable otherwise negative impact is caused. The p-value indicates whether the predictor variable is statistically significant to the response variable (calculated by a two-sided t-test).

In supp. Fig. 4, we plot the -log10(p-value) against log10(Estimate) to visualize the affections of different options in the four workflow steps on the workflow performance rankings. We can see, DEA tools limma [12], ROTS [13], DEP [14] always show positive and higher impact to workflow rankings under 6 settings, in other words, the adoption of limma, ROTS and DEP can improve the ranking of a workflow comparing to other DEA tools (supp. Fig. 4a-f and Supplementary Data 4).

For DDA settings, the DEA tool DEqMS [15] also positively affect the workflow performance (supp. Fig. 4a-b and Supplementary Data 4). For normalization, “no_norm” shows positive impact across different settings except for MQ_DDA (supp. Fig. 4b and Supplementary Data 4). The two regression-based normalization methods lossf [16] and Rlr [17] work well under FG_DDA, MQ_DDA and MQ_TMT (supp. Fig. 4a-b, d and Supplementary Data 4). From supp. Fig. 4a (Supplementary Data 4), we also can find some imputation algorithms showing positive impacts such as missForest [18], SeqKNN [19] and MinProb[20] under settings of FG_DDA and MQ_DDA. More details about the interactions between single predictor variables and response variables can be found in Supplementary Data 4.

We also checked the interactions between predictor variables, e.g., normalization methods and imputation algorithms. The following example R code is used to analysis the interactions of normalization methods and imputation algorithms under setting FG_DDA:

model <- lm (ranking_score ~ Imput * normalization, data = FG_DDA)   (2)

However, we cannot find valuable outputs from the predictor interactions.

In addition, we extract ANOVA [21] tables from the linear model to check whether significant differences of mean ranking scores exist among workflow groups formed by options available in a workflow step. The F-value [22] which is defined as the ratio of the variation between sample means to the variation within the samples is used to indicate the affection of a category variable on the workflow ranking score and the p-value (two sided t-test) means whether the group difference is statistically significant (https://www.scribbr.com/statistics/anova-in-r/).

The F-values and p-values obtained from the ANOVA are shown in following supp. Tab. 3. We also list feature important obtained from the workflow performance level classification in supp. Tab. 3. We can see, under label-free settings (FG_DDA, MQ_DDA, DIANN_DIA and spt_DIA) the p-values of the ANOVA are <0.05 were obtained for all the four workflow steps, which means significant differences exist. In addition, we can observe good consistency between feature importance and feature F-values. For example, the features DEA and normalization always obtained higher feature importance and higher F-values comparing to Imputation and Matrix under settings FG_DDA, MQ_DDA, FG_TMT and MQ_TMT. Under settings DIANN_DIA and spt_DIA, Matrix, normalization and DEA obtained higher feature importance and F-values than Imputation.

Comparing the differentially expressed proteins detected by expression matrix-specific top 1st workflows and the 5 globally top-ranked workflows

We are interested in the consistency among differentially expressed proteins (DEPs) detected by the top-ranked workflows. We used the HYEtims735_LFQ [23] dataset as a case study. Differentially expressed proteins are defined as the proteins with logFC>=log2(1.5) and q-value<=0.05. We compared the differentially expressed proteins detected by the 5 top-ranked

workflows based on our benchmarking. Below supp. Fig. 5 (a) shows the intersections among the DEPs detected by the ranked-best (Top1), the ranked 2nd (Top2), ranked 3rd (Top3), ranked 4th (Top4) and ranked 5th (Top5) workflows and the true DEPs (T_DEP) detectable by the 5 workflows (union the true DEPs from the proteins available in the DEA results of the 5 workflows) under setting FG_DDA. We refer these 5 top-ranked workflows as 5 globally top-ranked workflows. supp. Fig. 5 (b) shows the DEPs detected by the dlfq-based best workflow (dlfq [24], the ranked best workflow among the workflows using dlfq intensity as expression matrix), the MaxLFQ [25]-based best workflow (LFQ), the top0-based best workflow (top0), the top3-based best workflow (top3) and the count-based best workflow (count) and the available true DEPs (T_DEP). These 5-expression matrix specific top-ranked workflows are locally top-ranked workflows.

We can see, 63% of the true DEPs (2373 out of 3773) were detected by the 5 globally top-ranked workflows consistently. There are 30% (1141 out of 3773) of the true DEPs missed by all the 5 globally top-ranked workflows. The remaining 7% true DEPs can be detected by at least one of the 5 globally top-ranked workflows. In comparison, only 37% of the true DEPs (1382 out of 3773) can be consistently detected by all the 5 locally top-ranked workflows. However, only 12% (468 out of 3773) of the true DEPs were never detected by the 5 locally top-ranked workflows. The remaining 51% of the true DEPs can be detected by at least 1 locally top-ranked workflow.

From the two groups comparisons we can see, combining top-ranked workflows has the potential to improve the accuracy in detection of DEPs. For example, if we combine the results from the 5 globally top-ranked workflows, the recall can be increased to about 70% (100% - 30%, without workflow integration, the highest recall achieved by the five top-ranked workflows is 0.715). When the 5 locally top-ranked workflows are integrated, as high as 88% (100% - 12%, without workflow integration, the highest recall achieved by the 5 top-ranked workflows is 0.755) of the recall could be achieved.

Comparison of top-ranked single workflows and top-ranked ensemble inference workflows

To check whether the ensemble inference can improve the DEA performance, we compared the metric values obtained by the best single workflows and the best ensemble inference workflows under different settings (see Fig. 5a in our main text). The details of the top-ranked single workflows and ensemble inference workflows are shown in following supp. Tab. 4. For single workflows (the first 6 rows), the workflow names are in the format of [DEA tool]|[platform]|[expression matrix type]|[imputation]|[normalization]. For ensemble inference workflows, the workflow names are in the format of ensemble_[p-value integration method]||[composited workflows]. Hurdle model [26] was more frequently used for p-value integration compared to fisher and set operation, especially for the ens_multi-quant approaches (supp. Tab. 4).

The detail performance gains obtained by best ensemble inference workflows are shown in following supp. Tab. 5.

Cross-setting comparisons based on another two pairs of datasets

In supp. Fig. 6, we show the comparisons of the best single workflows of DDA settings (FG_DDA and MQ_DDA), the best DIA settings (DIANN_DIA and spt_DIA) and the best ens_multi-qaunt of DDA and DIA settings based on the dataset pairs (HEqe408_LFQ, HEqe408_DIA) (supp. Fig. 6 (a)) and (HYtims134_LFQ, HYtims134_DIA) (supp. Fig. 6 (b)). We can see, DIA settings (spt_DIA and DIANN_DIA) always work better than DDA settings (FG_DDA and MQ_DDA) where more TPs (true positives) while less FPs (false positives) were detected (see top 8 bars in supp. Fig. 6). For example, when testing on dataset pair (HEqe408_LFQ, HEqe408_DIA), DIANN_DIA (the best single workflow of it) detected 781 TPs which are about 2 times of the number of TPs detected by MQ_DDA (374) and FG_DDA (392). However, only 21 FPs were detected by DIANN_DIA comparing to FG_DDA's 27 and FG_DDA's 57. Similarly, for the dataset pair (HYtims134_LFQ, HYtims134_DIA) DIANN_DIA obtained 256 TPs and only 10 FPs which is better than MQ_DDA's 77 TPs and 21 FPs, and FG_DDA's 219 TPs and 18 FPs. However, spt_DIA detect more FPs than FG_DDA from the dataset pair (HEqe408_LFQ, HEqe408_DIA) and detected less TPs than FG_DDA from the dataset pair (HYtims134_LFQ, HYtims134_DIA). Thus, DIANN_DIA shows higher robustness in detection of DEPs.

When comparing the best ens_multi-quant workflows with those single workflows (bottom 8 bars in supp. Fig. 6), we can see, ens_multi-quant can further improve the number of TPs. Especially, from the dataset pair (HYtims134_LFQ, HYtims134_DIA) applying the ens_multi-quant under different settings, the TP numbers were nearly doubled. However, more FPs were also detected by the ens_multi-quant workflows. For example, comparing spt_DIA_ens with spt_DIA, 62 more TPs were detected together with 74 more FPs from (HEqe408_LFQ, HEqe408_DIA). Similarly, DIANN_DIA_ens detected 41 more TPs and 31 more FPs from (HEqe408_LFQ, HEqe408_DIA) compared to DIANN_DIA. We should pay attention to the false positives detected by the ens_multi-quant workflows.

In our main text, we compare the DDA settings with DIA settings based on the dataset pair (HYEtims735_LFQ, HYEtims735_DIA) and compare DDA settings with TMT settings based on the dataset pair (HYqfl683_LFQ, HYqfl683_TMT11 [27]), see our Fig. 5b-c. supp. Fig. 7 shows the Receiver Operating Characteristic (ROC) curves of the compared settings.

From supp. Fig. 7 we can see, higher pAUC(0.05) scores were obtained by DIA settings and TMT settings compared to DDA settings. Thus, we can get the similar conclusion that DIA settings and TMT settings work better than DDA settings. In addition, the ens_multi-quant sometimes decrease the pAUC(0.05) scores due to more FPs were detected and they were ranked higher than some TPs.

Cross-platform DEA workflow integration

As mentioned above, under a given setting, e.g., DIANN_DIA, integration of top-ranked single workflows can improve the DEA performance. For the workflow integrations strategies, e.g., ens_multi-quant and ens_topk, usually only one quantification platform is always involved. We may wonder whether the integration of workflows based on different quantification platforms, e.g.,

FragPipe and Maxquant, can further improve the DEA performance. The DDA dataset HYEtims735_LFQ and DIA dataset HYEtims735_DIA (similar to Fig. 5b) were used to evaluate this cross-platform workflow integration strategy.

We adopted a simple pooling strategy to test the cross-platform integration. We pool DEPs detected by top 1st workflows from different quantification platforms, i.e., pooling DEPs from top 1st workflow of FG_DDA (FG_DDA in Fig. 5b) and top 1st workflow of MQ_DDA (MQ_DDA in Fig. 5b) (we call this method "pool_DDA"), pooling DEPs from top 1st workflow of DIANN_DIA (DIANN_DIA in Fig. 5b) and top 1st workflow of spt_DIA(spt_DIA in Fig. 5b) ("pool_DIA") and pooling all of the DEPs detected by all these four top 1st workflows (FG_DDA + MQ_DDA + DIANN_DIA + spt_DIA, named as "pool_DDA_DIA"). The pooling is implemented by a simple voting method, where the pooled q-value equals the minimum q-value of the candidates and the pooled logFC value equals the one has the biggest absolute value among the candidates.

In supp. Fig. 8 and supp. Tab. 6, we show the comparison of pooling-based cross-platform integration methods (with prefix of "pool_") and the single-workflow (using setting names as method names) and ens_multi-quant methods (with postfix of "_ens"). We can see, with the simple pooling strategy, the integration of DEA results from different quantification platforms can improve the performance comparing to single workflows. The pooling methods can even work better than the ens_multi-quant methods. Especially, by integrating the DEPs from all the four platforms (refers to pool_DDA_DIA), the biggest number of TPs can be detected though more FPs also were detected. The results indicate that the integration of outputs from multi-quantification platforms has the potential to maximize the DEP coverage. However, for large proteomics projects, the running time will increase hugely when multiple quantifications are conducted. In our future work, we will explore the design of advanced approaches for heterogeneous quantification data analysis to maximize the DEP coverages or other downstream applications.

Running time comparisons

We used the HEqe408_DIA dataset to evaluate the running speed of top-ranked single workflows and top-ranked ensemble inference workflows under settings DIANN_DIA and spt_DIA. The running is implemented with our standalone tool and the raw outputs from quantification platforms DIA-NN and Spectrnaut 18 were used as inputs. Following supp. Tab. 7 show the results of the running speed comparison.

Optimal workflow recommendations

In the first subsection ("Recommendations and resources for selecting optimal workflows") of the Discussion section in our main text, we listed some recommendations of optimal workflows for each setting. Our recommendation is mainly based on the benchmarking results as we found that our benchmarking has good generalizability (LODOCV with average Spearman correlation coefficients exceed 0.56, Fig. 2c) and the optimality is predictable where higher than 0.84 of

average F1 score and average MCC scores were achieved in the workflow performance level classification. These predictions are stable, exhibiting low sensitivity to instrument types (only 6 out of 120 top ranked workflows show instrument sensitivities (Kruskal-Wallis test [28], Fig. 2d). We also consider the frequent patterns extracted from high-performing workflows where the inclusive of these frequent patterns possibly will lead to better performance (Fig. 3d). In addition, the workflow step option comparison results (Fig. 4b-e) were also used as clues. We explain our recommendation as below:

- For label-free DDA data quantified by FragPipe (i.e., setting FG_DDA), we recommend a workflow combining protein directLFQ intensity, no normalization, SeqKNN for MVI, and DEqMS or ROTS (or limma, if running time is a concern) for DEA.

This recommendation is based on our benchmarking result that the workflows "dlfq intensity + no normalization + SeqKNN MVI + DEqMS/ROTS/limma for DEA" were ranked at 2nd/5th/10th among available workflows for setting FG_DDA (Fig. 2e, Supplementary Data 2). Though the workflow "dlfq intensity + no normalization + missForest MVI + DEqMS" was ranked at 1st, the MVI method of missForest always runs quite slow and the improvement achieved by it comparing to the previous three are small (see Supplementary Data 2). From the frequent pattern mining, we also found that dlfq (SR=0.52), no normalization (SR=0.36), SeqKNN (SR=0.15) are the most frequently used expression matrix, normalization and MVI options. Though missForest (SR=0.13) was chosen by some top ranked workflows, e.g., top 1st, 7-9th, and it was ranked at 1st in option comparisons (see Fig. 4d), it runs much slower (Figure Fig. 3d, Supplementary Data 5). The imputation also shows less impact to workflow performance than expression matrix, normalization, and DEA tool. Thus, we fixed these three options, e.g., dlfq, no normalization and SeqKNN. limma, ROTS and DEqMS were frequently chosen as the DEA statistical tools by "H" workflows of FG_DDA (SR=0.249, SR=0.244 and SR=0.234 respectively, Supplementary Data 5). These three options were also chosen by top ranked workflows (DEqMS was chosen by 6 out of the top 10 workflows, and limma and ROTS were chosen by 1 and 3 out of the top 10 workflows). In the workflow step option comparison, DEqMS, limma and ROTS were ranked at 1st, 2nd, and 4th respectively. In addition, in FG_DDA's "H" workflows, as the most frequently used expression matrix, "dlfq" is coupled with DEqMS, ROTS and limma with SRs higher than 0.1 (Fig. 3d, Supplementary Data 5).

- For label-free DDA data quantified by Maxquant (i.e., setting MQ_DDA), we recommend a workflow combining protein directLFQ intensity, no normalization, Impseq for MVI, and DEqMS or limma for DEA.

This recommendation is mainly based on our benchmarking results that under setting MQ_DDA, the workflows "dlfq + no normalization + Imqseq + DEqMS/limma" were ranked at the top 2 positions (Fig. 2e, Supplementary Data 2). dlfq is also the most frequently used expression matrix type (SR=0.52) under setting MQ_DDA. Though normalization method lossf and Rlr obtained higher SR values (SR=0.265 for lossf and SR=0.260 for Rlr, and they were ranked at 1st and 2nd in Fig. 4c) than No_norm (SR=0.188, ranked at 4th in Fig. 4c), the normalization method No_norm was more frequently selected by top ranked "H" workflows (all top 5 workflows chose No_norm).

As to the MVI method, Impseq was not only frequently chosen by top ranked "H" workflows but also ranked 1st in Fig. 4d. Though the DEA tools DEqMS (SR=0.21) and limma (SR=0.24) have lower SR values than ROTS (SR=0.26), they were frequently chosen by those top-ranked workflows (6 times for DEqMS and 4 times for limma by top 10 workflows). The ROTS was ranked at 1st among the DEA statistical tools (Fig. 2e), but those "H" workflows applying ROTS were always ranked lower and it runs slower than limma and DEqMS. Thus, we suggest using DEqMS and limma instead of ROTS. Again, we can find several pairs of options, i.e., (dlfq, no normalization), (dlfq, limma), and (dlfq, DEqMS) are preferred in "H" workflows of MQ_DDA with SR=0.158, SR=0.119 and SR=0.107 respectively (Fig. 3d, Supplementary Data 5).

- For label-free DIA data quantified by DIA-NN (i.e., setting DIANN_DIA), we recommend a workflow combining protein directLFQ intensity, no normalization, MinDet[20] for MVI, and limma for the DEA (ROTS can be an alternative DEA method if running time is not considered).

Again, the recommendation is mainly based on the benchmarking results. Under setting DIANN_DIA, the workflow "dlfq + no normalization + MinDet + limma" worked the best (Fig. 2e). dlfq (SR=0.597), No_norm (SR=0.349) and MinDet (SR=0.127) obtained the highest SRs comparing to other options in their corresponding workflow steps. dlfq, No_norm and MinDet were also ranked 1st in Fig 2b-d. Though ROTS was ranked 1st in Fig. 2e, and it obtained higher SR (SR=0.419) than limma (SR=0.276), limma was chosen by top 4 workflows and it runs faster than ROTS. Thus, we suggest using limma as the default statistic tool but using ROTS as an alternative option if running time is not considered.

- For label-free DIA data quantified by Spectronaut (i.e., setting spt_DIA), we recommend a workflow combining directLFQ intensity, no normalization, Impseq for MVI, and ROTS for DEA.

Under setting spt_DIA, the workflow "dlfq + no normalization + Imqseq + ROTS" and workflow "dlfq + no normalization + MinDet + ROTS" in fact have the same average ranks (Supplementary Data 2), though the later one was shown to be ranked at 1st (Fig. 2e). Similarly, No_norm (SR=0.435) and ROTS (SR=0.648) were always the most popular normalization and statistical tool settings in "H" workflows of spt_DIA (Supplementary Data 5), and frequently appeared in top ranked "H" workflows (Supplementary Data 2). We suggest using dlfq as the default matrix type per the fact that it was frequently selected by top ranked workflows compared to LFQ (all the top 8 workflows chose dlfq instead of LFQ, see Supplementary Data 2), though LFQ has higher SR value (SR=0.483 for LFQ and SR=0.435 for dlfq) and was ranked at 1st in Fig. 2b. For the choosing of MVI, none of the MVI algorithms obtained SR higher than 0.1 (Supplementary Data 5). We suggest using Imqseq as it was ranked at 1st in Fig. 2d, and it was chosen by the top ranked workflows.

- For TMT data quantified by FragPipe (i.e., setting FG_TMT), we recommend a workflow combining TMT-Integrator abundance, no normalization, SeqKNN for MVI, and limma for DEA.

The workflow "TMT-Integrator abundance + no normalization + SeqKNN + limma" is the best under setting FG_TMT (supp. Fig. 2 (a)). Again, from the frequent pattern side, the TMT-

Integrator abundance (abd), No_norm and limma obtained higher SRs while none of the MVI algorithms obtained SR>0.1. From the workflow step option comparison side, abd, No_norm and limma were ranked at 1st in Fig. 4b, 4c and 4e. proDA was also popular but was not as popular as limma (SR of 0.27 vs. limma's SR of 0.43) and it run much slower than limma (Supplementary Data 5). For the choosing of MVI method, the SeqKNN was selected by the ranked 1st workflow, and it appeared on 3 out of the top 10 workflows (Supplementary Data 2).

- For TMT data quantified by Maxquant, we recommend a workflow combining reporter intensity, no normalization, bpca for MVI, and proDA (or limma, if running time is a consideration) for DEA.

For MQ_TMT, the best workflow is "report intensity + no normalization + bpca + proDA" according to our benchmarking (supp. Fig. 2 (b), Supplementary Data 2). The reporter intensity is the only available choice of expression matrix. No normalization obtained the highest SR of 0.56. No frequently used imputation method was found, so bpca is still recommended per the benchmarking results. Though proDA obtained the highest SR (0.38) and was chosen by the ranked 1st workflow (supp. Fig. 2 (b)), the second highest tool limma can be an alternative tool as it also obtained SR of 0.38 and it was ranked at 1st in the DEA option comparison (proDA was ranked at 2nd), and it runs faster than proDA at the same time (Fig. 4e).

- For expression matrices without acquisition platform or quantification information, we recommend a workflow combining no normalization, lossf, Rlr, center.median for normalization options, MinProb, SeqKNN, Impseq or MinDet for MVI, and limma or ROTS for DEA.

When we obtained expression matrices without acquisition and quantification platform, we recommend workflows mainly based on the frequent pattern and workflow step option comparison results. Among the normalization methods, no normalization, the two regression based method lossf and Rlr and center.median are good candidates. However, under this situation, we should check the data variance at first to confirm whether normalization is necessary. For imputation, those algorithms such as MinProb, SeqKNN, Impseq or MinDet are ranked higher under different settings (cross-setting ranking, see right heatmap of Fig. 4d). For the DEA tool, limma and ROTS are two priorities (Supplementary Data 5 and Supplementary Data 6).

- Label-free DIA and TMT are recommended for proteomics experiment design as they are more accurate, with lower missing rates, and having higher proteome coverages comparing to label-free DDA. However, we don't have data to compare between label-free DIA and TMT directly. The choice of label-free DIA or TMT in experiment design should consider the level of multiplexing required, the desired dynamic range, and the budget.

This recommendation is based on our cross-setting comparisons where DIA and TMT can detect much more TPs and less FPs comparing to DDA settings, see Fig. 5b-c and supp. Fig. 6.

Supplementary Note 2

Simple introduction of expression matrix types, normalization methods, imputation algorithms and differential expression analysis tools involved in our benchmarking

1 Expression Matrix types:

1.1 DDA LFQ (Data-dependent acquisition & Label-free quantification):

Count (Spectral Count)[29]**:** Spectral count is the number of PSMs (peptide-spectrum matches) in support of the protein.

top0[30]**:** All the peptides are aggregated for protein quantification.

top3[25]**:** The top 3 most intense peptides are aggregated for protein quantification.

MaxLFQ[25]**:** Multiple peptide ratios are merged to calculate optimal protein ratios between sample pairs.

directLFQ[24]**:** A ratio-centric method is employed for normalizing samples and determining protein intensities. This algorithm estimates protein abundances via aligning samples and ion traces by shifting them on top of each other in logarithmic space.

1.2 DIA LFQ (Data-independent acquisition & Label-free quantification):

top1[30]**:** The most intense ion is used for protein quantification.

top3[25]**:** The top 3 most intense ions are aggregated for protein quantification.

MaxLFQ[30]**:** Multiple ion ratios are merged to calculate optimal protein ratios between sample pairs.

directLFQ[24]**:** A ratio-centric method is employed for normalizing samples and determining protein intensities. This algorithm estimates protein abundances via aligning samples and ion traces by shifting them on top of each other in logarithmic space.

1.3 TMT FragPipe (Tandem mass tag & FragPipe):

Philosopher[11]**:** Protein intensities are estimated from sum of the top 3 most intense peptide abundances with Philosopher.

Ratio[11]**:** FragPipe reports ratio to the bridge/reference channel in each plex if specified, or a ratio to the average abundance within each plex (virtual reference approach).

Abundance[11]**:** Protein abundances are generated by converting (normalized) ratios back to the intensity (ion abundance) scale.

1.4 TMT Maxquant (Tandem mass tag & Maxquant):

Reporter[7]**:** Protein intensity is the total intensity of all the isotopic patterns in the label cluster.

2 Normalization methods (The rows in the input matrix represent the proteins/features whereas the columns are the samples.):

2.1 None: Normalization is not performed.

2.2 Feature (along the rows):

Sum[31]**:** Each intensity in the matrix is divided by the sum of the intensities in the corresponding row.

Max[31]**:** Each intensity in the matrix is divided by the maximum of the intensities in the corresponding row.

2.3 Sample (along the columns):

center.mean[31]**:** This algorithm normalizes the sample (column) intensities by subtracting the corresponding column means.

center.median[31]**:** This algorithm normalizes the sample (column) intensities by subtracting the corresponding column medians.

div.mean[31]**:** The sample (column) intensities are normalized by dividing the column means.

div.median[31]**:** The sample (column) intensities are normalized by dividing the column medians.

TIC (Total Ion Current Normalization)[32]**:** The sample (column) intensities are normalized by dividing the sum of the intensities in the corresponding column and then multiply by the median value of the column.

2.4 Distribution alignment:

Quantiles (Quantile Normalization)[31,33]**:** The goal of the quantile normalization is to make the distribution of protein intensities for each sample in a set of samples the same.

quantiles.robust (Robust Quantile Normalization)[31,33]**:** This algorithm is a modified version of quantile normalization, which allows weighting of samples and removal of extreme samples.

MBQN (Mean-Balanced Quantile Normalization)[34]**:** This algorithm is a modified version of quantile normalization and balances the mean intensity of proteins (rows) which are rank invariant or nearly rank invariant across samples (columns) before quantile normalization.

2.5 Variance stabilization:

Vsn (Variance Stabilization Normalization)[31,35]**:** Variance stabilization normalization is to transform values in a dataset by conducting affine transformation for systematic experimental factor calibration and generalized logarithm ($glog_2$) transformation for variance stabilization such that the variability of the values is not related to their mean value.

2.6 Regression:

Rlr (Robust Linear Regression Normalization)[17,36]**:** Log2 transformed data is normalized by robust linear regression, which employs a method known as iteratively reweighted least squares. This approach exhibits lower sensitivity to significant variations in small parts of the data. Consequently, compared to standard linear regression normalization, robust linear regression normalization demonstrates reduced susceptibility to outliers.

Lossf (Cyclic LOESS Normalization)[37,38]**:** Cyclic LOESS (LOcally Estimated Scatterplot Smoothing) is a cyclic normalization technique where pairs of data samples undergo MA transformation (with 'A' representing the median sample and 'M' being the difference of each sample compared to 'A') and normalization, one pair at a time. This process iterates through all sample pairs and is repeated three times.

3 Imputation algorithms:

3.1 None: Missing value imputation is not performed.

3.2 Single value:

MinDet (Deterministic Minimum Imputation)[39]: In the imputation of left-censored missing data using a deterministic minimal value approach, for a given expression dataset with n samples and p features, each sample's missing values are imputed with the minimum value recorded in that specific sample. This minimum value is calculated as the q-th quantile of the observed values within the same sample.

MinProb (Probabilistic Minimum Imputation)[20]: This algorithm performs the imputation of left-censored missing data by random draws from a Gaussian distribution centered to a minimal value. Considering an expression data matrix with n samples and p features, for each sample, the mean value of the Gaussian distribution is set to a minimal observed value in that sample. The minimal value is determined as the q-th quantile of observed values within that sample. Additionally, the standard deviation for this Gaussian distribution is estimated using the median of the standard deviations across all features.

Min[40]: The missing values in matrix are replaced by the smallest non-missing value in the data.

Zero[20]: The missing values in matrix are replaced by zero.

3.3 Local similarity:

KNN (K Nearest Neighbors Imputation)[41]: This algorithm employs the K-nearest neighbors approach in the protein space to fill in missing values.

SeqKNN (Sequential K Nearest Neighbors Imputation)[42]: SeqKNN, a sequential imputation method, addresses missing values in proteins starting from the one with the fewest missing values. It employs the K-Nearest Neighbors approach for this task and utilizes the values imputed in earlier steps for subsequent imputations.

Mice (Multivariate Imputation by Chained Equations)[43]: This algorithm implements a multiple imputation technique, repeatedly executing imputation cycles until a definitive value is assigned to each missing value. Initially, it starts with a placeholder value, which is then refined through linear regression in each iteration, generating a new imputed value for use in the next iteration.

missForest (Random Forest Imputation)[18]: missForest imputes missing values particularly in the case of mixed-type data using a random forest approach. It is capable of handling both continuous and categorical data, effectively addressing complex interactions and nonlinear relationships. Additionally, it provides an out-of-bag error estimate for the imputation.

QRILC (Quantile Regression Imputation of Left-Censored Data)[44]: This missing data imputation method addresses left-censored missing data by conducting random draws from a truncated distribution whose parameters are estimated through quantile regression.

nbavg (Average Neighbor Imputation)[31]: The Average Neighbor Imputation method is specifically tailored for datasets comprising fractions collected along a fractionation/separation gradient, like those in sub-cellular fractionation. This approach operates under the assumption that these fractions are sequentially ordered along the gradient and is invalid otherwise. For continuous datasets, NA value at the beginning and the end of the quantitation vectors are set to the lowest

observed value in the data or to a user defined value. Subsequently, if a missing value is situated between two non-missing values, it is imputed using the average of these directly adjacent neighbors.

GMS (Generalized Mass Spectrum Missing Peaks Imputation with Two-Step Lasso)[45]: This algorithm applies a Lasso (Least absolute shrinkage and selection operator) model to select subsets of detected peaks to impute the missing values using a two-step Lasso procedure.

3.4 Global structure:

bpca (Bayesian PCA Missing Value Imputation)[46]: This algorithm is an iterative method using a Bayesian PCA (Principal Component Analysis) to impute missing values.

MLE (Imputation based on Maximum Likelihood Estimation)[47]: This algorithm is maximum likelihood-based imputation method using the EM (Expectation Maximization) algorithm.

Impseq (Sequential Imputation of Missing Values)[48]: This method sequentially imputes missing values in an incomplete observation by minimizing the determinant of the covariance in the expanded data matrix. Following this, the observation is incorporated into the complete data matrix, and the algorithm proceeds to the next observation that contains missing values.

Impseqrob (Robust Sequential Imputation of Missing Values)[49]: This method is similar to Impseq but improved by plugging in robust estimators of location and scatter.

4 DEA tools:

4.1 Protein intensity:

ANOVA (Analysis of variance)[21]: ANOVA encompasses a group of linear statistical models that examine the differences among means. This method operates on the law of total variance, which divides the observed variance in a given variable into portions corresponding to various sources of variation.

DEP (Differential Enrichment analysis of Proteomics data)[14]: This algorithm performs a differential expression analysis based on protein-wise linear models, combined with empirical Bayes statistical methods. Tail area-based false discovery rates (FDRs) and local FDRs are then estimated using fdrtool[28].

DEqMS (Differential Expression analysis of quantitative Mass Spectrometry data)[15]: DEqMS is a robust method designed for analyzing differential protein expression in mass spectrometry datasets. This method considers the inherent dependence between protein variance and the number of PSMs or peptides employed in quantification. As a result, it offers a more precise estimation of protein variance.

Limma (Linear Models for Microarray)[37]: limma uses the linear models to assess differential expression, combined with empirical Bayes statistics for refinement of the results.

MSstats[50]: MSstats constructs either a fixed effects model or a mixed effects model, tailoring the model to suit the specific experimental setup and to each individual protein. This model is then utilized to identify proteins that show differential abundance.

proDA[51]: proDA is a method designed for conducting statistical tests to detect differential abundance of proteins. It uniquely handles missing values by adopting an intensity-dependent probabilistic approach. Built upon linear models, proDA is apt for intricate experimental designs.

Additionally, it enhances statistical power in cases of small sample sizes through the application of variance moderation.

ROTS (Reproducibility-Optimized Test Statistic)[13]: ROTS modifies a t-statistic to align with the intrinsic characteristics of the data, offering a ranking of features based on their statistical significance in showcasing differential expression across two groups.

SAM (Significance analysis of microarrays)[52]: SAM is employed to identify differentially expressed proteins. This algorithm allocates a score to each protein, which is determined by comparing the differences in expression levels against the standard deviation. Additionally, it offers an estimate of the FDR that is based on permutation analysis.

***t*-test:** The Student's *t*-test is a hypothesis test that evaluates whether the means from two normally distributed populations are equal.

4.2 Protein spectra counts:

edgeR (empirical analysis of digital gene expression in R)[53]: edgeR is a tool designed for analyzing differential expression in replicated count data. It utilizes an over-dispersed Poisson model to address both biological and technical variability. To enhance the reliability of its findings, edgeR employs Empirical Bayes methods for moderating the extent of overdispersion across different transcripts. This approach is effective even with minimal replication, as long as there is at least one replicated phenotype or experimental condition. Moreover, the utility of edgeR extends beyond sequencing data and can be applied to other domains, such as proteome peptide count data.

plgem (power law global error model)[54]: plgem introduces a novel technique for identifying differentially expressed genes. This method involves a specialized statistic that directly applies measurement spread estimates derived from the model. It also includes a hypothesis testing algorithm that is based on resampling methods.

beta-binomial[55]: This method utilizes a beta-binomial sampling model that correctly incorporates both between-library variability and the within-library variability to identify the differential expression genes.

Description of benchmarking datasets

Three types of benchmarking datasets were used such as label-free DDA datasets (DDA), label-free DIA datasets (DIA) and TMT datasets (TMT). Our Table 1 and supp9. Tab1 in Supplementary Data 9 list the details of each dataset. These datasets are obtained from 14 proteomics projects (see ProteomeXchange IDs in Table 1 and supp9. Tab1)

The project with ID PXD028735 (https://www.ebi.ac.uk/pride/archive/projects/PXD028735) was designed to validate data analysis pipelines on modern day acquisition strategies in proteomics using SCIEX TripleTOF5600 and 6600+, Orbitrap QE-HFX, Waters Synapt GS-Si and Synapt XS and Bruker timsTOF Pro [23]. However, we only chose the data generated by the four popular machines, e.g., SCIEX TripleTOF5600 and 6600+, Orbitrap QE-HFX and Bruker timsTOF Pro, for workflow evaluation. Another reason of choosing only the four machines is to avoid the benchmarking result will be dominated by the same project, which can help enhance the

generalizability of our benchmarking. We generated 4 DDA dataset from this project including "HYE5600735_LFQ" generated by SCIEX TripleTOF5600, "HYE6600735_LFQ" generated by SCIEX TripleTOF6600+, "HYEqe735_LFQ" generated by Orbitrap QE-HFX and "HYEtims735_LFQ" generated by Bruker timsTOF Pro. Each of the dataset contains two groups of samples, where sample A contains 5% of Ecoli weight for weight (w/w), 30% Yeast and 65% Human while sample B contains 20% Ecoli, 15% Yeast and 65% Human. So, comparing sample B and sample A (contrast conditionB-conditionA), both Ecoli and Yeast proteins are differentially expressed proteins (DEPs) with log2(fold change) (logFC) of -2 and 1 respectively while human proteins are non-differentially expressed proteins (non-DEPs). Four contrasts of DDA data were available from this project. We also chose the DIA data generated by the Bruker timsTOF Pro to evaluate workflows for analyzing DIA data, namely the HYEtims735_DIA dataset with 1 contrast of conditionB-conditionA (similarly, human proteins are non-DEPs, and Ecoli and Yeast proteins are DEPs with logFC of 1 and -2).

The project with ID PXD036134 (https://www.ebi.ac.uk/pride/archive/projects/PXD036134) was designed as additional data for evaluation mass spectrometry downstream analysis pipeline (MS-DAP) [2]. This is a two-proteome spike-in series created by mixing 50ng HeLa per sample with 12.5ng (conditionB), 15.625 (conditionC) or 18.75 Yeast (conditionD) respectively. The TimsTof Pro2 machine was used for peptide detection with both DDA and DIA modes. Two datasets were generated such as DDA dataset "HYtims134_LFQ" and DIA dataset "HYtims134_DIA". Three contrasts are available: conditionD-conditionB, conditionD-conditionC and conditionC-conditionB where the Yeast proteins are DEPs with logFC of log2(1.5), log2(1.2) and log2(1.25) respectively.

The project with ID of PXD021425 (https://www.ebi.ac.uk/pride/archive/projects/PXD021425) was designed to generate spike-in data set for evaluating peptide identity propagation (PIP) [56]. The machine timsTOF Pro was used for data generation. In this project, spike-in samples were prepared by mixing HeLa sample with 0%, 3%, 4.5%, 6%, 7.5% or 9% (wt/wt) of E. coli sample (n=3). We only used the mixtures of Hela with 4.5% (conditionA), 6% (conditionB) and 9% (conditionC) (wt/wt) of Ecoli for generating DDA dataset "HEtims425_LFQ". Three contrasts are available including conditionC-conditionA, conditionC-conditionB and conditionB-conditionA where Ecoli proteins are true DEPs with logFCs of 1, log2(1.5) and log2(4/3) respectively.

The project with ID of PDC000006 (https://proteomic.datacommons.cancer.gov/pdc/TechnologyAdvancementStudies/) is designed as study 6 of the Clinical Proteomic Tumor Analysis Consortium (CPTAC) program where an older machine LTQ-Orbitrap was used [57]. Five concentrations of 0.25 (A), 0.74 (B), 2.2 (C), 6.7 (D) or 20 (E) fmol/µL of 48 UPS1 proteins were spiked in yeast proteins and 3 replicates were analyzed for each concentration. We generated the DDA dataset "YUltq006_LFQ" with data from this project. There are 10 possible contrasts when combining 2 out of the 5 conditions. We chose 2 out of the 10 contrasts (conditionE-conditionB, logFC=log2(80); conditionD-conditionC, logFC=log2(67/22)) due to the old machine and avoidance of performance domination by a specific project.

The project with ID of PXD002099 (https://www.ebi.ac.uk/pride/archive/projects/PXD002099) is

also produced by an old machine LTQ Orbitrap Velos. We generated dataset “YUltq099_LFQ” from this project. Five conditions of samples are available with 48 human UPS1 proteins with concentrations of 2 (A), 4 (B), 10 (C), 25 (D), and 50 (E) fmol/µL were added to trypsin-digested soluble yeast proteins [58]. Similarly, of the 10 contrasts, we only chose 2 out of the 10 contrasts for workflow evaluation (conditionE-conditionC with logFC=log2(5); conditionD-conditionB with logFC=log2(25/4)).

The project with ID of PXD001819 (https://www.ebi.ac.uk/pride/archive/projects/PXD001819) was designed to benchmark several label-free quantitative workflows, involving different software packages developed in recent years [59]. In this project, 48 UPS1 proteins at concentrations 0.05 (A), 0.125 (B), 0.25 (C), 0.5 (D), 2.5 (E), 5 (F), 12.5 (G), 25 (H) or 50 (I) fmol/ml were mixed with yeast lysate and proteins were detected by LTQ Orbitrap Velos. We chose 3 (D-A with logFC=log2(10); F-C with logFC=log2(20); I-H with logFC=log2(2)) out of 36 possible contrasts for DDA workflow evaluation.

The project with ID of PXD018408 (https://proteomecentral.proteomexchange.org/cgi/GetDataset?ID=PXD018408) was designed to establish guidelines for data acquisition, statistical approach, and replicate numbers for accurate quantification [1]. The machine Q Exactive was used for proteomics data acquisition with both DDA and DIA modes. The datasets “HEqe408_LFQ” (DDA) and “HEqe408_DIA” were generated from this project. In the experiment, 100 µg (A) or 200 µg (B) of the E. coli lysate was spiked into 400 µg of the human lysate (HEK293 cell lysate) and then 8 replicates. One contrast (B-A) for each data type is available with logFC=1.

The project with ID of PXD007683 (https://www.ebi.ac.uk/pride/archive/projects/PXD007683) was designed to test the ability of a tandem mass tagging (TMT) method (TMT11plex-SPS-MS3) and a label- free quantitation method (LFQ DDA) in detecting differentially expressed proteins [27]. In this project, Yeast lysate was spiked into human lysate to 10% of total protein concentration (1× group) (A, 3replicates), 5% (2× group) (B, 4 replicates), and 3.3% (3× group) (C, 4 replicates) for a total of 11 samples. Three contrasts are available such as C-A (logFC=-log2(3)), C-B (logFC=-log2(5/3.3)) and B-A (logFC=-1) for each method. The machine Orbitrap Fusion Lumos was used for peptide detection and generated both LFQ DDA data (dataset “HYqfl683_LFQ”) and TMT data (“HYqfl683_TMT11”).

The project with ID of PXD014777 (https://www.ebi.ac.uk/pride/archive/projects/PXD014777) was designed to evaluate the ion mobility enhanced MaxQuant software [7]. In the mixed species experiments, tryptic protein digests of H. sapiens (HeLa), S. cerevisiae (Promega) and E.coli (Waters) were mixed in two different experiments leading to a ratio of 1:1 (HeLa), 1:2 (S. cerevisiae, Yeast) and 1:4 (E.coli, Ecoli) between the two samples (with only one contrast B-A with Yeast and Ecoli proteins are true DEPs and the logFC of the Yeast protein is 1 while the logFC of the Ecoli proteins is 2). The machine timsTOF Pro was used to detect peptides under DDA mode, which produced our DDA dataset “HYEtims777_LFQ”.

The project with ID of PXD034709 (https://www.iprox.cn/page/project.html?id=IPX0004576000) was designed to benchmark commonly used software suites and analysis workflows for DIA

proteomics and phosphoproteomics [60]. In this project, each replicate of mouse membrane protein digest was spiked into one replicate of yeast protein digest to generate one reference (containing 20% mouse membrane proteome) and six samples for comparison (containing 5% (A), 10% (B), 13% (D), 20% (C), 30% (E) and 40% (F) mouse membrane proteome). The peptides were detected with the timsTOF Pro. We generated the DIA dataset “MYtims709_DIA”. We selected the contrasts C-B (logFC=1), C-A (logFC=2) and E-C (logFC=log2(1.5)) for DIA workflow evaluation.

The project with ID of PXD026600 (https://proteomecentral.proteomexchange.org/cgi/GetDataset?ID=PXD026600) was designed to compare workflows for acquisition and treatment of proteomic data analyzed in Data Independent Acquisition (DIA) mode [61]. The project data was generated by spike-in the 48 human proteins of UPS1 (Sigma) in a whole cell extract of E.coli at 8 different concentrations ranging from 0.1 to 50 fmol of UPS1/ug of E.coli (A:0.1, B:0.25, C:1, D:2.5, E:5, F:10, G:25, H:50). Each sample has been trypsin-digested analyzed in triplicate on an Orbitrap Fusion instrument (Thermo) operating in DIA mode with four different sizes of precursor windows (narrow, wide, mixed or overlapped). We chose data acquired by using the precursor windows of narrow and wide to generate our datasets “HEof_n600_DIA” and “HEof_w600_DIA”. For each dataset, 3 contrast were used for workflow benchmarking. The contrasts E-C (logFC=log2(5)), G-F (logFC=log2(2.5)) and E-B (logFC=log2(20)) were chosen from “HEof_n600_DIA” while the contrasts E-A (logFC=log2(50)), A-B (logFC=log2(2.5)) and F-C (logFC=log2(10)) were chosen from “HEof_w600_DIA”.

The project with ID PXD019777 (https://www.ebi.ac.uk/pride/archive/projects/PXD019777) was designed to allow direct comparison of DDA and DIA for label-free proteomics analyses. This project uses the same spike-in samples as above project PXD021425 [56] where the spike-in samples were prepared by mixing HeLa sample with 0%, 3%, 4.5%, 6%, 7.5% or 9% (wt/wt) of E. coli sample (n=3). The peptides were detected by a Q Exactive HF machine with DIA mode. Again, we used the mixtures of Hela with 4.5% (conditionA), 6% (conditionB) and 9% (conditionC) (wt/wt) of Ecoli for generating DDA dataset “HEqe777_DIA”. Three contrasts including C-A, C-B and B-A where Ecoli proteins are true DEPs with logFCs of 1, log2(1.5) and log2(4/3) respectively were used for workflow evaluation.

The project with ID of PXD013277 (https://proteomecentral.proteomexchange.org/cgi/GetDataset?ID=PXD013277) was designed to evaluate the DEA tools for differential expression analysis of TMT data [15]. In this project, samples with different spiked in amounts of E. coli protein extract (3 replicates with 7.5 µg (A), 4 with 15 µg (B) and 3 with 45 ug (C)) in MCF-7 background (70 µg of protein extract) were prepared. The peptides were labeled with TMT10plex and detected by a Q exactive machine, MS2 level quantification was implemented. We generated TMT dataset “HEqe277_TMT10” from this project. Three contrasts were used for TMT workflow evaluation including C-A with logFC=log2(6), C-B with logFC=log2(3) and B-A with logFC=1.

The project with ID of PXD020815 (https://www.ebi.ac.uk/pride/archive/projects/PXD020815) was designed to challenge the mass spectrometer to measure a range of protein abundance ratios in a background of potential interference [62]. In this project, the HYpro16 standard consists of

TMTpro-labeled human peptides at a 1:1 (A) ratio across all channels were mixed with TMTpro-labeled yeast peptides in triplicate at 20:1 (E), 10:1 (D), 4:1 (C), and 2:1 (B) ratios. The peptides were detected by a FAIMSpro-equipped Orbitrap Fusion Lumos machine and with different data acquisition methods such as hrMS2, SPS-MS3, RTS-MS3. We generated three datasets namely “HYms2faims815_TMT16”, “HYsps2815_TMT16” and “HYms2815_TMT16” based on three machine settings e.g., hrMS2 acquisition with FAIMSpro module (HYms2faims815_TMT16), SPS-MS3 acquisition without FAIMSpro module “HYsps2815_TMT16” and hrMS2 acquisition without FAIMSpro module “HYms2815_TMT16” respectively. For other settings, e.g., RTS-MS3 acquisition, we obtained some errors from with platform Maxquant. For dataset “HYms2faims815_TMT16”, we used the contrasts A-B (logFC=-1), B-D (logFC=-log2(5)), and C-E (logFC=-log2(5)) for workflow evaluation. For dataset, HYsps2815_TMT16, contrasts B-C (logFC=-1), B-E (logFC=-log2(10)), and A-C (logFC=-2), and for HYms2815_TMT16, contrasts A-D (logFC=-log2(10)), A-E (logFC=-log2(20)), and D-E (logFC=-1) were used for workflow evaluation.

The raw data of above datasets were downloaded with the software FileZilla (3.46.3).

Parameter settings of quantification platforms

FragPipe for quantifying DDA LFQ data

The software config of FragPipe are as follows:

FragPipe v20.0; MSFragger-3.8; IonQuant-1.9.8; Philosopher-5.0.0; python 3.11.4; java 20.0.2; Windows 10, Architecture AMD64.

When FragPipe is used to quantify DDA LFQ data, the parameter settings are as follow

a.) workflow sheet. workflow “LFQ-MBR” is loaded in the.

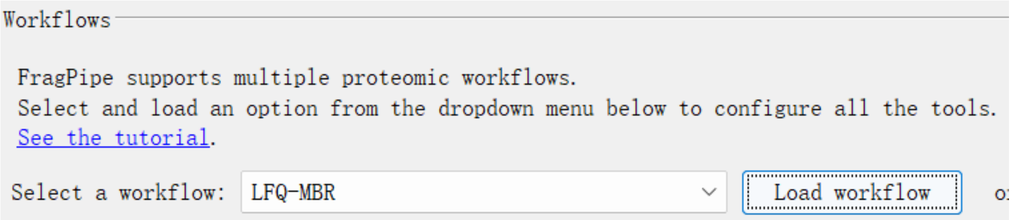

b.) Database sheet. We add the downloaded database from uniport (the detailed database information is listed in supp9. Tab1 of Supplementary Data 9). We add decoy and contaminants with the “Add decoys” button. The prefix is “rev_”

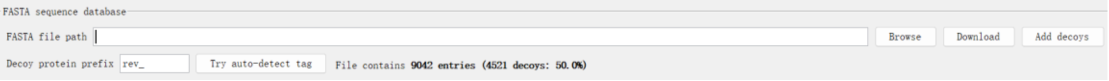

c.) MSFragger sheet.

Peak Matching: Precursor mass tolerance *-20 PPM – 20 PPM*; Fragment mass tolerance *20PPM*; Calibration and Optimization *Mass calibration, parameter optimization*; Isotope error *0/1/2*

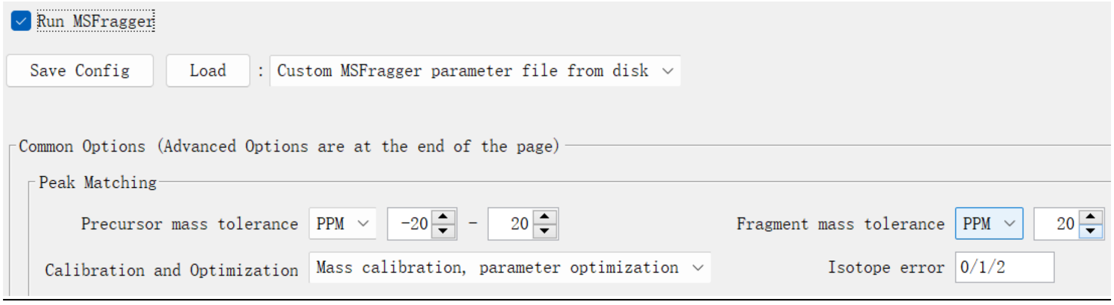

Protein Digestion: Cleavage *ENZYMATIC*; Clip N-term M *True*; Enzyme name 1 *stricttrypsin*; Load rules *stricttrypsin*; Cuts *KR*; Missed cleavages 1 *2*; Sense 1 *C*; Peptide length *7-50*; peptide mass range *500-5000*; split database *1*

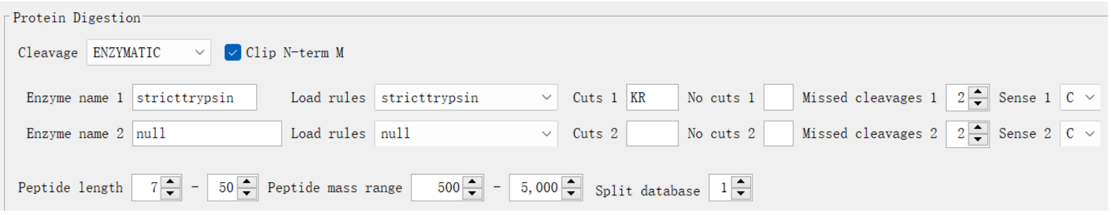

Modifications: Max variable mods on a peptide *3*; Max combinations *5000*; site enabled *M*, *[^*; all of the fixed modifications are selected.

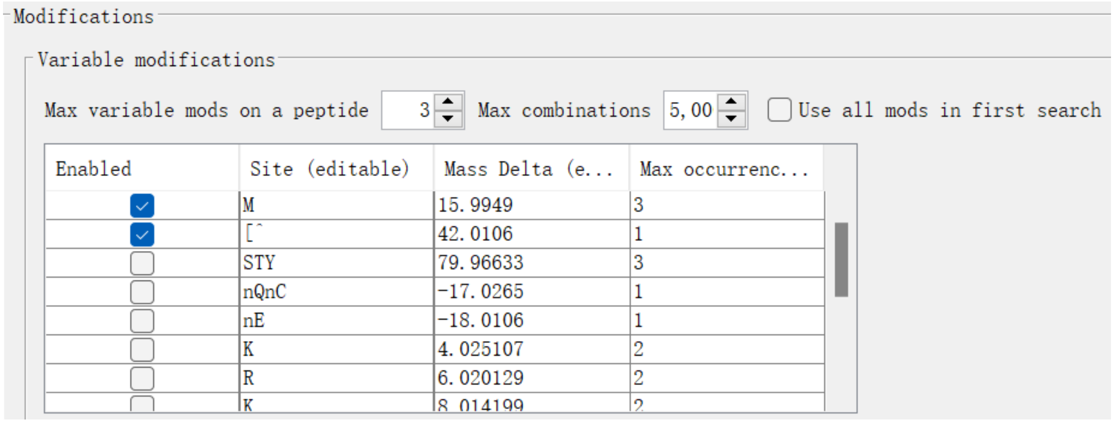

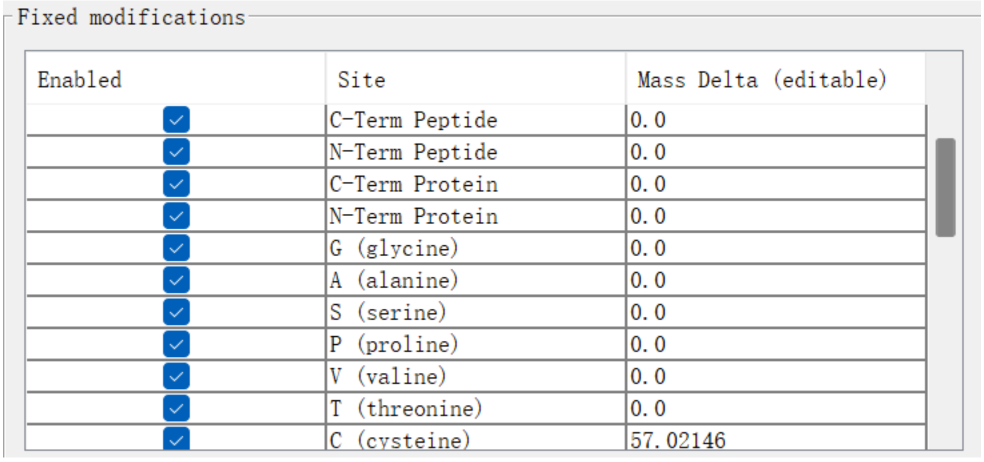

Spectral Processing: Activation Type Filter *all*; Min Peaks *15*; Clear m/z range *0 – 0*; Use top N peaks *150*; Min ratio *0.01*; Remove precursor peak *Only peak with precursor*

charge; removal m/z range *-1.5 – 1.5*

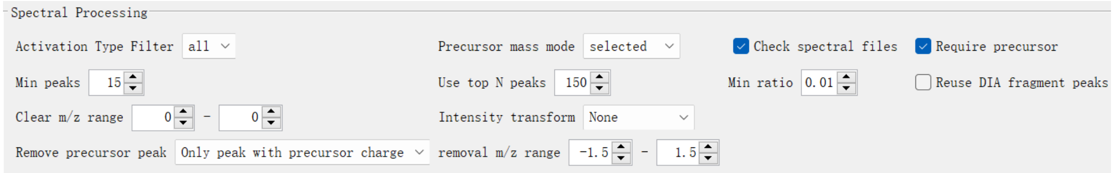

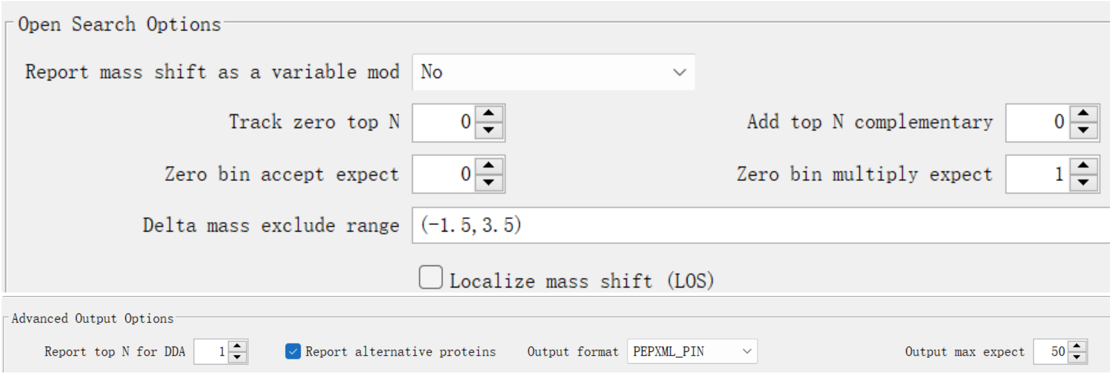

Advanced Peak Matching Options: Min frags modeling *2*; Min matched frags *4*; Max fragment charge *2*; Deisotope *Yes*; Fragment ion series *b,y*; Deneutralloss *Yes*; Precursor true tolerance *20PPM*

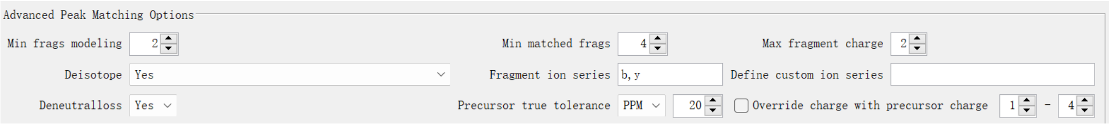

d.) Validation sheet

Run Validation Tools *Yes*; Run MSBooster *Yes*; Predict RT *Yes*; Predict spectra *Yes*

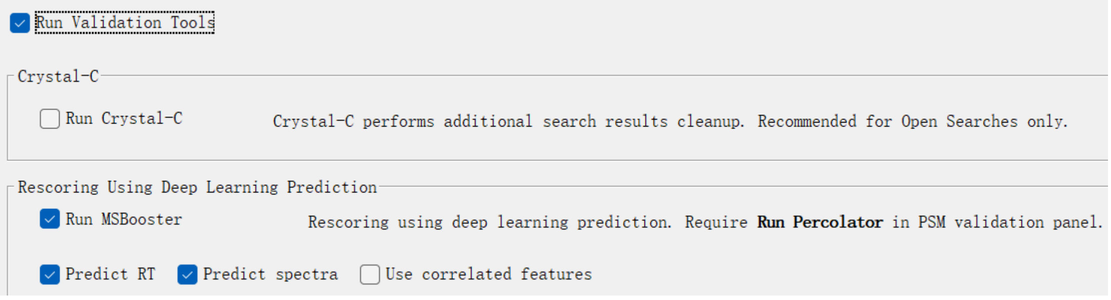

Run PSM validation *Yes*; Run Percolator *Yes*; Min probability *0.5*; Cmd line opts *--only-psms --no-terminate --post-processing-tdc*

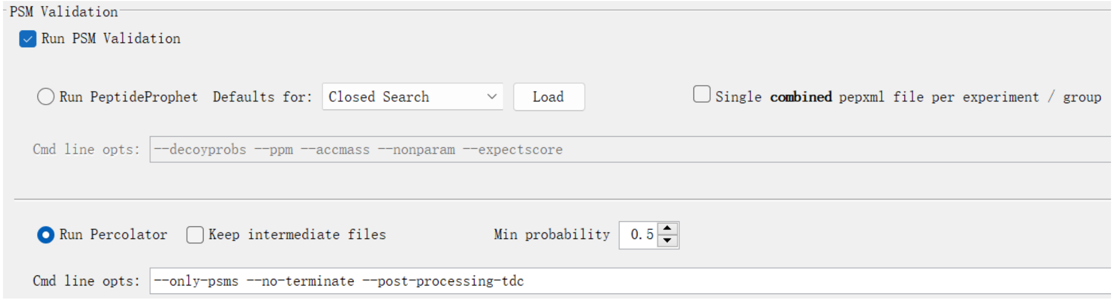

Protein Inference: Run ProteinProphet *Yes*; Cmd line opts *--maxppmdiff 2000000*; Generate reports *Yes*; Filter *--sequential --prot 0.01*; Generate MSstats files *Yes*; Generate peptide-level summary *Yes*; Generate protein-level summary *Yes*

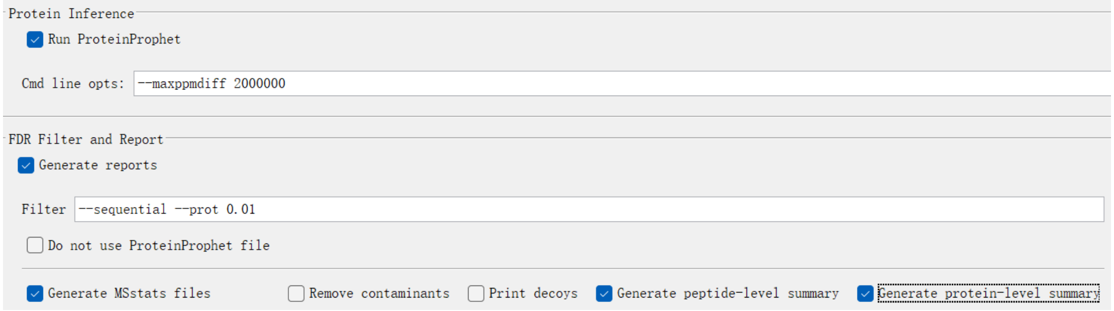

e.) Quant (MS1) sheet:

RUN MS1 quant *Yes*; LFQ *Yes*; Add MaxLFQ *Yes*; MaxLFQ min ions *2*; Match between runs(MBR) *Yes*; Normalize intensity across runs *Yes*; Peptide-protein uniqueness *unique+razor*

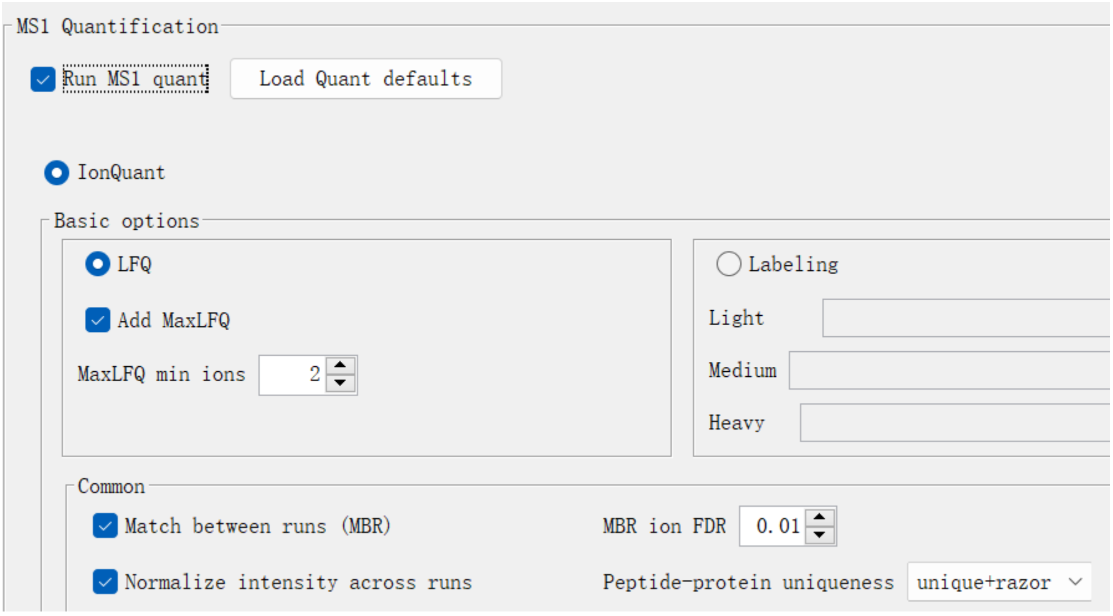

Advanced options: Min scans *3*; Min isotopes *2*; m/z tolerance (ppm) *10*; RT tolerance (minutes) *0.4*; IM tolerance (1/k0) *0.05*; MBR RT tolerance (minutes) *1*; MBR IM tolerance (1/k0) *0.05*; MBR peptide FDR *1*; MBR min correlation *0*; MBR top runs *10*; MBR protein FDR *1*

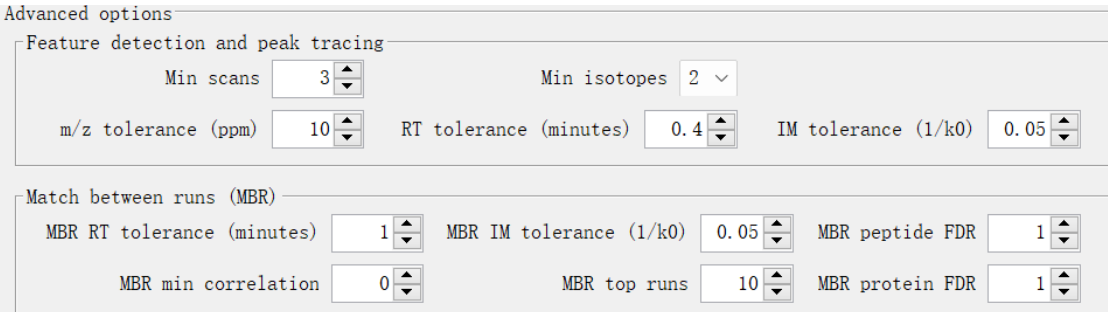

Intensity options: Top N ions *0* (we change the value to 3 when we conduct top3 quantification); Min freq *0*; Min site localization probability *0.75*

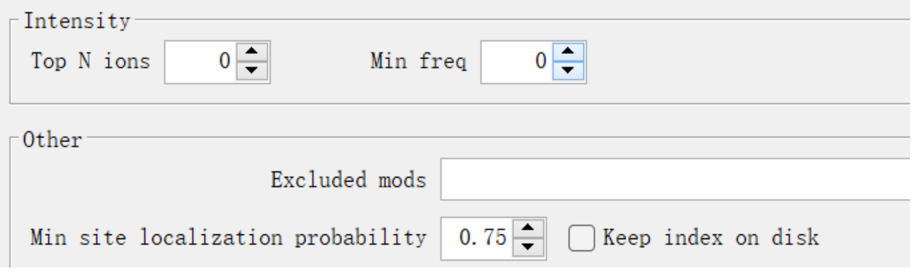

Maxquant for quantifying DDA LFQ data

The MaxQuant_2.1.0.0 was also used for quantifying DDA LFQ data. The parameter settings are as follows:

a.) Type *Standard*

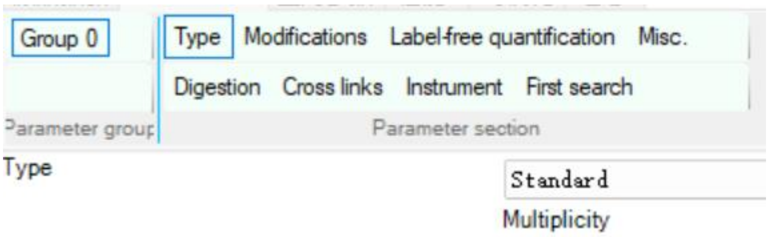

b.) Modifications: Variable modifications *Oxidation (M)* *Acetyl (Protein N-term)*; Fixed modifications *Carbamidomethyl (C)*; Max. number of modifications per peptide *5*

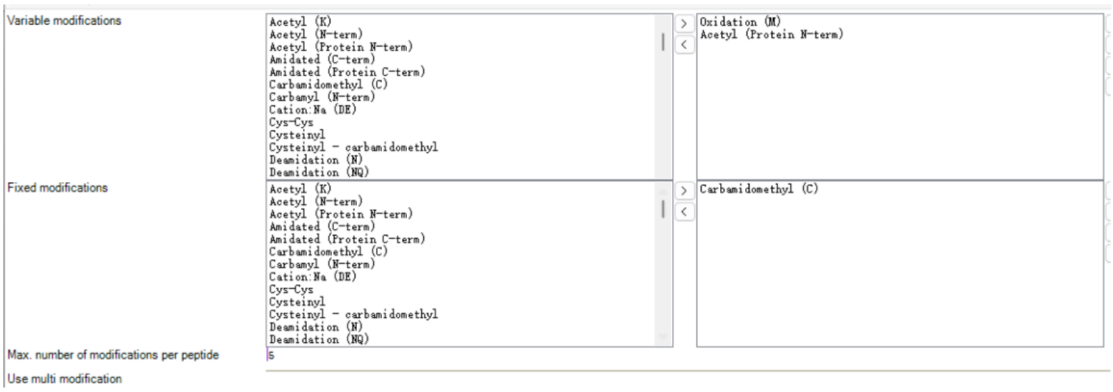

Label-free quantification *LFQ*; LFQ min. ratio count *2*; Normalization type *Classic*; Fast LFQ *Yes*; LFQ min. number of neighbors *3*; LFQ average number of neighbors *6*

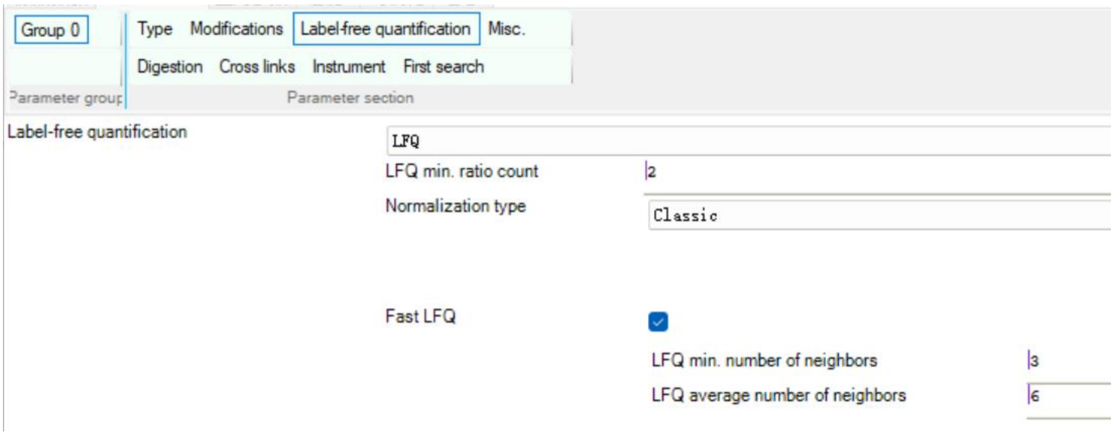

Digestion Digestion mode *Specific*; Enzyme *Trypsin/P*; Max. missed cleavages *2*

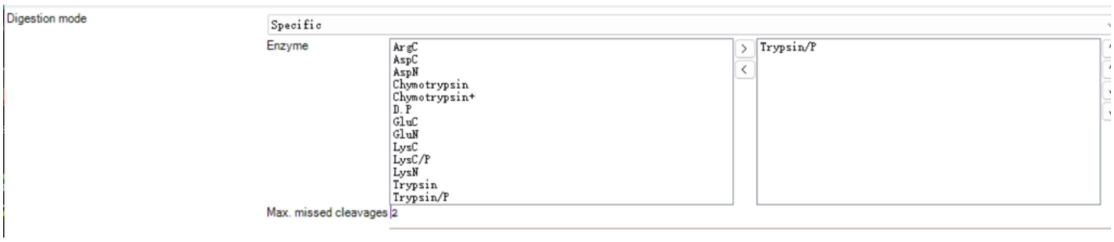

Instrument For datasets generated by Orbitrap QE-HFX (HYEqe735_LFQ), LTQ-Orbitrap (YUltq006_LFQ), LTQ Orbitrap Velos (YUltq099_LFQ, YUltq819_LFQ), Q Exactive (HEqe408_LFQ, HEqe777_DIA, HEqe408_DIA, HEqe277_TMT10), Orbitrap Fusion Lumos (HYqfl683_LFQ, HYms2faims815_TMT16, HYsps2815_TMT16, HYms2815_TMT16), Orbitrap Fusion ETD (HEof_n600_DIA, HEof_w600_DIA)

the instrument type is set as ***Orbitrap***; The remain settings are shown as following screenshots

Orbitrap	
First search peptide tolerance	20
Main search peptide tolerance	4.5
Peptide tolerance unit	ppm
Individual peptide mass tolerance	☑
Isotope match tolerance	2
Isotope match tolerance unit	ppm
Centroid match tolerance	8
Centroid match tolerance unit	ppm
Centroid half width	35
Centroid half width unit	ppm
Time valley factor	1.4
Isotope valley factor	1.2
Isotope time correlation	0.6
Theoretical isotope correlation	0.6
Recalibration unit	ppm
Use MS1 centroids	☐
Use MS2 centroids	☐
Intensity dependent calibration	☐
Min. peak length	2
Min. DIA peak length	1
Max. charge	7
Min score for recalibration	70
Cut peaks	☑
Gap scans	1
Advanced peak splitting	☐
Intensity threshold MS1	0
Intensity threshold MS2	0
Check mass deficit	☑
Intensity determination	Value at maximum
Centroid Position	Gaussian
DIA initial precursor mass tolerance [ppm]	20
DIA initial fragment mass tolerance [ppm]	20
DIA corr. threshold for feature clustering	0.85

DIA max. train instances	1000000
DIA LFQ ratio type	Median
DIA XGBoost Base Score	0.4
DIA XGBoost Sub Sample	0.9
DIA XGBoost learning objective	Binary logistic raw
DIA XGBoost Min child weight	9
DIA XGBoost Maximum Tree Depth	12
DIA XGBoost Estimators	580
DIA XGBoost Gamma	0.9
DIA XGBoost Max Delta Step	3
DIA no ML	☐
DIA only isos for recalibration	☑
DIA min. peaks for recalibration	5
DIA max. fragment charge	3
DIA use frag intensities for ML	
DIA use frag masses for ML	
DIA RT prediction	
DIA RT prediction second round	
DIA permute RT	
DIA permute CCS	
DIA prec. mass tol. for feat. clustering [ppm]	2
DIA frag. mass tol. for feat. clustering [ppm]	2
DIA score N	7
DIA min. score	1.99
DIA quant method	Mixed, LFQ split
DIA feature quant method	Sum
DIA top N fragments for quant	10
DIA top msms intensity quantile for quant	0.85
DIA min. msms intensity for quant	0
DIA precursor filter type	None
DIA min. fragment overlap score	1
DIA min. precursor score	0.5
DIA min. profile correlation	0
DIA global ML	☑
DIA adaptive mass accuracy	☐
DIA mass window factor	3.3
DIA background subtraction	☐
DIA background subtraction quantile	0.5
DIA background subtraction factor	4
DIA transfer q-value	0.3

For datasets generated by TimsToF pro (HYEtims735_LFQ, HYtims134_LFQ, HEtims425_LFQ , HYEtims777_LFQ), the instrument type is set as *Bruker TIMS*; the remain parameters are shown in following screenshot:

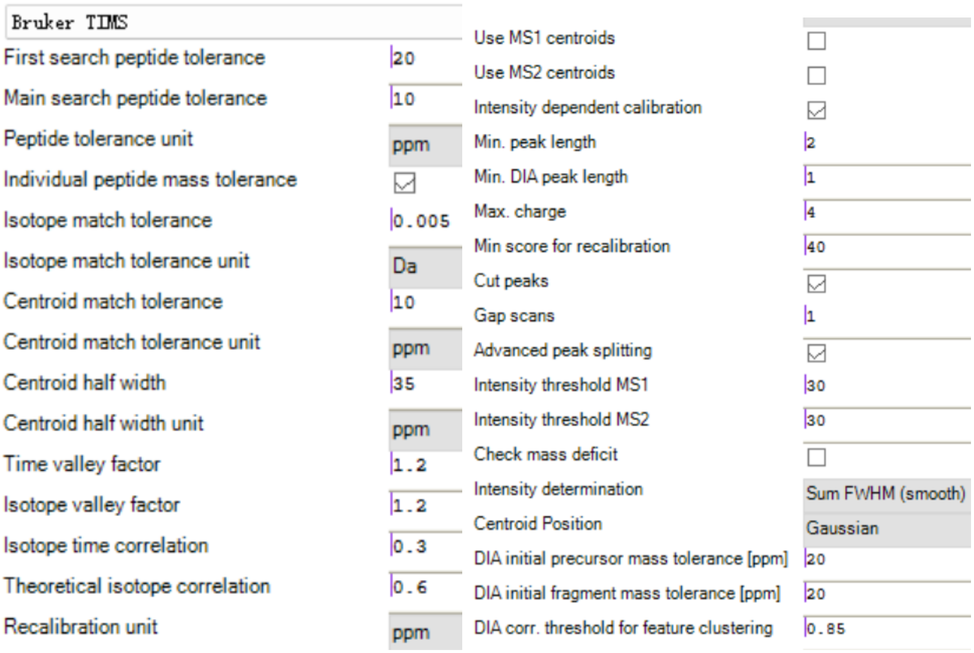

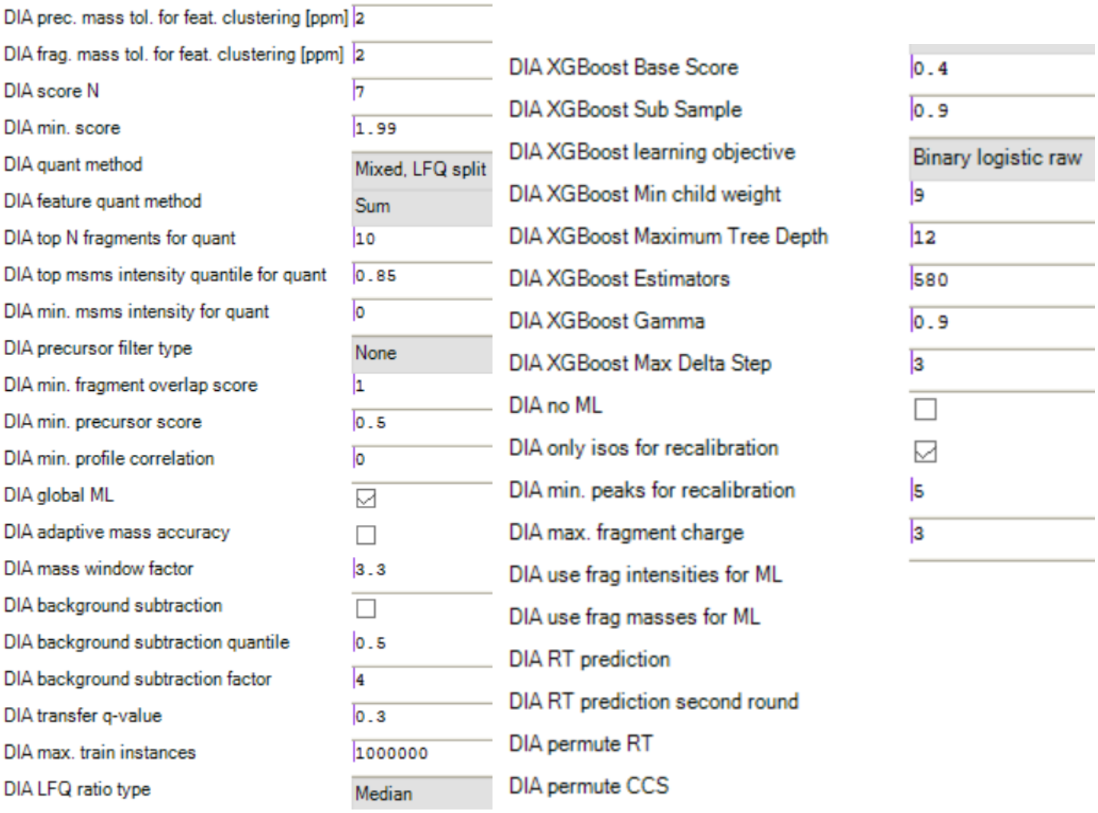

For datasets (HYE5600735_LFQ, HYE6600735_LFQ) generated by instruments SCIEX Triple TOF5600 and SCIEX Triple TOF6600, the instrument type is set as *Sciex Q-TOF*; remain parameters are set as shown in following screenshots:

Sciex Q-TOF	
First search peptide tolerance	20
Main search peptide tolerance	10
Peptide tolerance unit	ppm
Individual peptide mass tolerance	☑
Isotope match tolerance	0.005
Isotope match tolerance unit	Da
Centroid match tolerance	10
Centroid match tolerance unit	ppm
Centroid half width	35
Centroid half width unit	ppm
Time valley factor	1.2
Isotope valley factor	1.2
Isotope time correlation	0.3
Theoretical isotope correlation	0.6
Recalibration unit	ppm

Use MS1 centroids	☐
Use MS2 centroids	☐
Intensity dependent calibration	☑
Min. peak length	2
Min. DIA peak length	2
Max. charge	4
Min score for recalibration	40
Cut peaks	☑
Gap scans	1
Advanced peak splitting	☑
Intensity threshold MS1	100
Intensity threshold MS2	20
Check mass deficit	☐
Intensity determination	Sum FWHM (smooth)
Centroid Position	Gaussian
DIA initial precursor mass tolerance [ppm]	20
DIA initial fragment mass tolerance [ppm]	20
DIA corr. threshold for feature clustering	0.85

DIA prec. mass tol. for feat. clustering [ppm]	2
DIA frag. mass tol. for feat. clustering [ppm]	2
DIA score N	7
DIA min. score	1.99
DIA quant method	Mixed, LFQ split
DIA feature quant method	Sum
DIA top N fragments for quant	3
DIA top msms intensity quantile for quant	0.85
DIA min. msms intensity for quant	0
DIA precursor filter type	None
DIA min. fragment overlap score	1
DIA min. precursor score	0.5
DIA min. profile correlation	0
DIA global ML	☑
DIA adaptive mass accuracy	☐
DIA mass window factor	3.3
DIA background subtraction	☑
DIA background subtraction quantile	0.7
DIA background subtraction factor	4
DIA transfer q-value	0.3
DIA max. train instances	1000000
DIA LFQ ratio type	Median

DIA XGBoost Base Score	0.4
DIA XGBoost Sub Sample	0.9
DIA XGBoost learning objective	Binary logistic raw
DIA XGBoost Min child weight	9
DIA XGBoost Maximum Tree Depth	12
DIA XGBoost Estimators	580
DIA XGBoost Gamma	0.9
DIA XGBoost Max Delta Step	3
DIA no ML	☐
DIA only isos for recalibration	☑
DIA min. peaks for recalibration	5
DIA max. fragment charge	3
DIA use frag intensities for ML	
DIA use frag masses for ML	
DIA RT prediction	
DIA RT prediction second round	
DIA permute RT	
DIA permute CCS	

For database settings, we used the same databases downloaded from uniport as FragPipe and are shown in Supplementary Data 9. Other parameters include: Include contaminants *Yes*; Min. peptide length *7*; Max. peptide mass [Da] *4600*; Min. peptide length for unspecific search *8*; Max peptide length for unspecific search *25*; Variation mode *None*

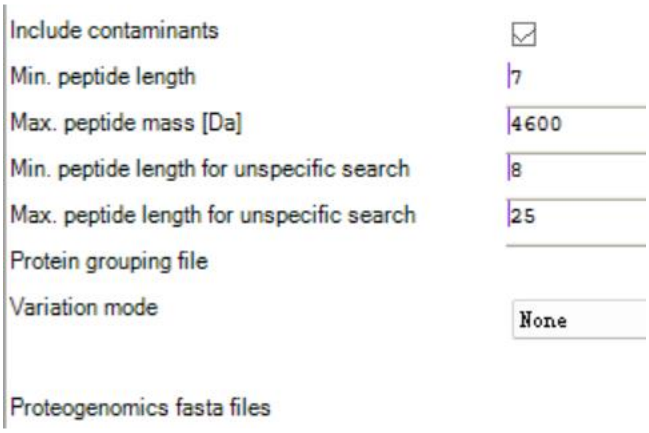

Protein quantification parameters: Label min. ratio count *2*; Peptides for quantification *Unique+razor*; Use only unmodified peptides and…*Yes* and *Oxidation (M)* *Acetyl (Protein N-term)*; Discard unmodified counterpart peptides *Yes*; Advanced ratio estimation *Yes*

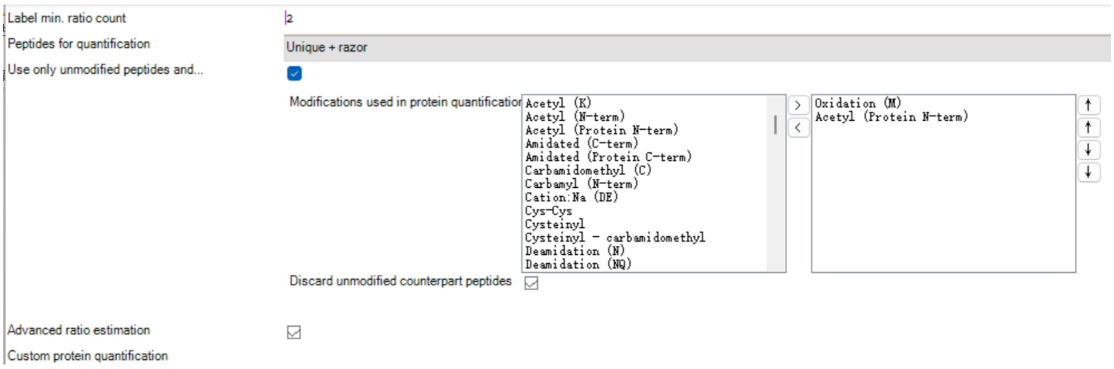

MS/MS analyzer parameters are shown as following screenshots:

FTMS MS/MS match tolerance unit	ppm
FTMS MS/MS de novo tolerance	25
FTMS MS/MS de novo tolerance unit	ppm
FTMS MS/MS deisotoping tolerance	7
FTMS MS/MS deisotoping tolerance unit	ppm
FTMS top peaks per Da interval	12
FTMS top x mass window [Da]	100
FTMS de-isotoping	☑
FTMS higher charges	☑
FTMS water loss	☑
FTMS water loss for cross links	☐
FTMS ammonia loss	☑
FTMS ammonia loss for cross links	☐
FTMS dependent losses	☑
FTMS recalibration	☐
ITMS MS/MS match tolerance	0.5
ITMS MS/MS match tolerance unit	Da
ITMS MS/MS de novo tolerance	0.5
ITMS MS/MS de novo tolerance unit	Da
ITMS MS/MS deisotoping tolerance	0.15
ITMS MS/MS deisotoping tolerance unit	Da

ITMS MS/MS match tolerance	0.5
ITMS MS/MS match tolerance unit	Da
ITMS MS/MS de novo tolerance	0.5
ITMS MS/MS de novo tolerance unit	Da
ITMS MS/MS deisotoping tolerance	0.15
ITMS MS/MS deisotoping tolerance unit	Da
ITMS top peaks per Da interval	8
ITMS top x mass window [Da]	100
ITMS de-isotoping	☐
ITMS higher charges	☑
ITMS water loss	☑
ITMS water loss for cross links	☐
ITMS ammonia loss	☑
ITMS ammonia loss for cross links	☐
ITMS dependent losses	☑
ITMS recalibration	☐

TOF MS/MS match tolerance	40
TOF MS/MS match tolerance unit	ppm
TOF MS/MS de novo tolerance	25
TOF MS/MS de novo tolerance unit	ppm
TOF MS/MS deisotoping tolerance	0.01
TOF MS/MS deisotoping tolerance unit	Da
TOF top peaks per Da interval	10
TOF top x mass window [Da]	100
TOF de-isotoping	☑
TOF higher charges	☑
TOF water loss	☑
TOF water loss for cross links	☐
TOF ammonia loss	☑
TOF ammonia loss for cross links	☐
TOF dependent losses	☑
TOF recalibration	☐

Unknown MS/MS match tolerance	20
Unknown MS/MS match tolerance unit	ppm
Unknown MS/MS de novo tolerance	25
Unknown MS/MS de novo tolerance unit	ppm
Unknown MS/MS deisotoping tolerance	7
Unknown MS/MS deisotoping tolerance unit	ppm
Unknown top peaks per Da interval	12
Unknown top x mass window [Da]	100
Unknown de-isotoping	☑
Unknown higher charges	☑
Unknown water loss	☑
Unknown water loss for cross links	☐
Unknown ammonia loss	☑
Unknown ammonia loss for cross links	☐
Unknown dependent losses	☑
Unknown recalibration	☐

Advance setting parameters including Decoy mode *Revert*; Use for occupancies *Normalized ratios*; Epsilon score for mutations *Yes*; Evaluate proteogenomic peptides separately *Yes*;

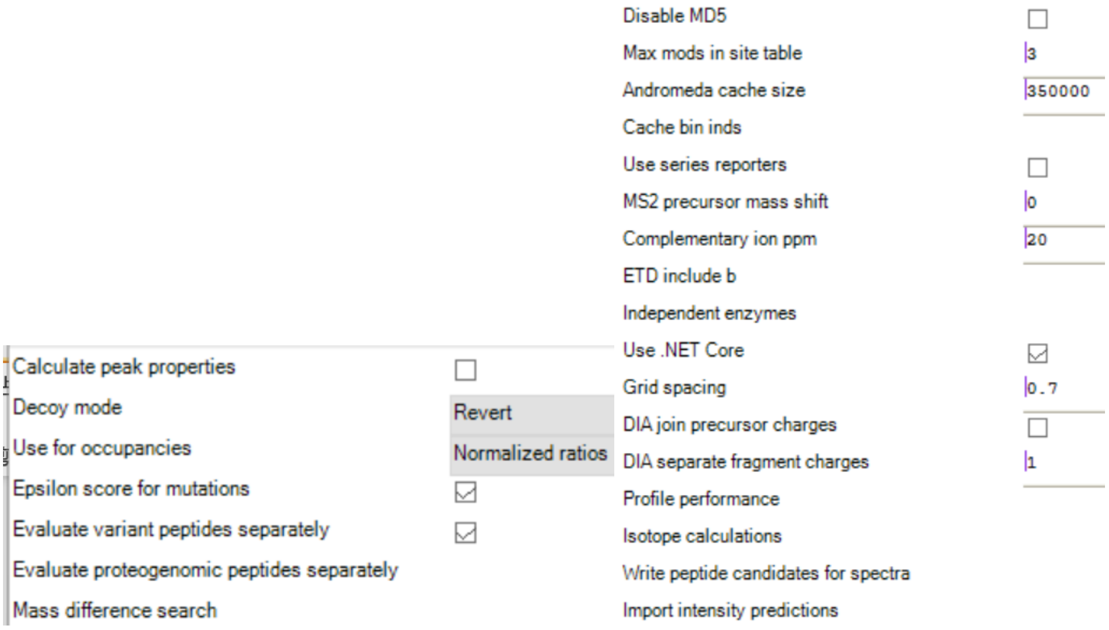

Identification parameters are shown in following screenshots and Match between runs is _enabled_:

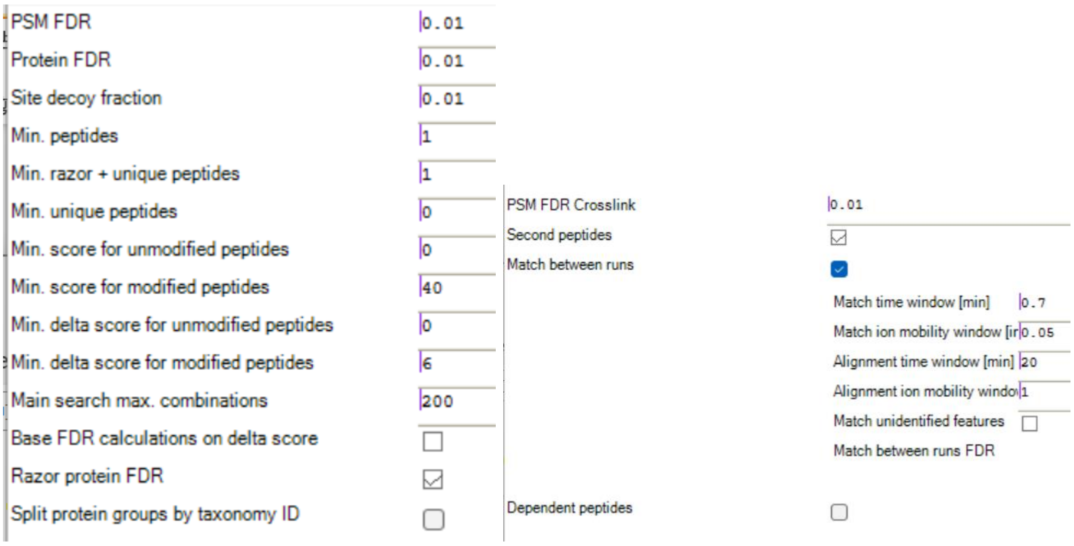

Label free quantification parameters are: TopN peptides _0_; Advanced site intensities _Yes_; Top3 _Yes_

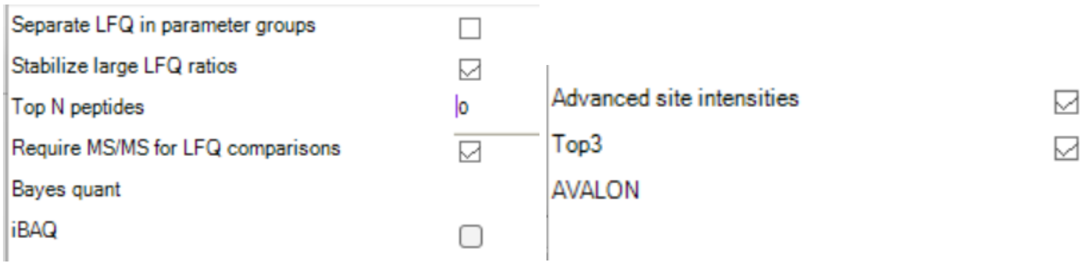

DIA-NN for quantifying DIA LFQ data

The DIA-NN 1.8.1 was used for DIA data quantification. We kept most of the parameters as default. Following screenshots show the detail parameter settings. We used the spectral library

generated by DIA-NN's deep learning spectra, RT and IM prediction tool.
Library free search is enabled.
The Protease is set to be Trypsin/P and at most 1 missed cleavage is permitted.
N-term excision is enabled, Cysteine carbamidomethylation is enabled as a fix modification.
The peptide length range is set as 7-30;
precursor charge range is set as 1-4;
Precursor m/z range is set as 300-1800;
Fragment ion m/z range is set as 200-1800;
Precursor FDR is set as 1%; Log level is set as 1;
MBR is enabled, heuristic protein inference is enabled;
Protein inference is set as "Genes"
Neural network class is set as "Single-pass mode";
Quantification strategy is set as "Robust LC(high precision)";
Cross-run normalization is set as "RT-dependent";
Library generation is set as "Smart profiling";
Speed and RAM usage is set as "Optimal results".

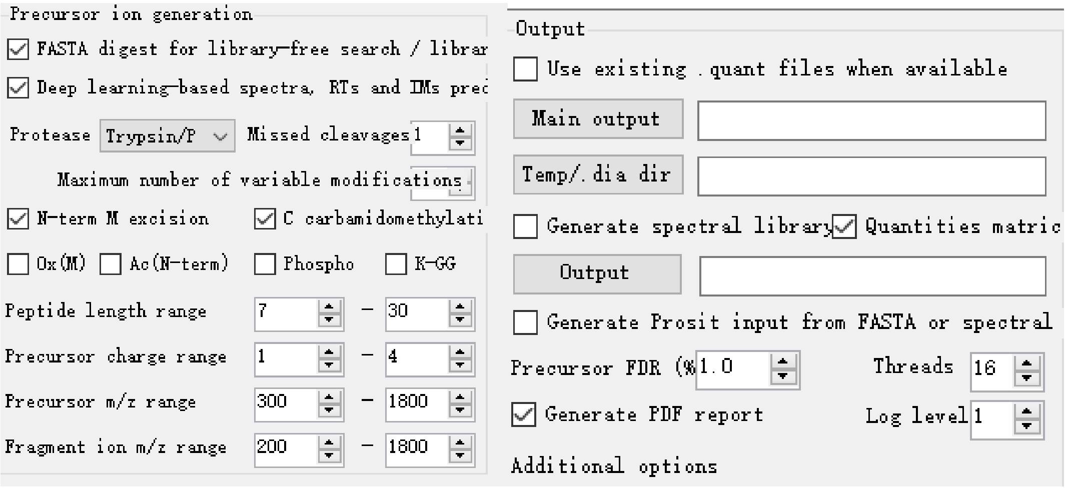

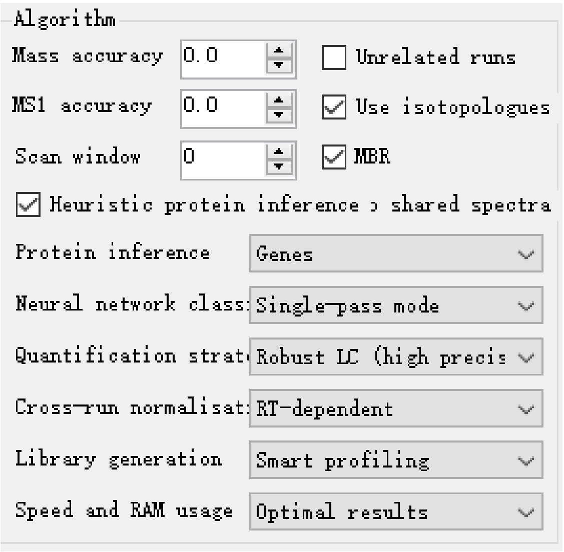

Spectronaut for quantifying DIA LFQ data

We used Spectronaut 18 as another quantification platform for DIA data analysis.

The BGS Factory Settings are as follows:

a) Peptides: Enzymes/Cleavage Rules *Trypsin/P*; Digest Type *Specific*; Max Peptide Length *52*; Min Peptide length *7*; Missed Cleavages *2*; Toggle N-terminal M *Yes*

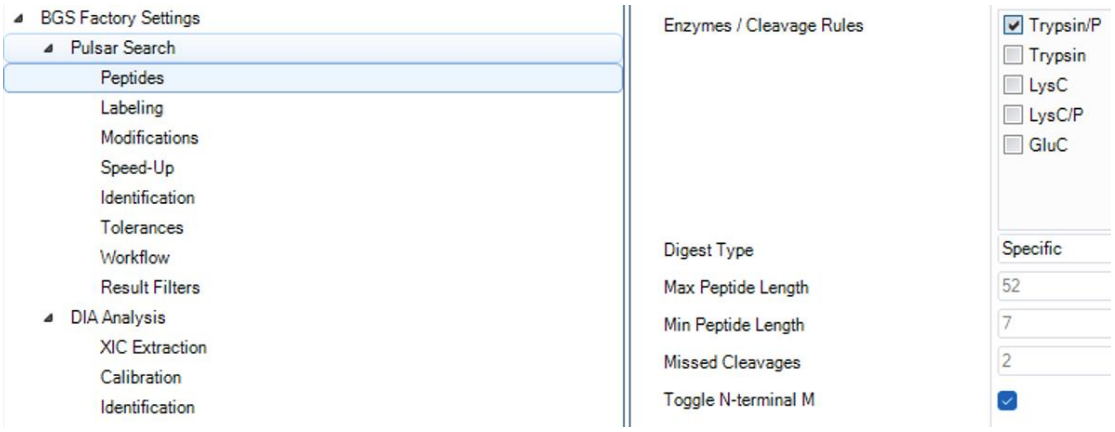

b) Modification: Max Variable Modifications *5*; Fixed Modifications *C*; Variable Modifications *Acetyl* and *M*

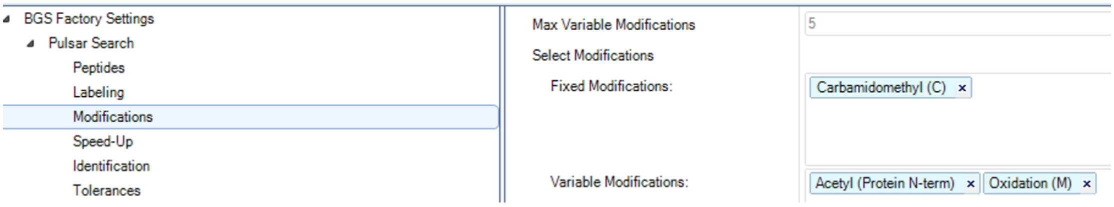

c) Speed-up: Use Dynamic IM Peak Filter *Yes*; Target TIC Fraction *0.9*; MS2 Index *Automatic*

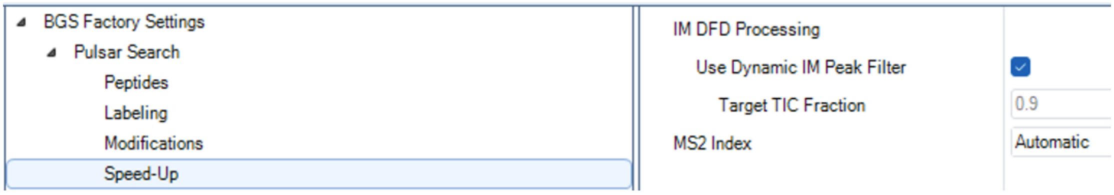

d) Identification: PSM FDR *0.01*; Peptide FDR *0.01*; Protein Group FDR *0.01*; directDIA Workflow *directDIA+(Deep)*

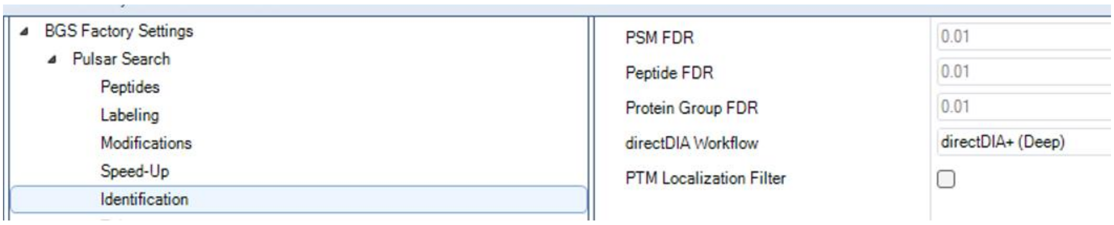

e) Tolerances: see below screenshot

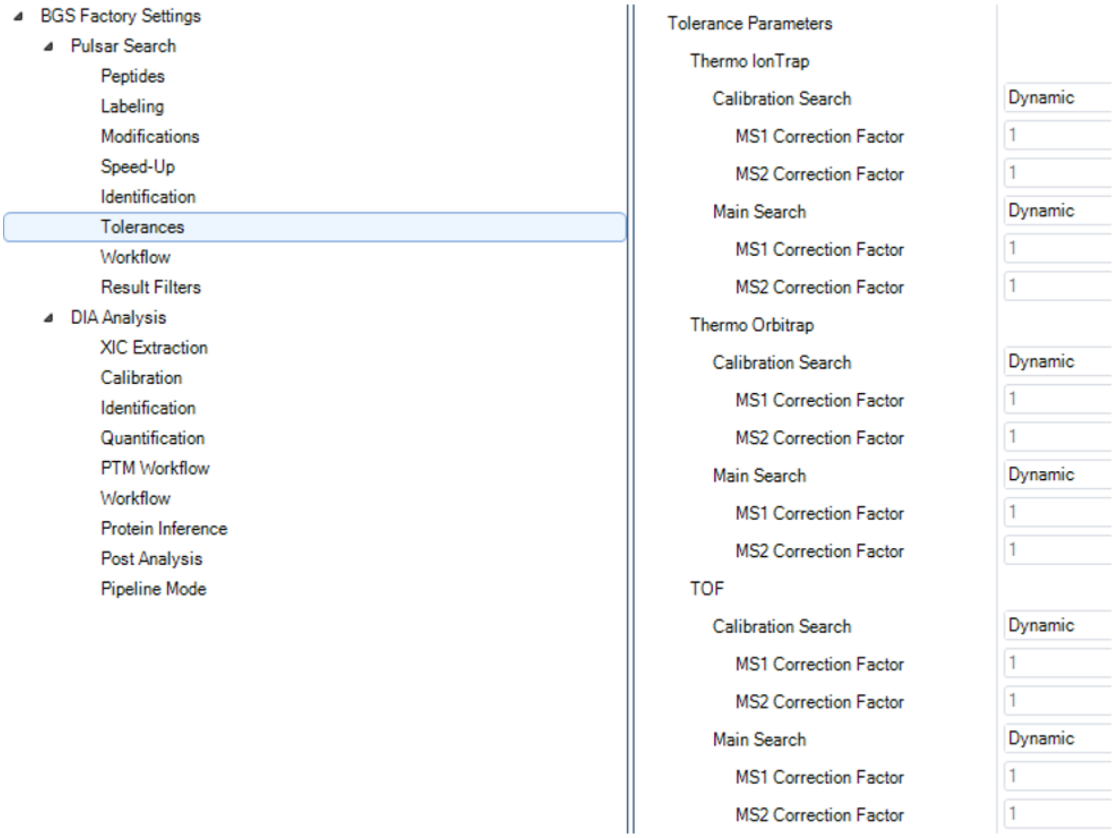

f) Workflow: Fragment Ion Selection Strategy *__Intensity Based__*; Use DNN predicted Ion Mobility *__Auto__*

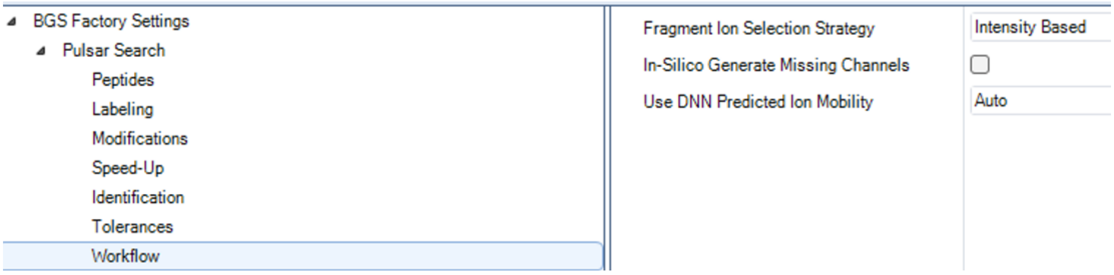

g) Result Filters: see below screenshot

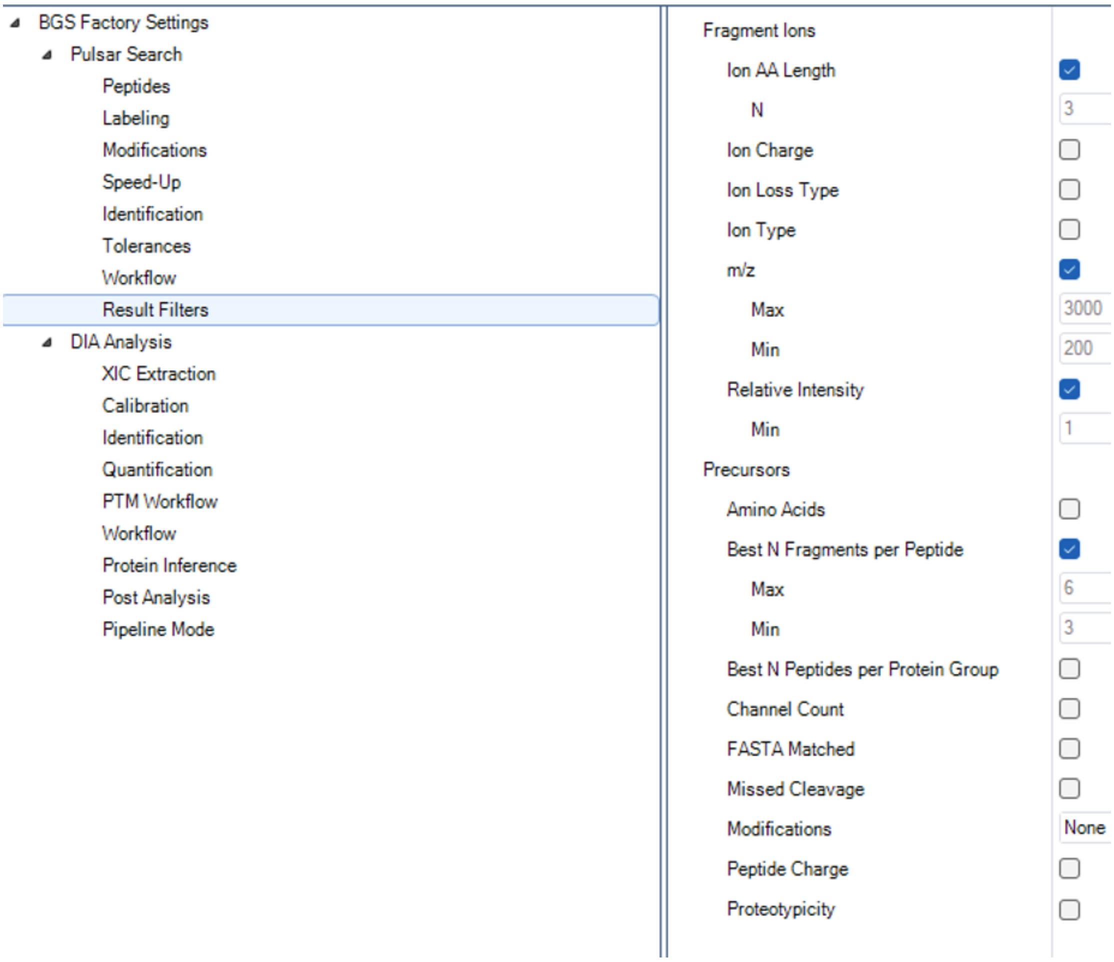

h) XIC Extraction:

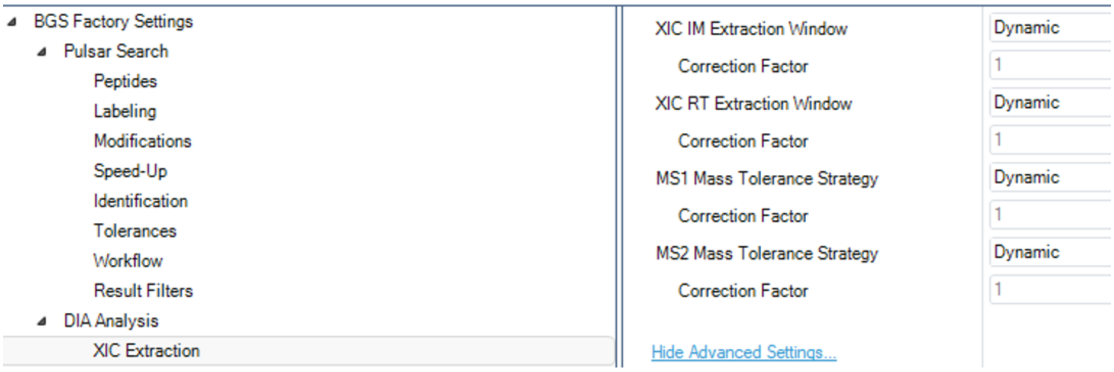

i) Calibration

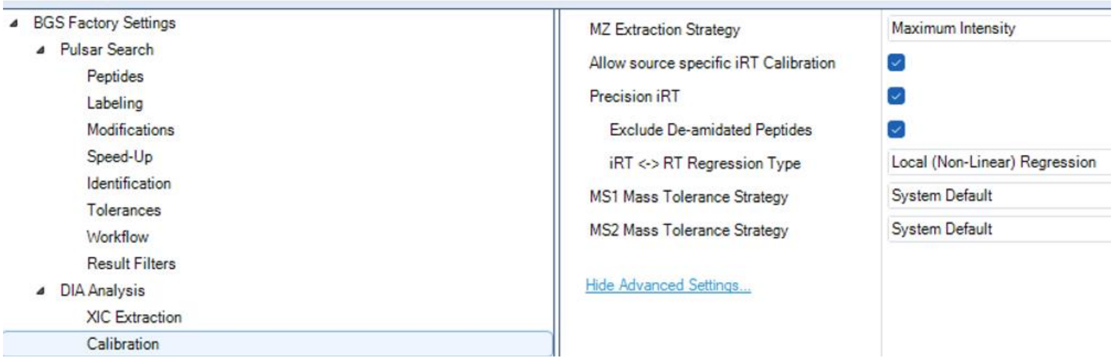

j) DIA analysis Identification:

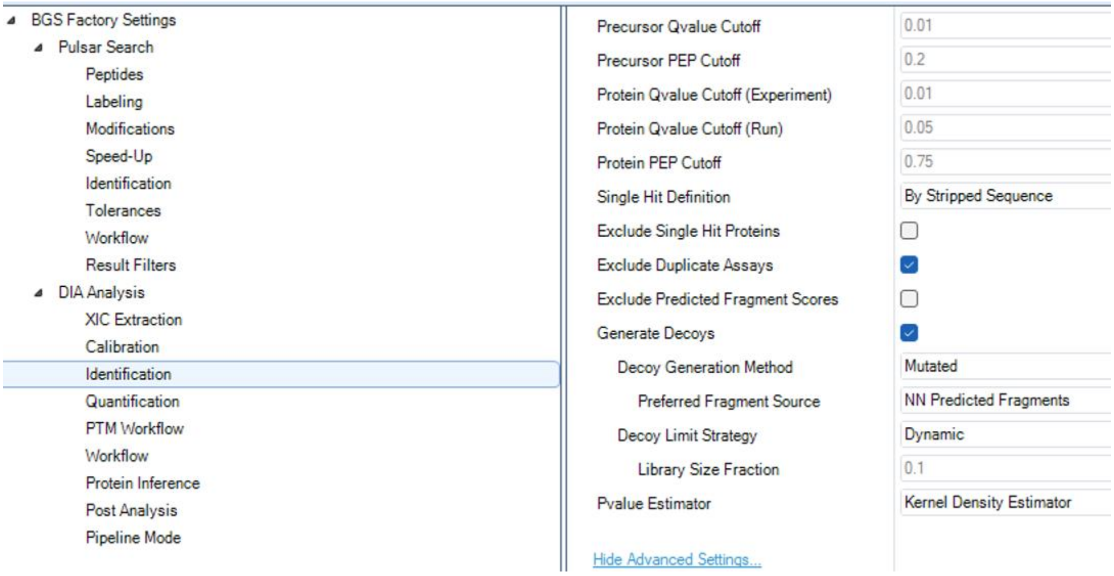

k) DIA analysis Quantification parameters:

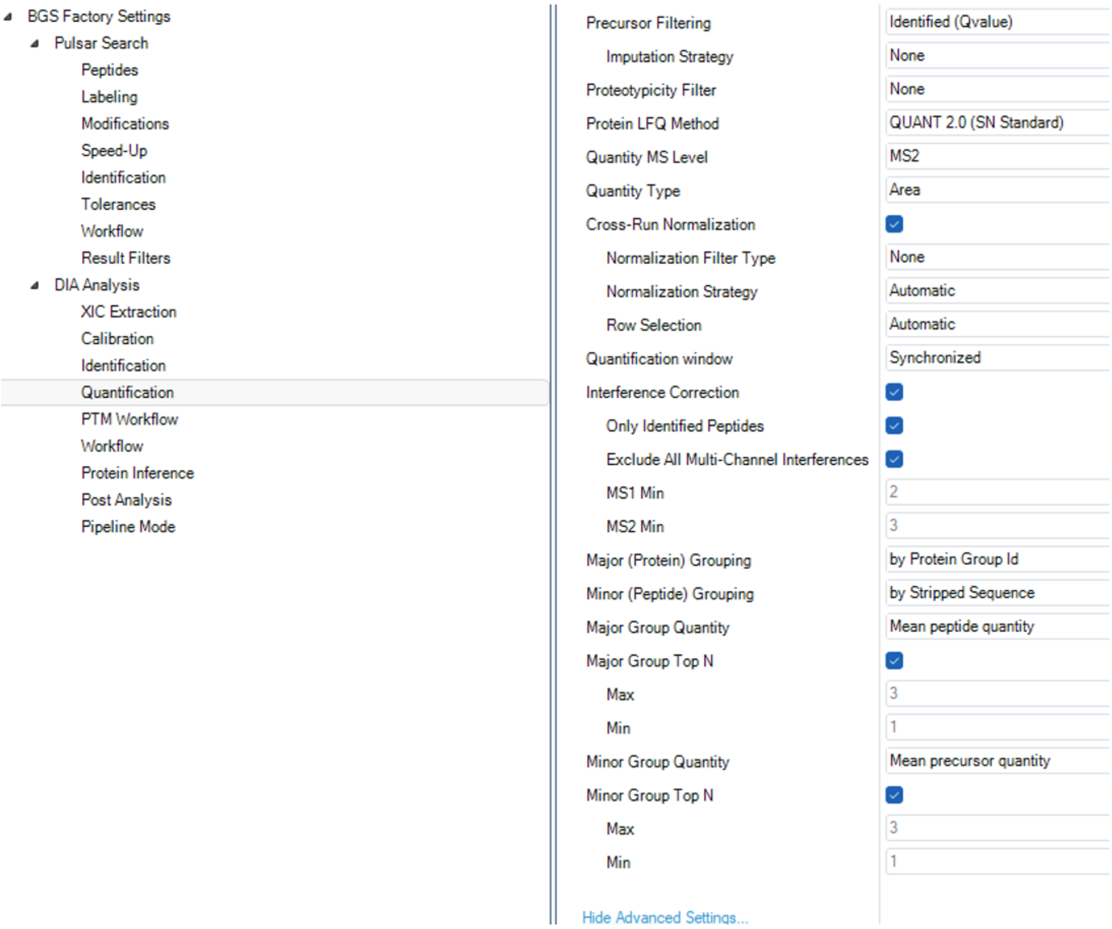

l) DIA analysis Workflow parameters:

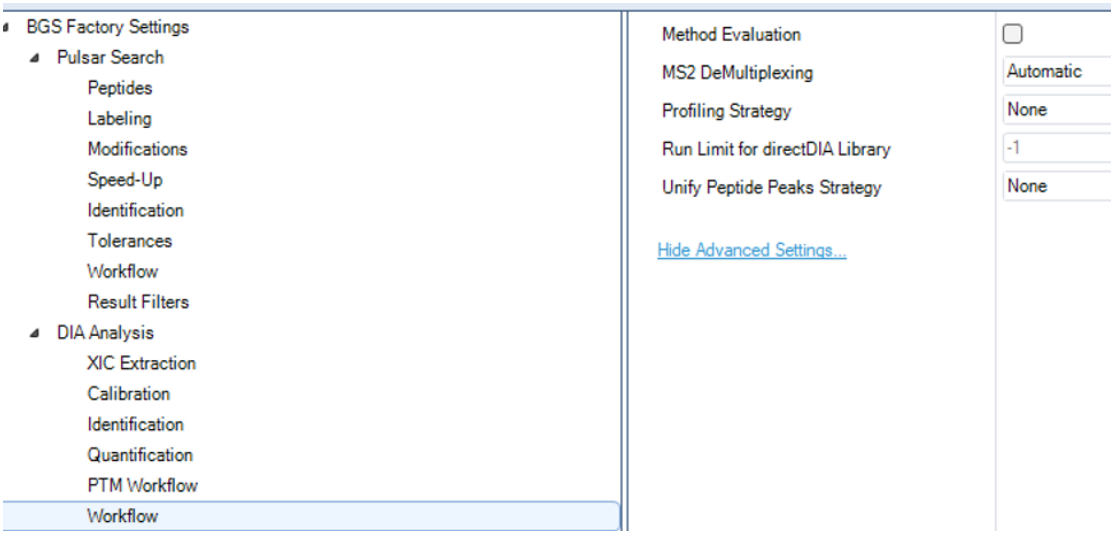

m) DIA analysis Protein inference parameters:

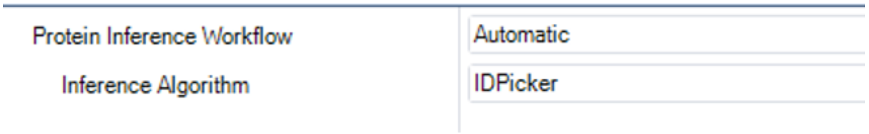

n) DIA analysis Post analysis parameters:

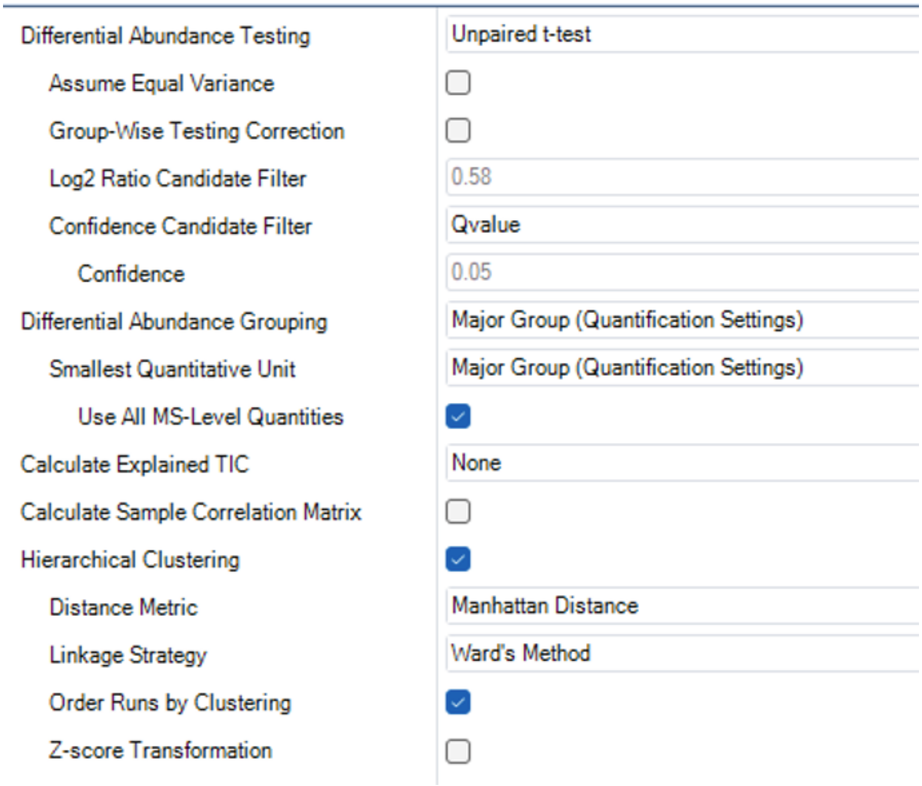

We note that to ensure the iq package can be worked with outputs from Spectronaut, the iq.rs file should be imported to Spectronaut, see https://cran.r-project.org/web/packages/iq/vignettes/iq-fast.html for more details.

FragPipe for quantifying TMT data

When FragPipe is used to quantify TMT data, the same software configuration is adopted as DDA LFQ quantification. The detail parameter settings are as follows:

When labeling peptide with TMT10 and quantification at MS2 level (dataset “HEqe277_TMT10”), the following parameters are used:

a.) Workflow sheet. Workflow “TMT10” is loaded.

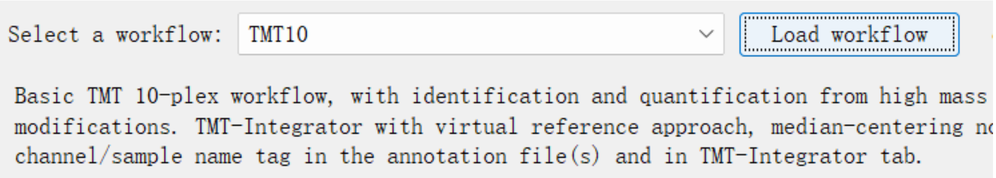

b.) Database sheet. (The same as FragPipe for DDA LFQ data quantification)

c.) MSFragger sheet. Peak Matching: Precursor mass tolerance *-20 PPM – 20 PPM*; Fragment mass tolerance *20PPM*; Calibration and Optimization *Mass calibration, parameter optimization*; Isotope error *-1/0/1/2/3*

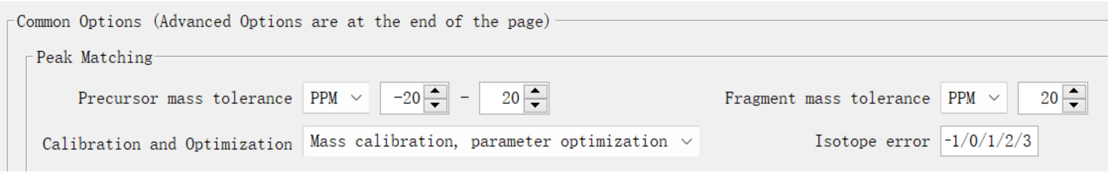

Protein Digestion: Cleavage *ENZYMATIC*; Clip N-term M *True*; Enzyme name 1 *stricttrypsin*; Load rules *stricttrypsin*; Cuts *KR*; Missed cleavages 1 *2*; Sense 1 *C*; Peptide length *7-50*; peptide mass range *500-5000*; split database *1*

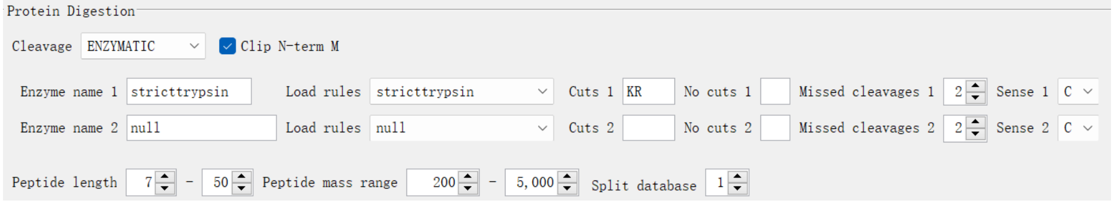

Modifications: Max variable mods on a peptide *3*; Max combinations *5000*; site enabled *M*, *[^, n^, S*; all of the fixed modifications are selected.

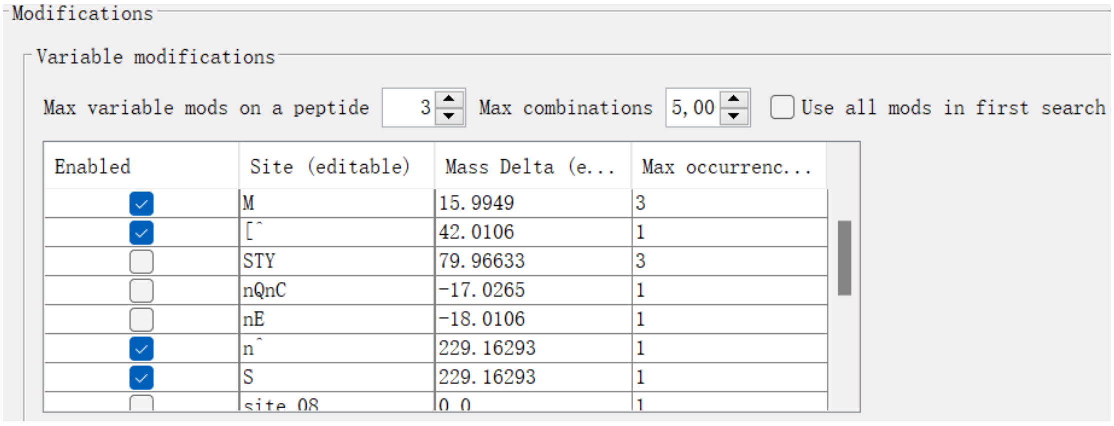

Spectral Processing: Activation Type Filter *all*; Min Peaks *15*; Clear m/z range *125.5 – 131.5*; Use top N peaks *150*; Min ratio *0.01*; Remove precursor peak *Only peak with precursor charge*; removal m/z range *-1.5 – 1.5*

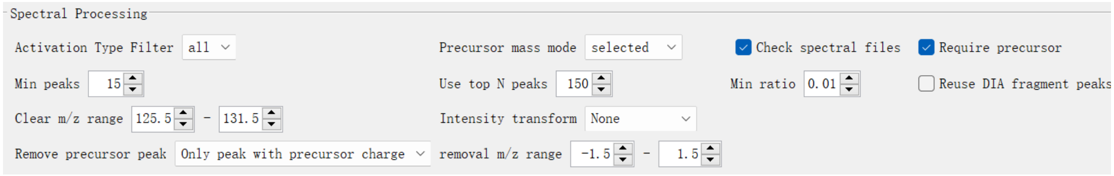

d.) Validation sheet

Run Validation Tools *Yes*; Run MSBooster *Yes*; Predict RT *Yes*; Predict spectra *Yes*

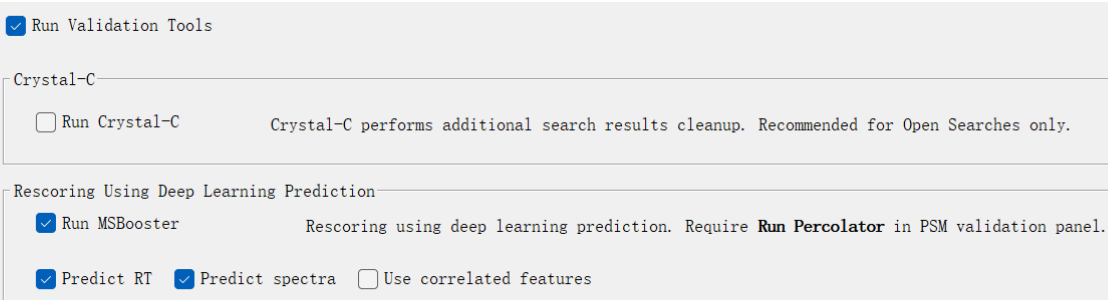

Run PSM validation *Yes*; Run Percolator *Yes*; Min probability *0.5*; Cmd line opts *--only-psms --no-terminate --post-processing-tdc*

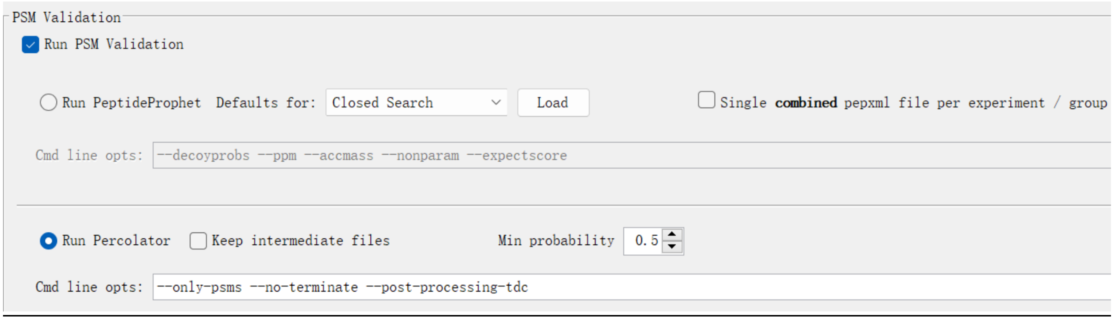

Protein Inference: Run ProteinProphet *Yes*; Cmd line opts *--maxppmdiff 2000000 –minprob 0.5*; Generate reports *Yes*; Filter *–sequential --picked --prot 0.01*; Generate MSstats files *Yes*; Generate peptide-level summary *Yes*; Generate protein-level summary *Yes*

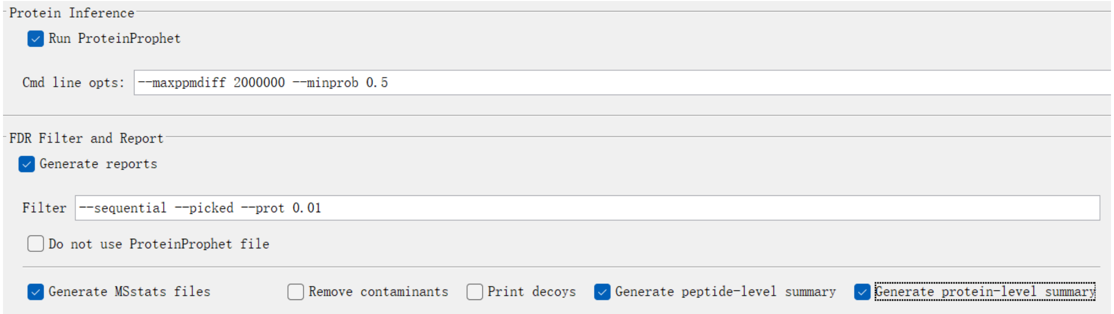

e.) Quant (Isobaric) sheet

Run TMT-Integrator *Yes*

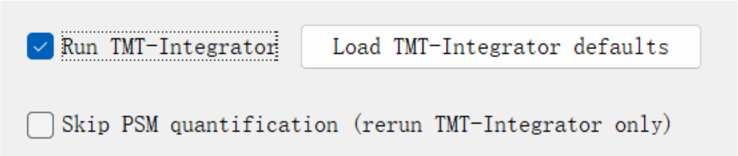

Basic Options Label type *TMT-10*; Quant level *2*; Mass tolerance (ppm) *20*; Define reference *Virtual;* Group by *All*; Normalization *MD (median centering)*; Mod tag *none*; Min site probability *-1*; Glycan FDR filter *-1*

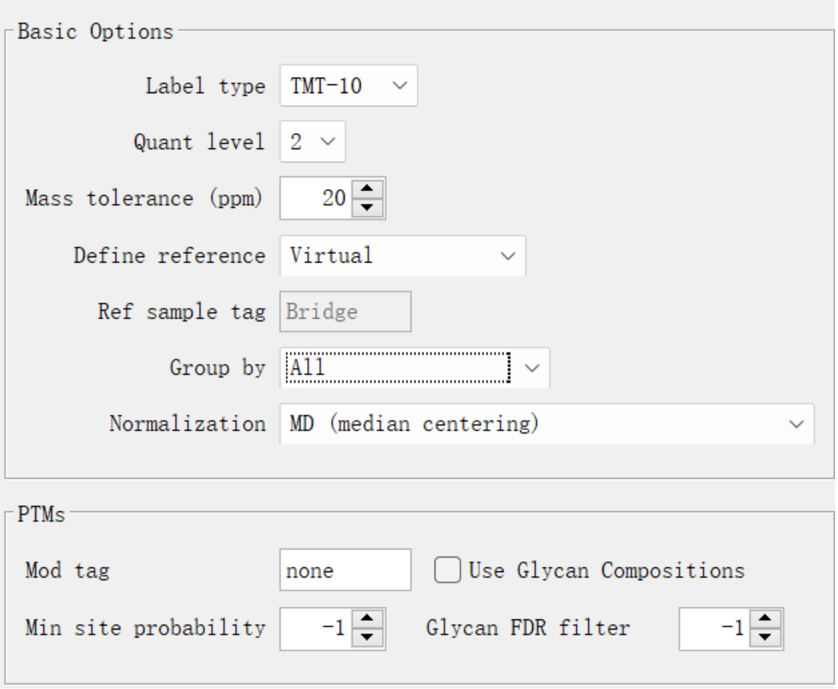

Advanced Options Peptide-Gene uniqueness *Keep all PSMs*; Peptide-Protein uniqueness *Unique+Razor*; Min PSM probability *0.9*; Min purity *0.5*; Min Intensity (percent) *0.05*; Min best peptide probability *0.9*; Min NTT *0*; Aggregation method *Median*; Best PSM *Yes*; Allow overlabel *Yes*; Outlier removal *Yes*; Exclude proteins *none*; Use MS1 intensity *Yes*; Top 3 ions *Yes*; Log2 transform the intensity *Yes*

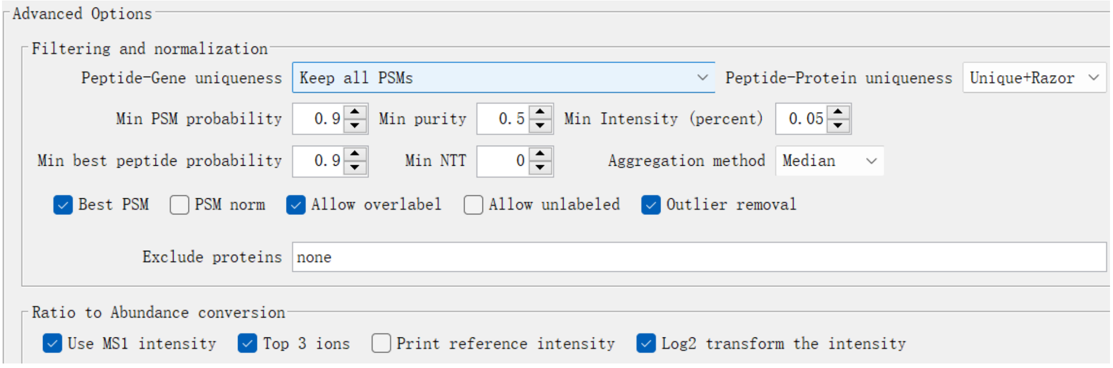

For TMT11 labeling with MS3 quantification (dataset "HYqfl683_TMT11"):

a.) Workflow sheet. Workflow "TMT10-MS3" is loaded.

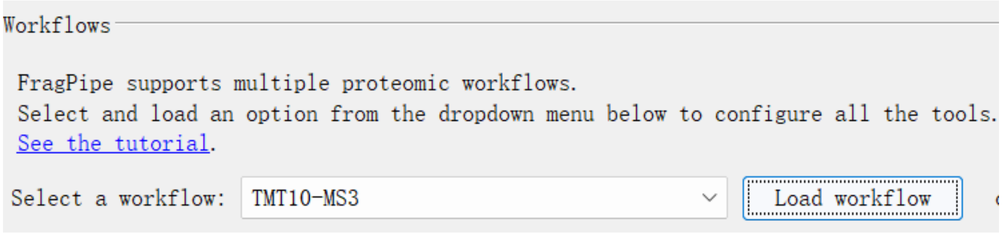

b.) Database sheet. (The same as FragPipe for DDA LFQ data quantification)

c.) MSFragger sheet. Peak Matching: Precursor mass tolerance *-20 PPM – 20 PPM*; Fragment mass tolerance *0.6* Da; Calibration and Optimization *Mass calibration, parameter optimization*; Isotope error *0/1/2/3*

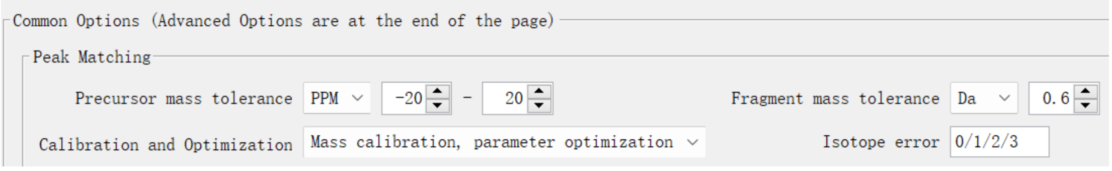

Protein Digestion: Cleavage *ENZYMATIC*; Clip N-term M *True*; Enzyme name 1 *stricttrypsin*; Load rules *stricttrypsin*; Cuts *KR*; Missed cleavages 1 *2*; Sense 1 *C*; Peptide length *7-50*; peptide mass range *500-5000*; split database *1*

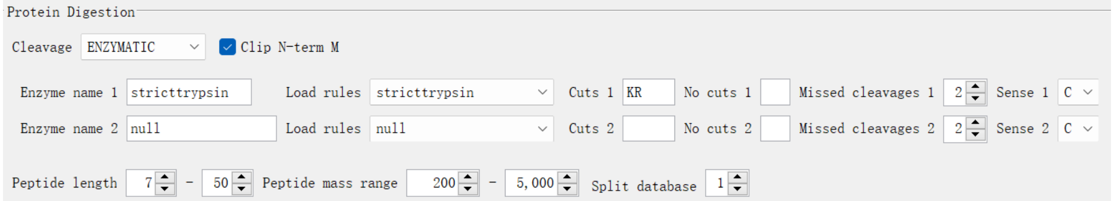

Modifications: Max variable mods on a peptide *3*; Max combinations *5000*; site enabled *M*, *[^, n^*; all of the fixed modifications are selected.

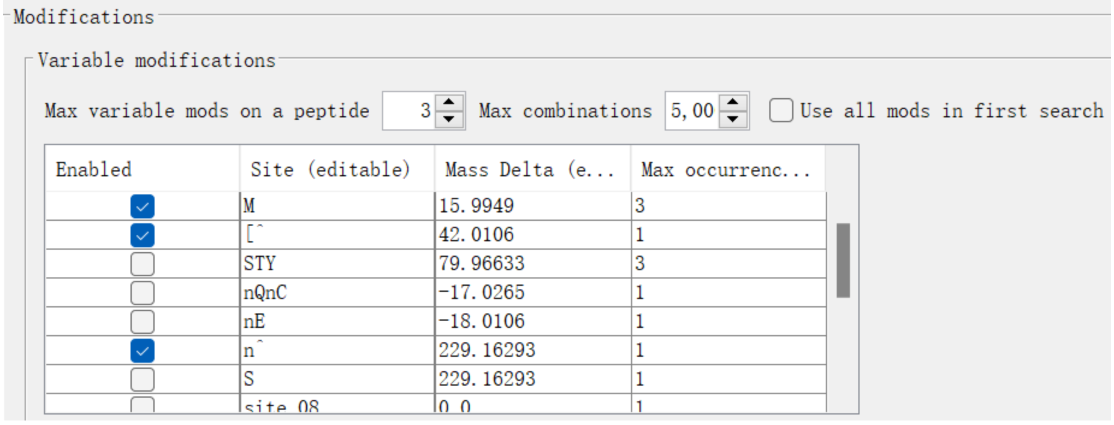

Spectral Processing: Activation Type Filter *all*; Precursor mass mode *selected*; Check spectral files *Yes*; Require precursor *Yes*; Min Peaks *15*; Clear m/z range *125.5 – 131.5*; Use top N peaks *150*; Min ratio *0.01*; Intensity transform *None*; Remove precursor peak *Only peak with precursor charge*; removal m/z range *-1.5 – 1.5*

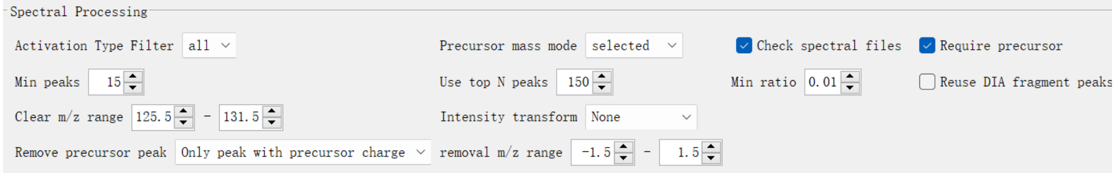

d.) Validation sheet

Run Validation Tools *Yes*; Run MSBooster *Yes*; Predict RT *Yes*; Predict spectra *Yes*

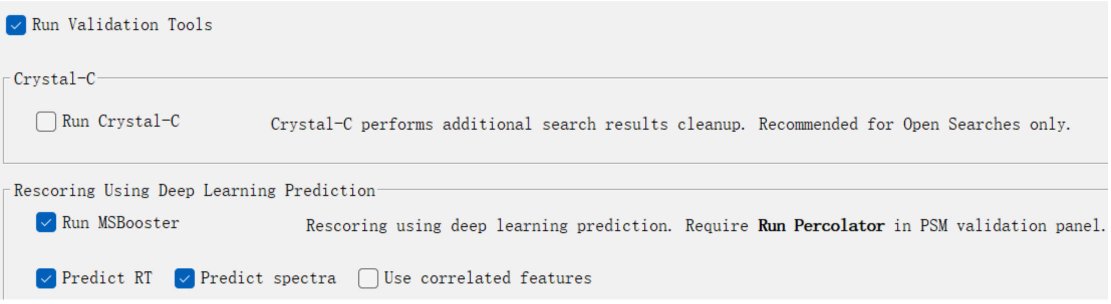

Run PSM validation *Yes*; Run Percolator *Yes*; Min probability *0.5*; Cmd line opts *--only-psms --no-terminate --post-processing-tdc*

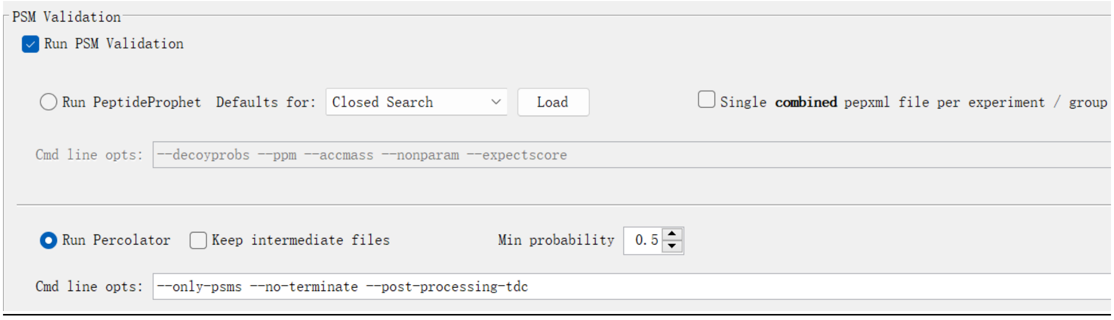

Protein Inference: Run ProteinProphet *Yes*; Cmd line opts *--maxppmdiff 2000000 –minprob 0.5*; Generate reports *Yes*; Filter *–sequential --picked --prot 0.01*; Generate MSstats files *Yes*; Generate peptide-level summary *Yes*; Generate protein-level summary *Yes*

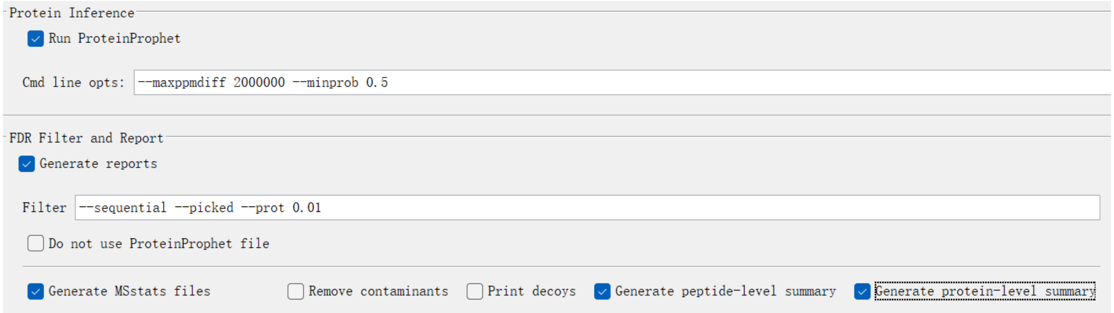

e.) Quant (Isobaric) sheet

Run TMT-Integrator *Yes*

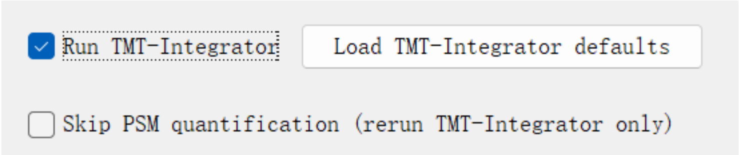

Basic Options Label type *TMT-11*; Quant level *3*; Mass tolerance (ppm) *20*; Define reference *Virtual;* Group by *All*; Normalization *MD (median centering)*; Mod tag *none*; Min site probability *-1*; Glycan FDR filter *-1*

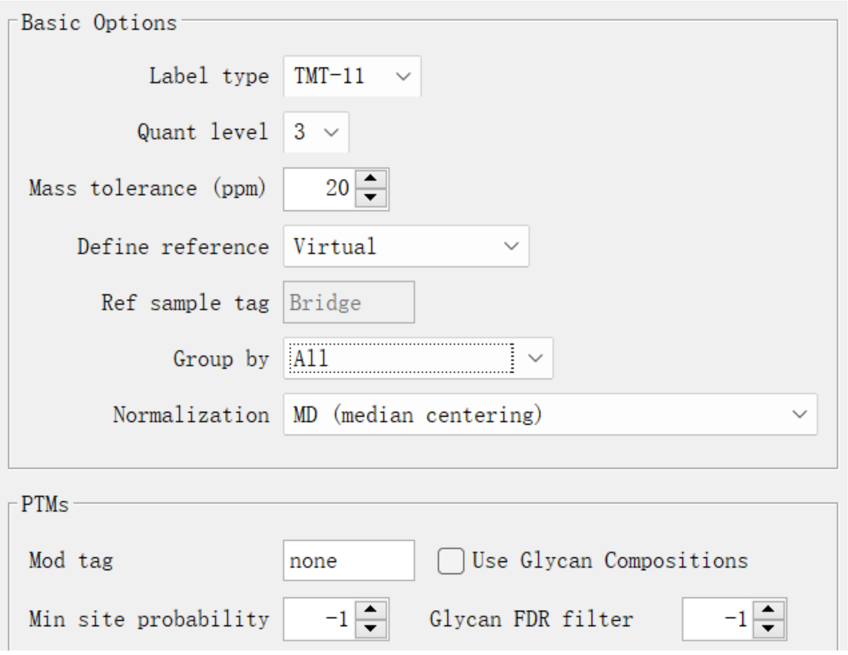

Advanced Options Peptide-Gene uniqueness *Keep all PSMs*; Peptide-Protein uniqueness *Unique+Razor*; Min PSM probability *0.9*; Min purity *0.5*; Min Intensity (percent) *0.05*; Min best peptide probability *0.9*; Min NTT *0*; Aggregation method *Median*; Best PSM *Yes*; Allow overlabel *Yes*; Outlier removal *Yes*; Exclude proteins *none*; Use MS1 intensity *Yes*; Top 3 ions *Yes*; Log2 transform the intensity *Yes*

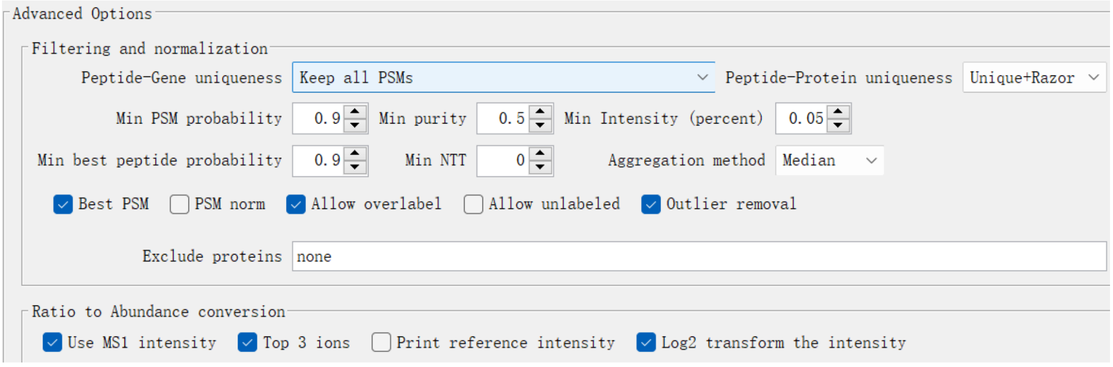

For TMT16 labeling with MS2 quantification (datasets “HYms2faims815_TMT16” and “HYms2815_TMT16”):

a.) Workflow sheet. Workflow “TMT16” is loaded.

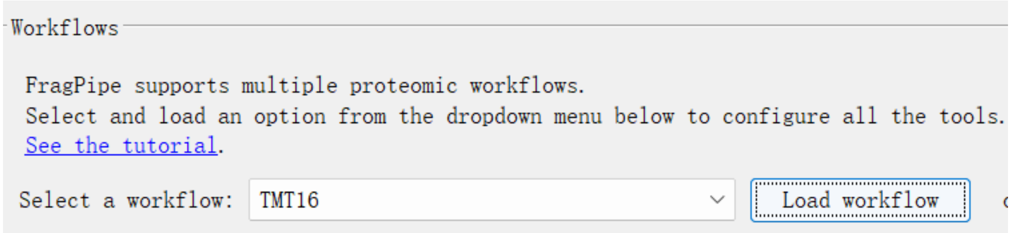

b.) Database sheet. (The same as FragPipe for DDA LFQ data quantification)

c.) MSFragger sheet. Peak Matching: Precursor mass tolerance *-20 PPM – 20 PPM*; Fragment mass tolerance *20* PPM; Calibration and Optimization *Mass calibration,*

parameter optimization; Isotope error *-1/0/1/2/3*

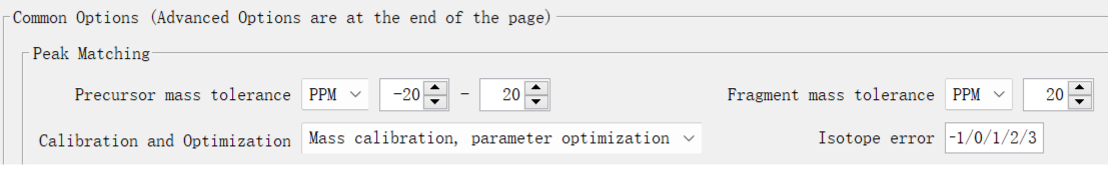

Protein Digestion: Cleavage *ENZYMATIC*; Clip N-term M *True*; Enzyme name 1 *stricttrypsin*; Load rules *stricttrypsin*; Cuts *KR*; Missed cleavages 1 *2*; Sense 1 *C*; Peptide length *7-50*; peptide mass range *500-5000*; split database *1*

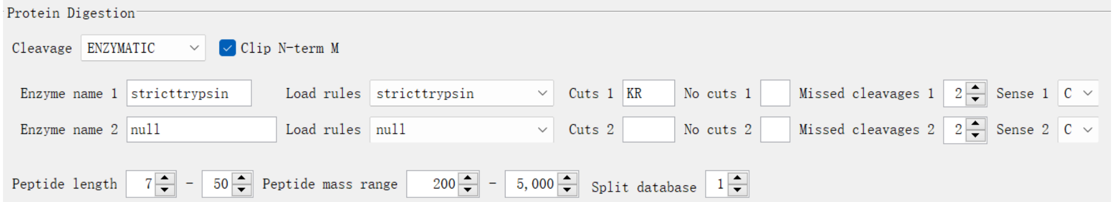

Modifications: Max variable mods on a peptide *3*; Max combinations *5000*; site enabled *M*, *[^, n^*; all of the fixed modifications are selected.

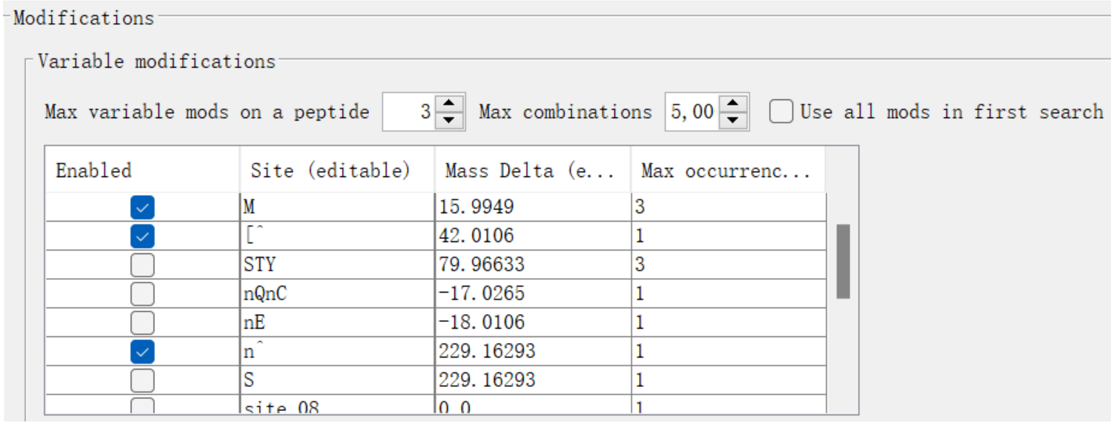

Spectral Processing: Activation Type Filter *all*; Precursor mass mode *selected*; Check spectral files *Yes*; Require precursor *Yes*; Min Peaks *15*; Clear m/z range *125.5 – 134.5*; Use top N peaks *150*; Min ratio *0.01*; Intensity transform *None*; Remove precursor peak *Only peak with precursor charge*; removal m/z range *-1.5 – 1.5*

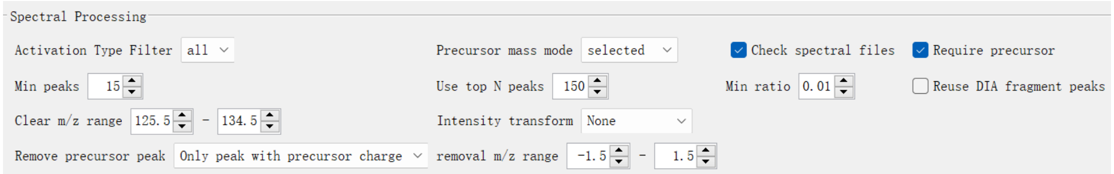

d.) Validation sheet

Run Validation Tools *Yes*

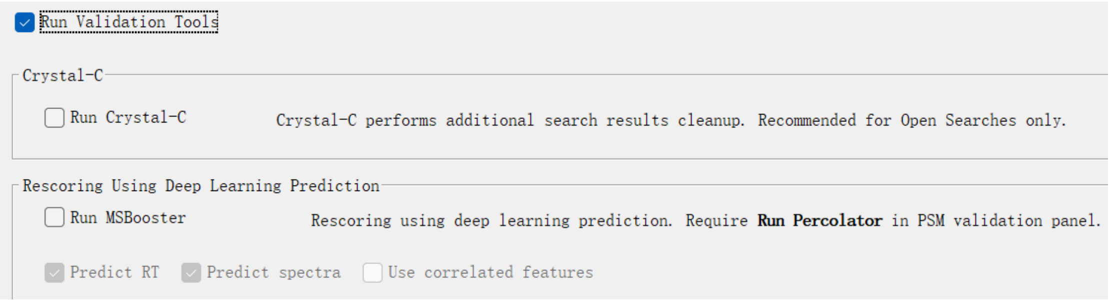

Run PSM validation *Yes*; Run Percolator *Yes*; Min probability *0.5*; Cmd line opts *--only-psms --no-terminate --post-processing-tdc*

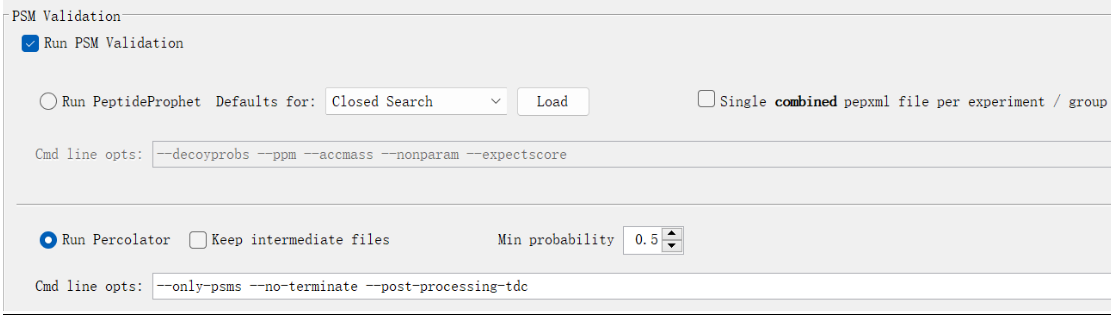

Protein Inference: Run ProteinProphet *Yes*; Cmd line opts *--maxppmdiff 2000000 –minprob 0.5*; Generate reports *Yes*; Filter *–sequential --picked --prot 0.01*; Generate MSstats files *Yes*; Generate peptide-level summary *Yes*; Generate protein-level summary *Yes*

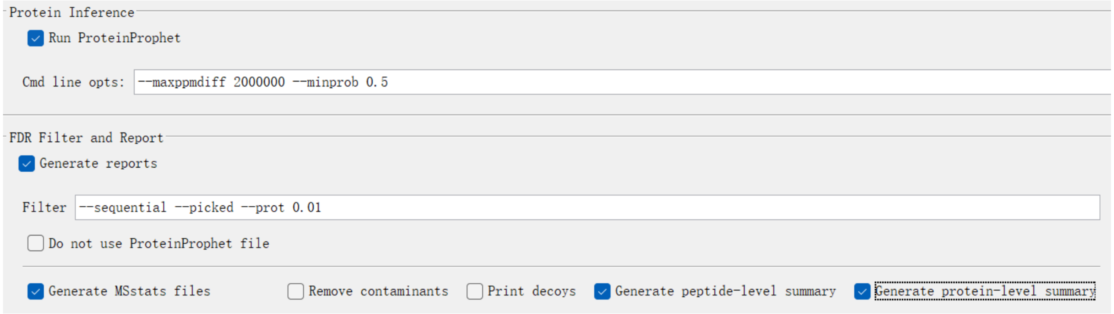

e.) Quant (Isobaric) sheet

Run TMT-Integrator *Yes*

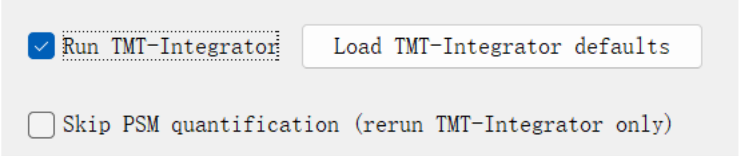

Basic Options Label type *TMT-16*; Quant level *2*; Mass tolerance (ppm) *20*; Define reference *Virtual;* Group by *All*; Normalization *MD (median centering)*; Mod tag *none*; Min site probability *-1*; Glycan FDR filter *-1*

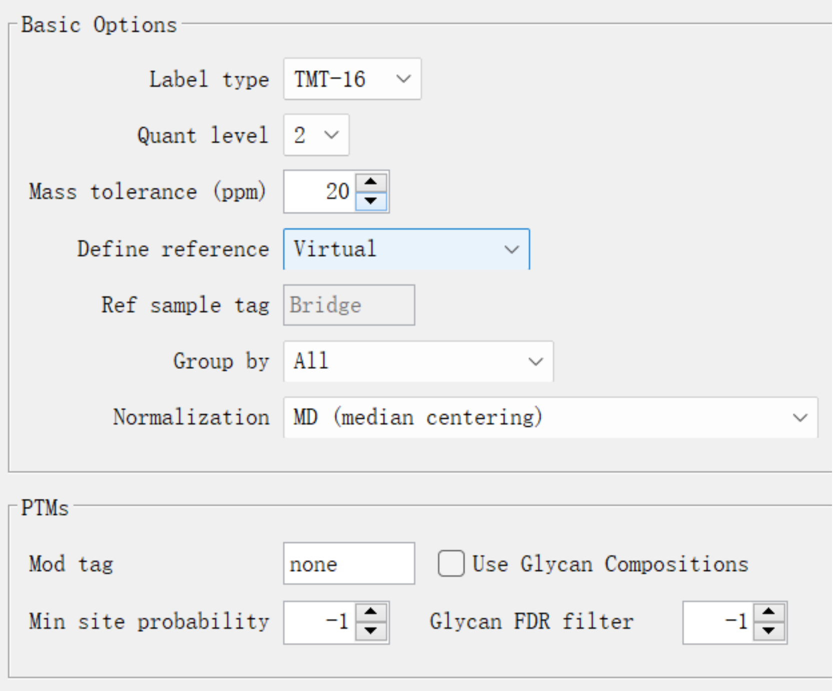

Advanced Options Peptide-Gene uniqueness *Keep all PSMs*; Peptide-Protein uniqueness *Unique+Razor*; Min PSM probability *0.9*; Min purity *0.5*; Min Intensity (percent) *0.05*; Min best peptide probability *0.9*; Min NTT *0*; Aggregation method *Median*; Best PSM *Yes*; Allow overlabel *Yes*; Outlier removal *Yes*; Exclude proteins *none*; Use MS1 intensity *Yes*; Top 3 ions *Yes*; Log2 transform the intensity *Yes*

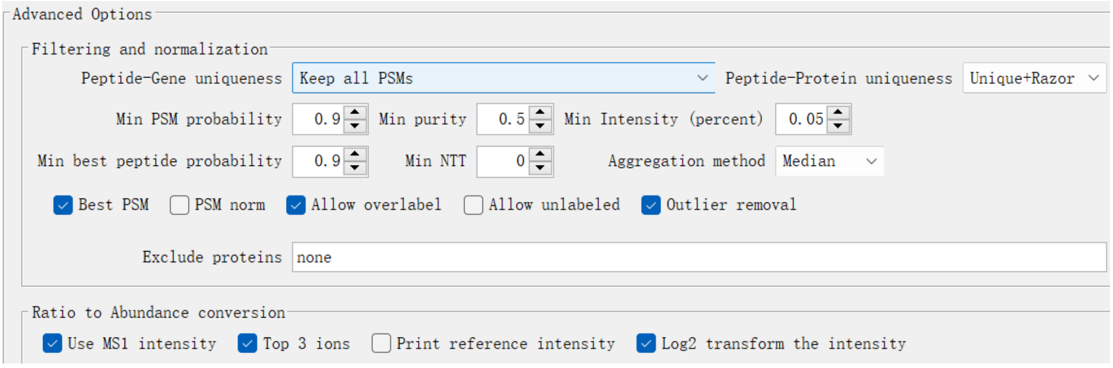

For TMT16 labeling with MS3 quantification (datasets "HYsps2815_TMT16"):

a.) Workflow sheet. Workflow "TMT16-MS3" is loaded.

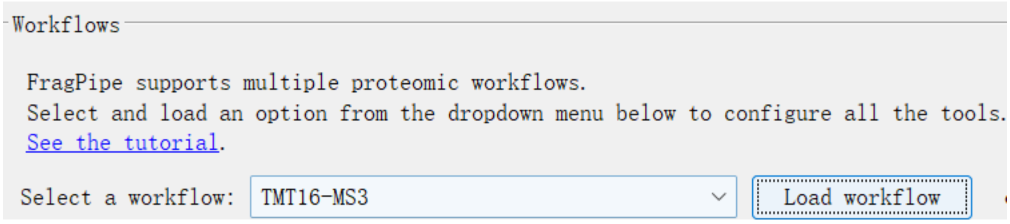

b.) Database sheet. (The same as FragPipe for DDA LFQ data quantification)

c.) MSFragger sheet. Peak Matching: Precursor mass tolerance *-20 PPM – 20 PPM*; Fragment mass tolerance *0.6* Da; Calibration and Optimization *Mass calibration,*

parameter optimization; Isotope error *0/1/2/3*

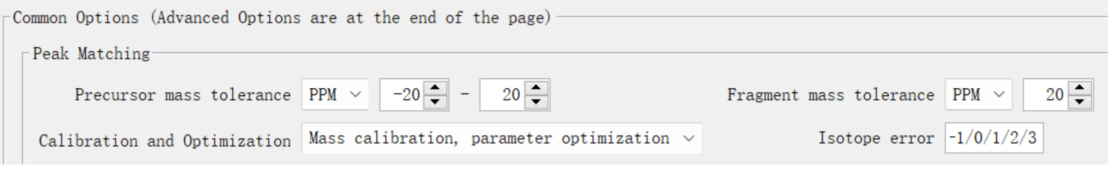

Protein Digestion: Cleavage *ENZYMATIC*; Clip N-term M *True*; Enzyme name 1 *stricttrypsin*; Load rules *stricttrypsin*; Cuts *KR*; Missed cleavages 1 *2*; Sense 1 *C*; Peptide length *7-50*; peptide mass range *500-5000*; split database *1*

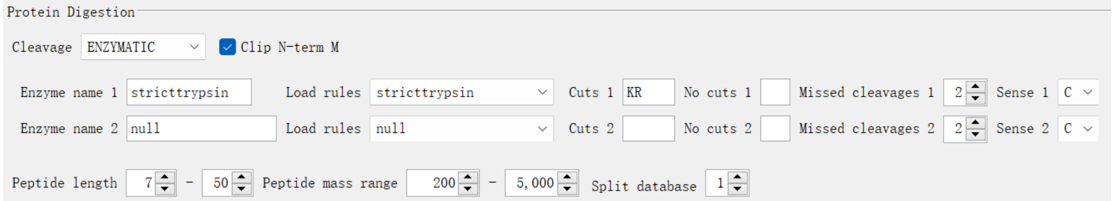

Modifications: Max variable mods on a peptide *3*; Max combinations *5000*; site enabled *M*, *[^, n^*; all of the fixed modifications are selected.

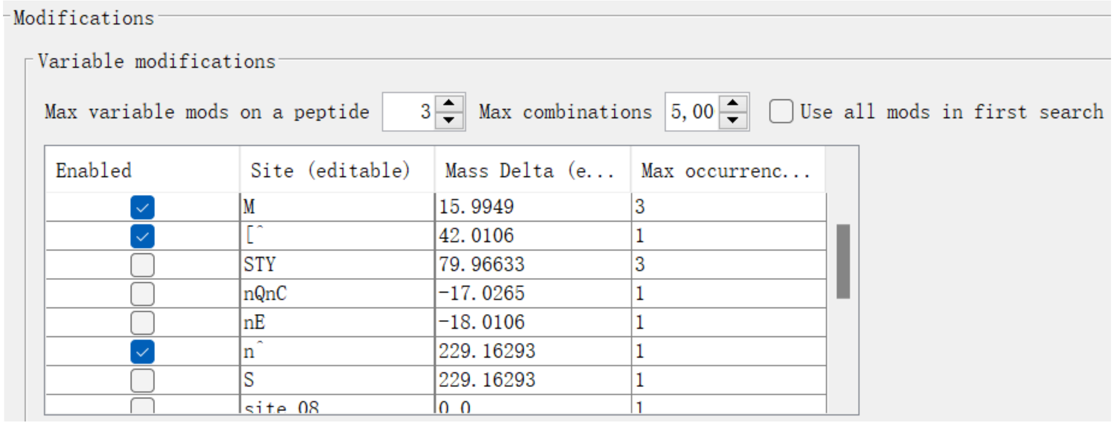

Spectral Processing: Activation Type Filter *all*; Precursor mass mode *selected*; Check spectral files *Yes*; Require precursor *Yes*; Min Peaks *15*; Clear m/z range *125.5 – 134.5*; Use top N peaks *150*; Min ratio *0.01*; Intensity transform *None*; Remove precursor peak *Only peak with precursor charge*; removal m/z range *-1.5 – 1.5*

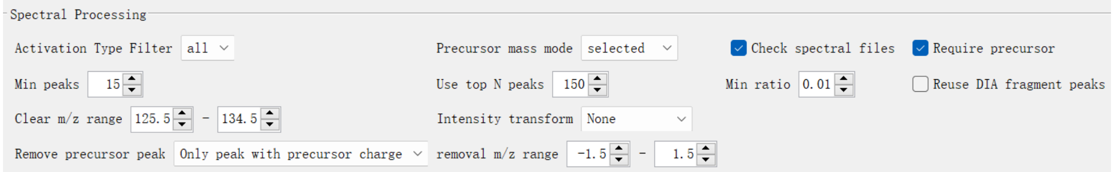

d.) Validation sheet

Run Validation Tools *Yes*

e.)

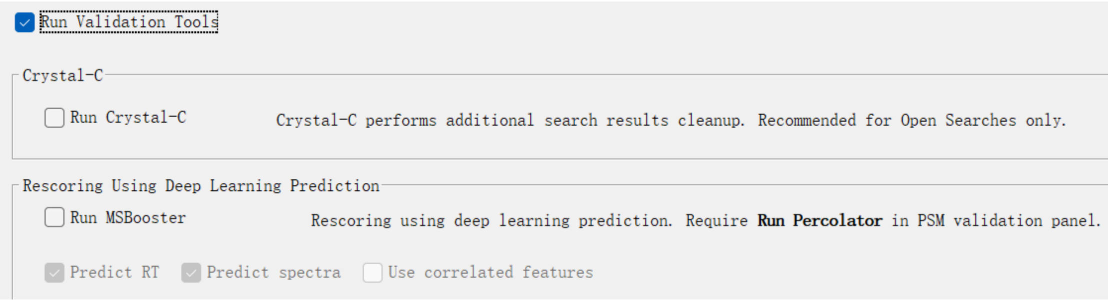

Run PSM validation *Yes*; Run Percolator *Yes*; Min probability *0.5*; Cmd line opts *--only-psms --no-terminate --post-processing-tdc*

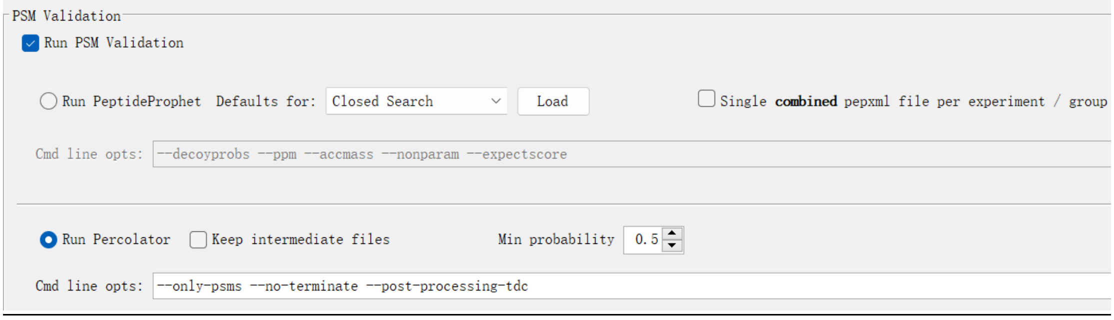

Protein Inference: Run ProteinProphet *Yes*; Cmd line opts *--maxppmdiff 2000000 –minprob 0.5*; Generate reports *Yes*; Filter *–sequential --picked --prot 0.01*; Generate MSstats files *Yes*; Generate peptide-level summary *Yes*; Generate protein-level summary *Yes*

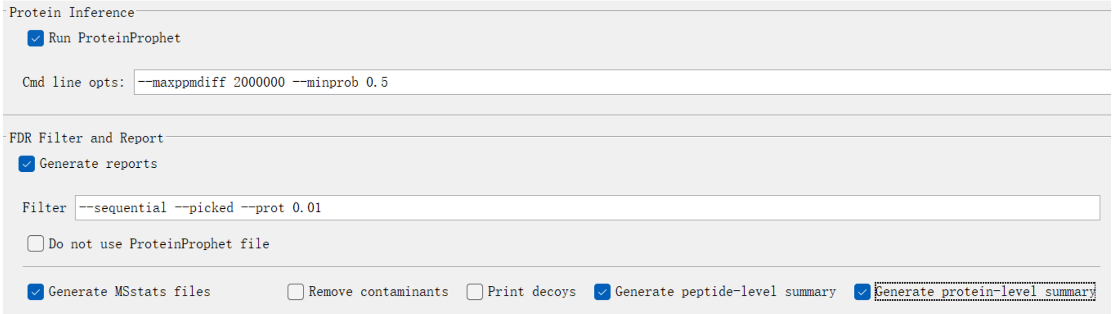

f.) Quant (Isobaric) sheet

Run TMT-Integrator *Yes*

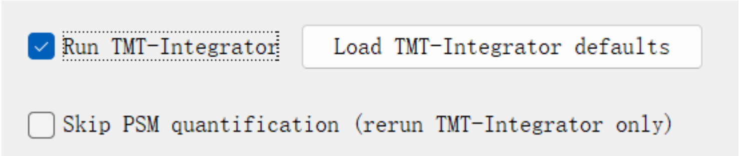

Basic Options Label type *TMT-16*; Quant level *3*; Mass tolerance (ppm) *20*; Define reference *Virtual;* Group by *All*; Normalization *MD (median centering)*; Mod tag *none*; Min site probability *-1*; Glycan FDR filter *-1*

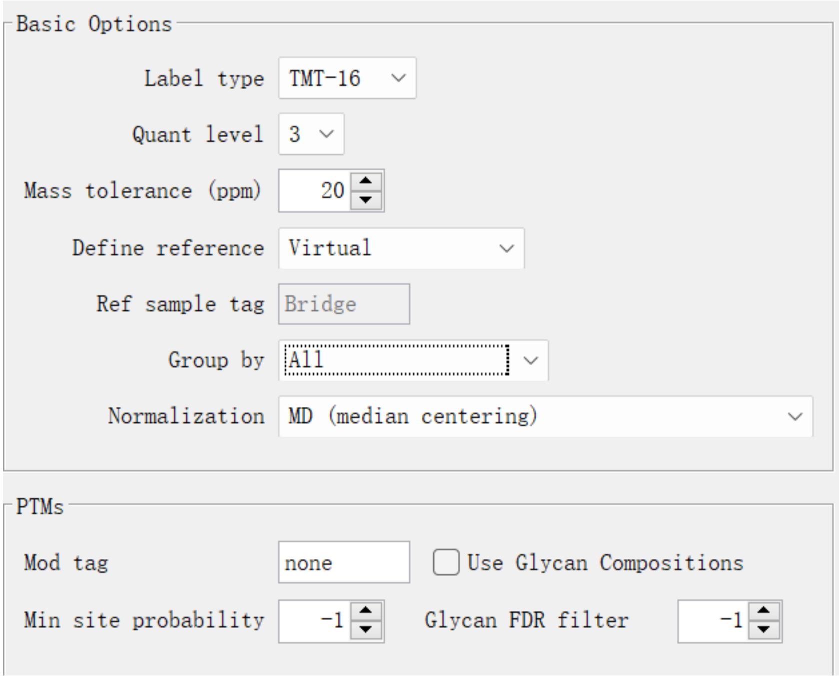

Advanced Options Peptide-Gene uniqueness <u>*Keep all PSMs*</u>; Peptide-Protein uniqueness <u>*Unique+Razor*</u>; Min PSM probability <u>*0.9*</u>; Min purity <u>*0.5*</u>; Min Intensity (percent) <u>*0.05*</u>; Min best peptide probability <u>*0.9*</u>; Min NTT <u>*0*</u>; Aggregation method <u>*Median*</u>; Best PSM <u>*Yes*</u>; Allow overlabel <u>*Yes*</u>; Outlier removal <u>*Yes*</u>; Exclude proteins <u>*none*</u>; Use MS1 intensity <u>*Yes*</u>; Top 3 ions <u>*Yes*</u>; Log2 transform the intensity <u>*Yes*</u>

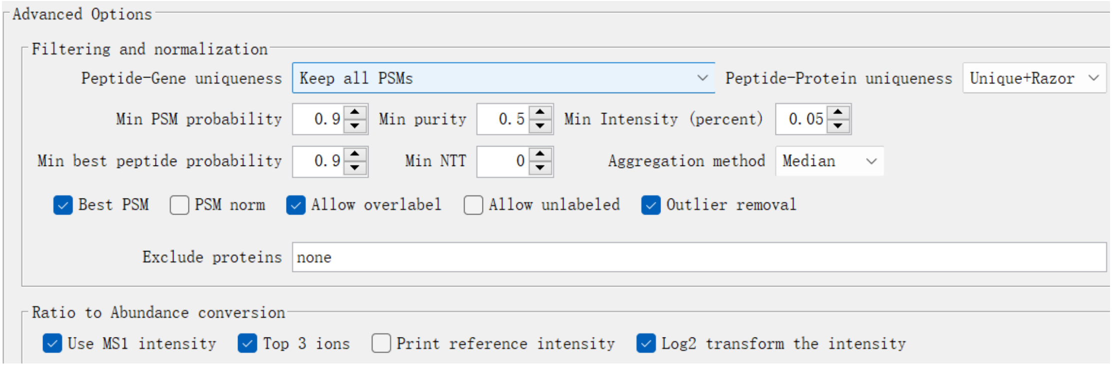

Maxquant for quantifying TMT data

The software Maxquant was also used to quantify TMT data. **We used Maxquant 2.4.4.0 to quantify the TMT data instead as we get some errors when using Maxquant 2.1.0.0**. The parameter settings are shown as below:

For TMT labeling with MS2 level quantification without FAIMS (HEqe277_TMT10, HYms2815_TMT16), the parameter "Type" is set as "<u>*Reporter ion MS2*</u>". Then for TMT10 (HEqe277_TMT10) and TMT16 (HYms2815_TMT16), we click "<u>*10plex TMT*</u>" and "<u>*16plex*</u>" respectively. The Reporter mass tol. [Da] is set as <u>*0.003*</u> as default. Mode is set as <u>*Direct*</u>; Normalization is set as <u>*"None"*</u>.

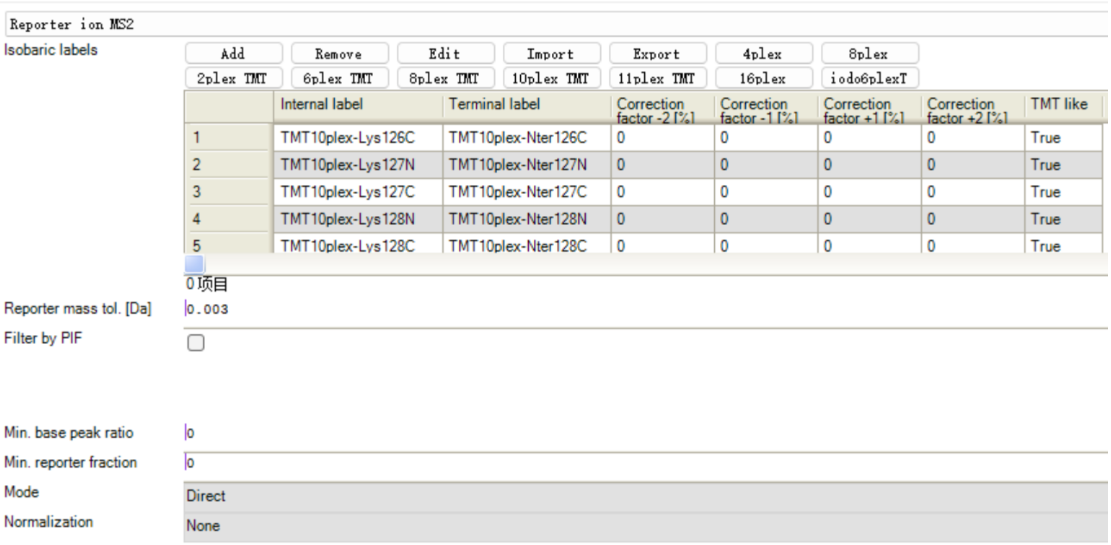

For TMT labeling with MS3 level quantification and without FAIMS (HYqfl683_TMT11 and HYsps2815_TMT16). The parameter "Type" is set as *"Reporter ion MS3"*. Then for TMT11 (HYqfl683_TMT11) and TMT16 (HYsps2815_TMT16), we click "*11plex TMT*" and "*16plex*" respectively. The Reporter mass tol. [Da] is set as *0.003* as default; Normalization is set as *"None"*.

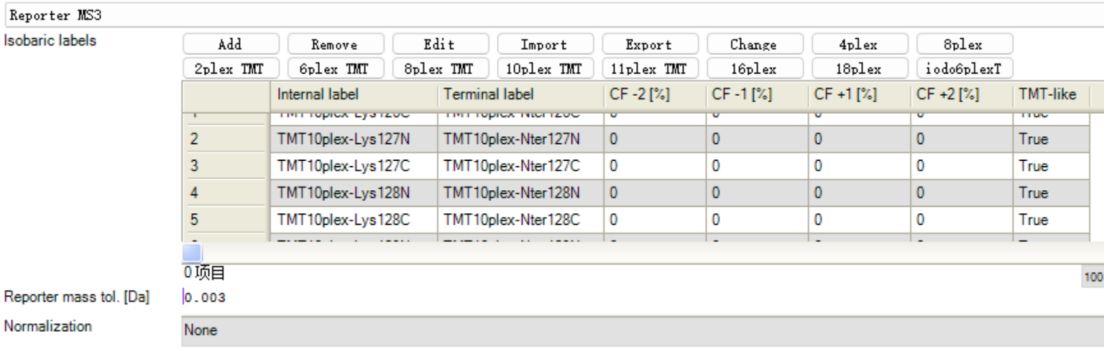

For TMT labeling with MS2 level quantification and with FAIMS (HYms2faims815_TMT16), The parameter "Type" is set as "*FAIMS-Reporter ion MS2*". Then, we click "*16plex*". The Reporter mass tol. [Da] is set as *0.003* as default; Normalization is set as *"None"*. Min. base peak ratio is set as *0*; Min. reporter fraction is set as *0*; Mode is set as *Direct*.

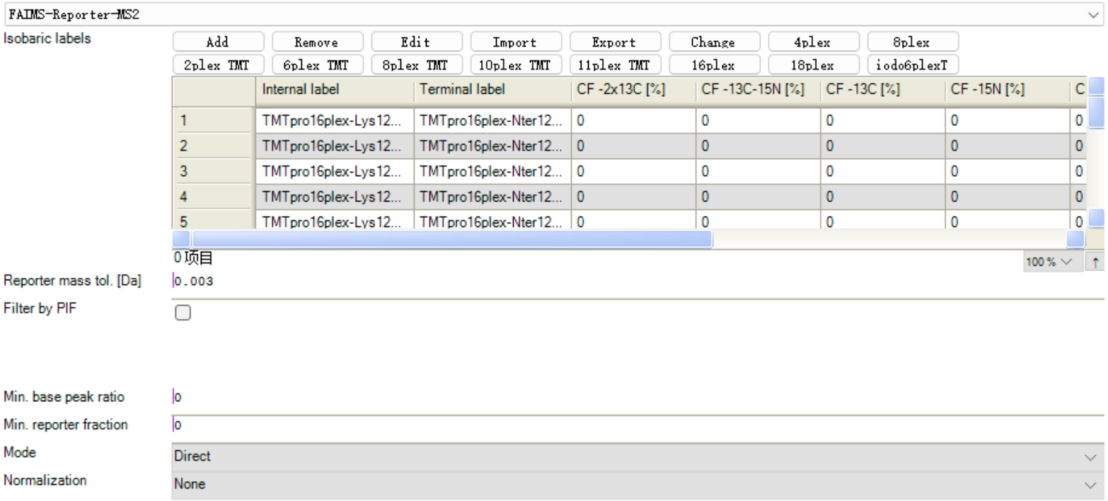

The remain parameters are set as shown in following screenshots:

Modifications: Variable modifications *Oxidation (M)* *Acetyl (Protein N-term)*; Fixed modifications *Carbamidomethyl (C)*; Max. number of modifications per peptide 5

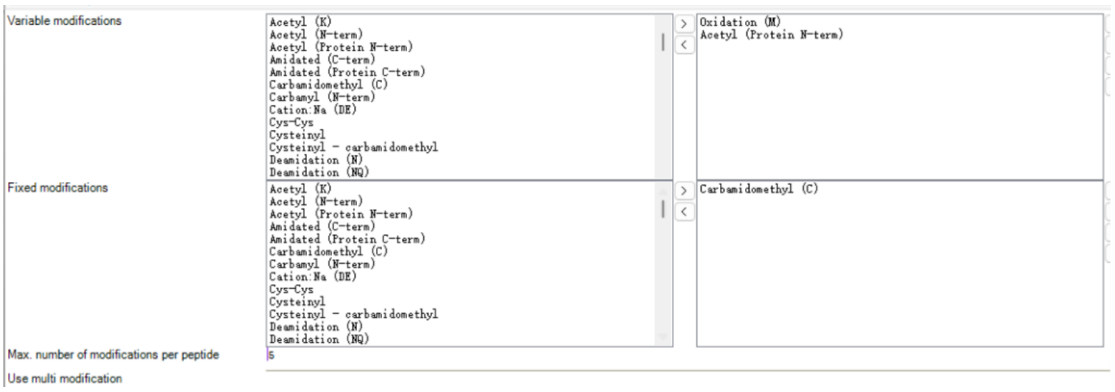

Label-free quantification: None.

Isobaric weight exponent 0.75

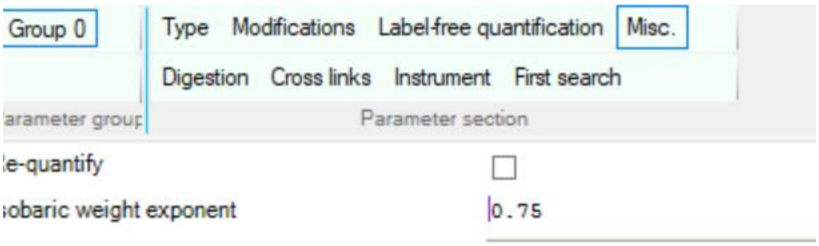

Digestion Digestion mode *Specific*; Enzyme *Trypsin/P*; Max. missed cleavages 2

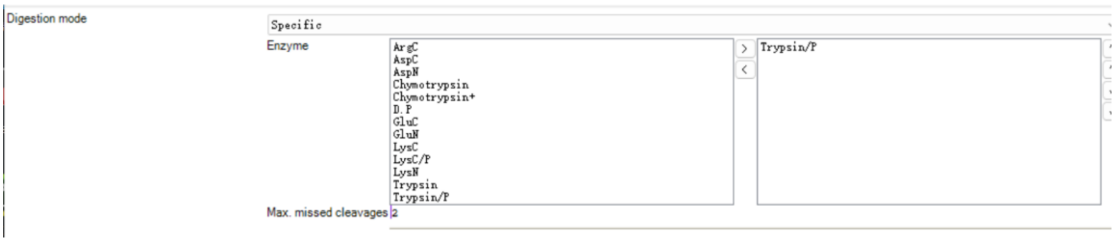

the instrument type is set as ***Orbitrap***; The remain settings are shown as following screenshots

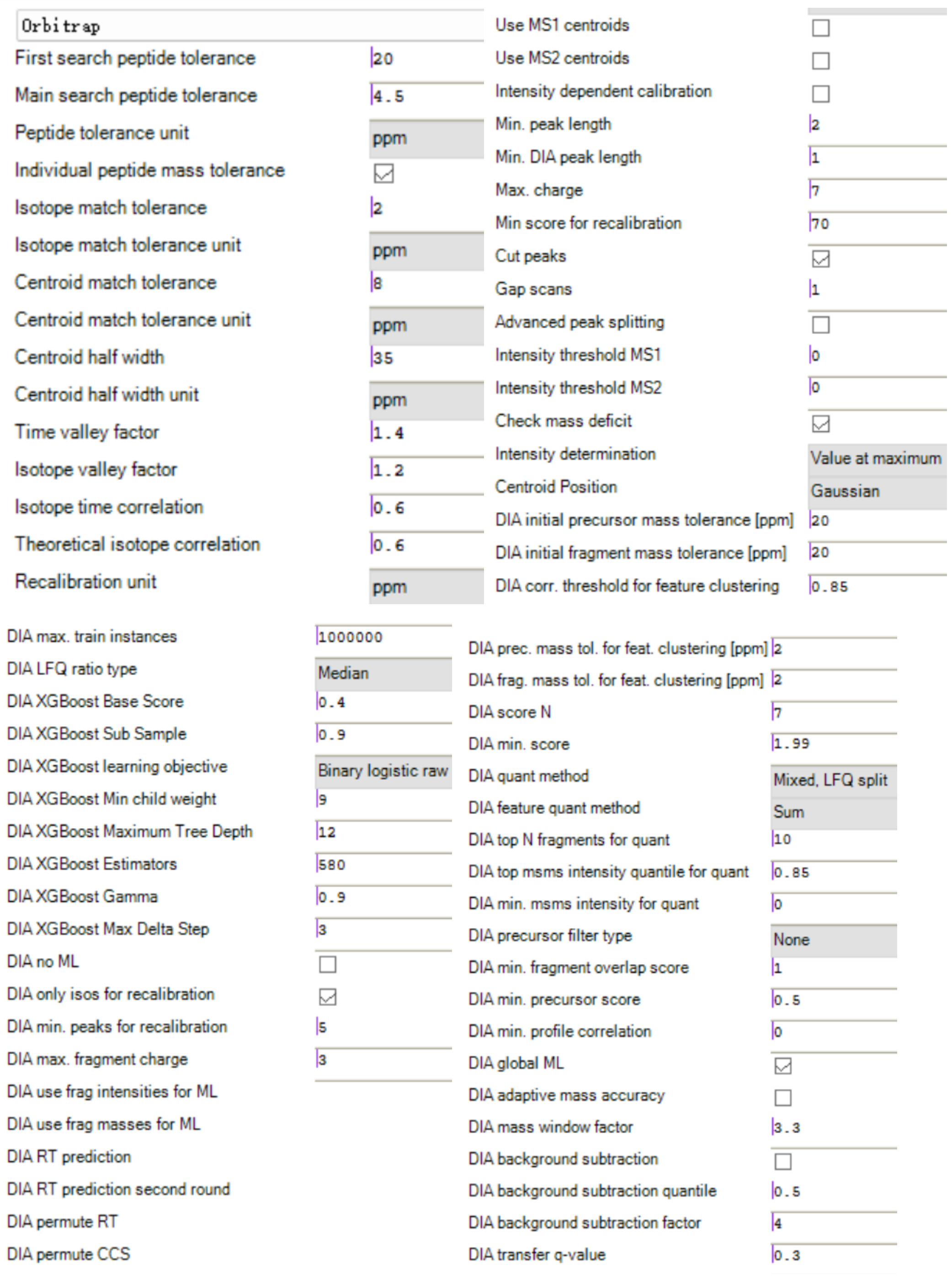

For database settings, we used the same databases downloaded from uniport as FragPipe and are shown in Supplementary Data 9. Other parameters include Include contaminants *Yes*; Min. peptide length *7*; Max. peptide mass [Da] *4600*; Min. peptide length for unspecific search *8*; Max peptide length for unspecific search *25*; Variation mode *None*

Include contaminants	☑
Min. peptide length	7
Max. peptide mass [Da]	4600
Min. peptide length for unspecific search	8
Max. peptide length for unspecific search	25
Protein grouping file	
Variation mode	None
Proteogenomics fasta files	

Protein quantification parameters: Label min. ratio count <u>2</u>; Peptides for quantification <u>*Unique+razor*</u>; Use only unmodified peptides and…<u>*Yes*</u> and <u>*Oxidation (M)*</u> <u>*Acetyl (Protein N-term)*</u>; Discard unmodified counterpart peptides <u>*Yes*</u>; Advanced ratio estimation <u>*Yes*</u>

Label min. ratio count	2		
Peptides for quantification	Unique + razor		
Use only unmodified peptides and...	☑		
	Modifications used in protein quantificatior	Acetyl (K)<br>Acetyl (N-term)<br>Acetyl (Protein N-term)<br>Amidated (C-term)<br>Amidated (Protein C-term)<br>Carbamidomethyl (C)<br>Carbamyl (N-term)<br>Cation:Na (DE)<br>Cys-Cys<br>Cysteinyl<br>Cysteinyl - carbamidomethyl<br>Deamidation (N)<br>Deamidation (NQ)	Oxidation (M)<br>Acetyl (Protein N-term)
	Discard unmodified counterpart peptides	☑	
Advanced ratio estimation	☑		
Custom protein quantification			

MS/MS analyzer parameters are shown as following screenshots:

FTMS MS/MS match tolerance	20
FTMS MS/MS match tolerance unit	ppm
FTMS MS/MS de novo tolerance	25
FTMS MS/MS de novo tolerance unit	ppm
FTMS MS/MS deisotoping tolerance	7
FTMS MS/MS deisotoping tolerance unit	ppm
FTMS top peaks per Da interval	12
FTMS top x mass window [Da]	100
FTMS de-isotoping	☑
FTMS higher charges	☑
FTMS water loss	☑
FTMS water loss for cross links	☐
FTMS ammonia loss	☑
FTMS ammonia loss for cross links	☐
FTMS dependent losses	☑
FTMS recalibration	☐

ITMS MS/MS match tolerance	0.5
ITMS MS/MS match tolerance unit	Da
ITMS MS/MS de novo tolerance	0.5
ITMS MS/MS de novo tolerance unit	Da
ITMS MS/MS deisotoping tolerance	0.15
ITMS MS/MS deisotoping tolerance unit	Da
ITMS top peaks per Da interval	8
ITMS top x mass window [Da]	100
ITMS de-isotoping	☐
ITMS higher charges	☑
ITMS water loss	☑
ITMS water loss for cross links	☐
ITMS ammonia loss	☑
ITMS ammonia loss for cross links	☐
ITMS dependent losses	☑
ITMS recalibration	☐

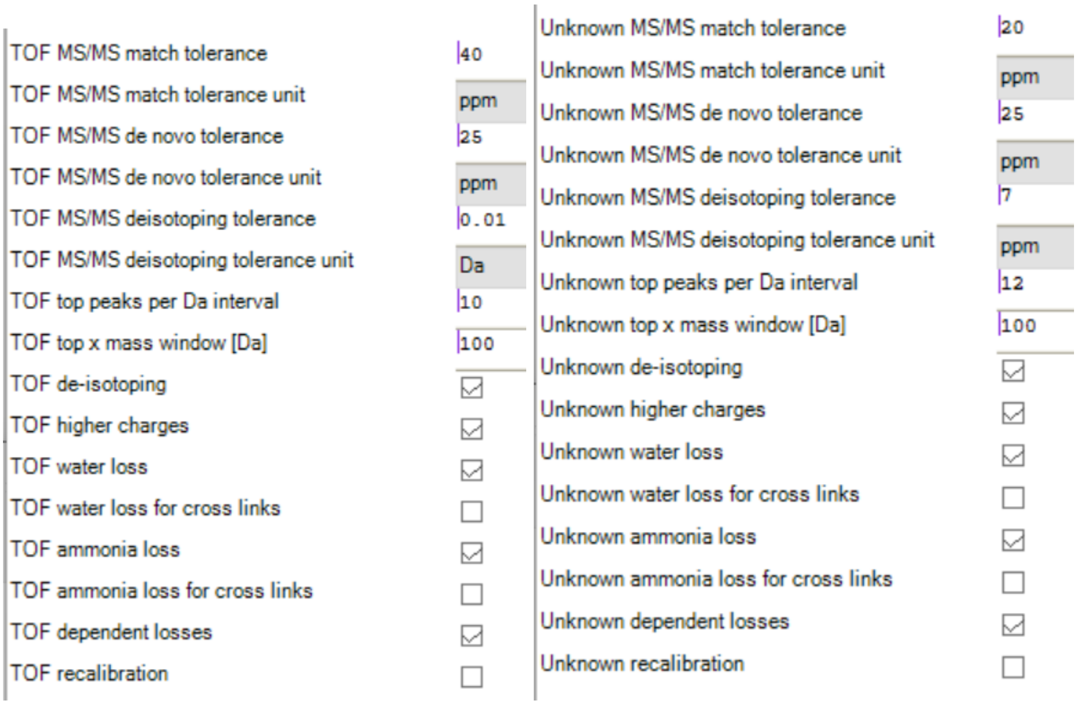

Advance setting parameters including Decoy mode *Revert*; Use for occupancies *Normalized ratios*; Epsilon score for mutations *Yes*; Evaluate proteogenomic peptides separately *Yes*;

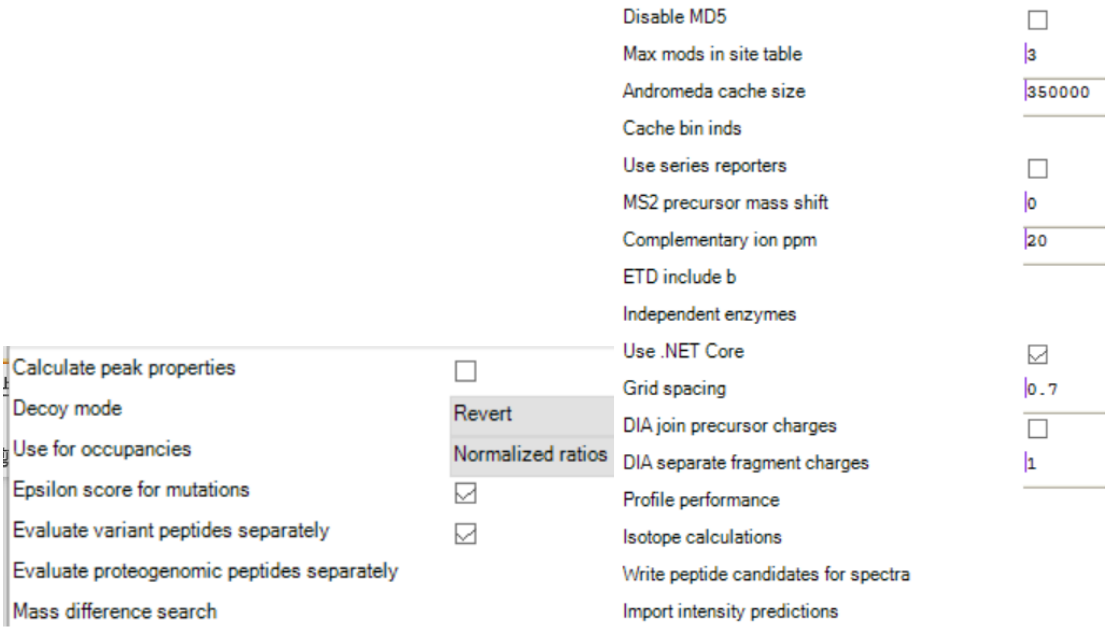

Identification parameters are shown in following screenshots and Match between runs is *disabled*:

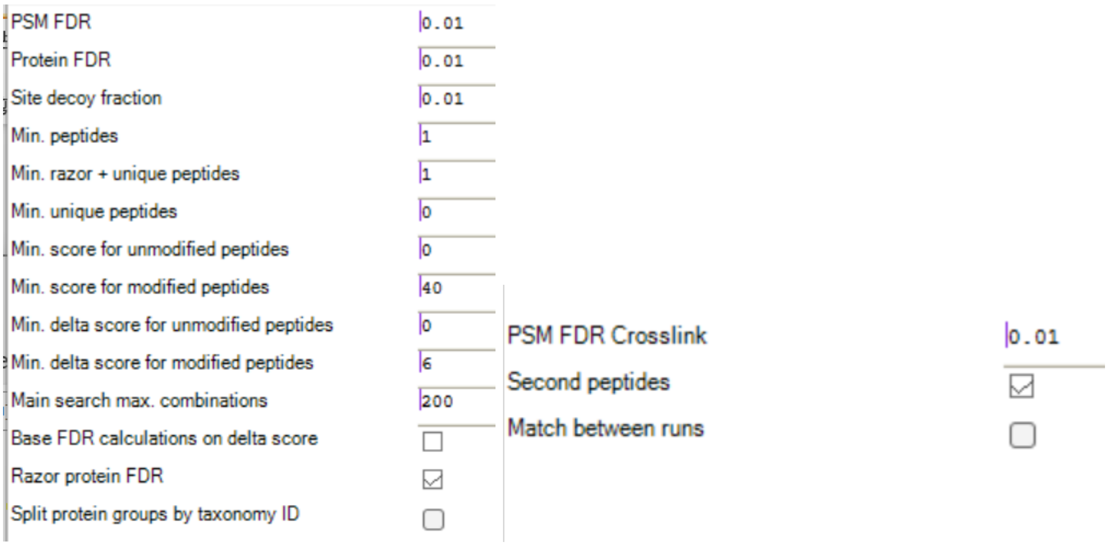

Supplementary Reference

1. Dowell, J. A., Wright, L. J., Armstrong, E. A. & Denu, J. M. Benchmarking Quantitative Performance in Label-Free Proteomics. *ACS Omega* **6**, 2494–2504 (2021).
2. Koopmans, F., Li, K. W., Klaassen, R. V. & Smit, A. B. MS-DAP Platform for Downstream Data Analysis of Label-Free Proteomics Uncovers Optimal Workflows in Benchmark Data Sets and Increased Sensitivity in Analysis of Alzheimer's Biomarker Data. *J. Proteome Res.* **22**, 374–386 (2023).
3. McClish, D. K. Analyzing a portion of the ROC curve. *Med. Decis. Mak. Int. J. Soc. Med. Decis. Mak.* **9**, 190–195 (1989).
4. Chicco, D. & Jurman, G. The advantages of the Matthews correlation coefficient (MCC) over F1 score and accuracy in binary classification evaluation. *BMC Genomics* **21**, 6 (2020).
5. Xuan, X., Lo, D., Xia, X. & Tian, Y. Evaluating defect prediction approaches using a massive set of metrics: an empirical study. in *Proceedings of the 30th Annual ACM Symposium on Applied Computing* 1644–1647 (Association for Computing Machinery, New York, NY, USA, 2015). doi:10.1145/2695664.2695959.
6. Kong, A. T., Leprevost, F. V., Avtonomov, D. M., Mellacheruvu, D. & Nesvizhskii, A. I. MSFragger: ultrafast and comprehensive peptide identification in mass spectrometry–based proteomics. *Nat. Methods* **14**, 513–520 (2017).
7. Prianichnikov, N. *et al.* MaxQuant Software for Ion Mobility Enhanced Shotgun Proteomics *. *Mol. Cell. Proteomics* **19**, 1058–1069 (2020).
8. Thompson, A. *et al.* Tandem Mass Tags: A Novel Quantification Strategy for Comparative Analysis of Complex Protein Mixtures by MS/MS. *Anal. Chem.* **75**, 1895–1904 (2003).
9. Demichev, V., Messner, C. B., Vernardis, S. I., Lilley, K. S. & Ralser, M. DIA-NN: neural networks and interference correction enable deep proteome coverage in high throughput. *Nat. Methods* **17**, 41–44 (2020).
10. Martinez-Val, A., Bekker-Jensen, D. B., Hogrebe, A. & Olsen, J. V. Data Processing and Analysis for DIA-Based PhosphoproteomicsPhosphoproteomics Using SpectronautSpectronaut. in *Proteomics Data Analysis* (ed. Cecconi, D.) 95–107 (Springer US,

New York, NY, 2021). doi:10.1007/978-1-0716-1641-3_6.
11. da Veiga Leprevost, F. *et al.* Philosopher: a versatile toolkit for shotgun proteomics data analysis. *Nat. Methods* **17**, 869–870 (2020).
12. Smyth, G. K. limma: Linear Models for Microarray Data. in *Bioinformatics and Computational Biology Solutions Using R and Bioconductor* (eds. Gentleman, R., Carey, V. J., Huber, W., Irizarry, R. A. & Dudoit, S.) 397–420 (Springer, New York, NY, 2005). doi:10.1007/0-387-29362-0_23.
13. Suomi, T., Seyednasrollah, F., Jaakkola, M. K., Faux, T. & Elo, L. L. ROTS: An R package for reproducibility-optimized statistical testing. *PLOS Comput. Biol.* **13**, e1005562 (2017).
14. Zhang, X. *et al.* Proteome-wide identification of ubiquitin interactions using UbIA-MS. *Nat. Protoc.* **13**, 530–550 (2018).
15. Zhu, Y. *et al.* DEqMS: A Method for Accurate Variance Estimation in Differential Protein Expression Analysis *. *Mol. Cell. Proteomics* **19**, 1047–1057 (2020).
16. Callister, S. J. *et al.* Normalization Approaches for Removing Systematic Biases Associated with Mass Spectrometry and Label-Free Proteomics. *J. Proteome Res.* **5**, 277–286 (2006).
17. Chawade, A., Alexandersson, E. & Levander, F. Normalyzer: a tool for rapid evaluation of normalization methods for omics data sets. *J. Proteome Res.* **13**, 3114–3120 (2014).
18. Stekhoven, D. J. & Bühlmann, P. MissForest—non-parametric missing value imputation for mixed-type data. *Bioinformatics* **28**, 112–118 (2012).
19. Kim, K.-Y., Kim, B.-J. & Yi, G.-S. Reuse of imputed data in microarray analysis increases imputation efficiency. *BMC Bioinformatics* **5**, 160 (2004).
20. Lazar, C., Gatto, L., Ferro, M., Bruley, C. & Burger, T. Accounting for the Multiple Natures of Missing Values in Label-Free Quantitative Proteomics Data Sets to Compare Imputation Strategies. *J. Proteome Res.* **15**, 1116–1125 (2016).
21. Kerr, M. K., Martin, M. & Churchill, G. A. Analysis of Variance for Gene Expression Microarray Data. *J. Comput. Biol.* **7**, 819–837 (2000).
22. Lix, L. M., Keselman, J. C. & Keselman, H. J. Consequences of Assumption Violations Revisited: A Quantitative Review of Alternatives to the One-Way Analysis of Variance F Test. *Rev. Educ. Res.* **66**, 579–619 (1996).
23. Van Puyvelde, B. *et al.* A comprehensive LFQ benchmark dataset on modern day acquisition strategies in proteomics. *Sci. Data* **9**, 126 (2022).
24. Ammar, C., Schessner, J. P., Willems, S., Michaelis, A. C. & Mann, M. Accurate Label-Free Quantification by directLFQ to Compare Unlimited Numbers of Proteomes. *Mol. Cell. Proteomics* **22**, 100581 (2023).
25. Cox, J. *et al.* Accurate proteome-wide label-free quantification by delayed normalization and maximal peptide ratio extraction, termed MaxLFQ. *Mol. Cell. Proteomics MCP* **13**, 2513–2526 (2014).
26. Goeminne, L. J. E., Sticker, A., Martens, L., Gevaert, K. & Clement, L. MSqRob Takes the Missing Hurdle: Uniting Intensity- and Count-Based Proteomics. *Anal. Chem.* **92**, 6278–6287 (2020).
27. O'Connell, J. D., Paulo, J. A., O'Brien, J. J. & Gygi, S. P. Proteome-Wide Evaluation of Two Common Protein Quantification Methods. *J. Proteome Res.* **17**, 1934–1942 (2018).
28. McKight, P. E. & Najab, J. Kruskal-Wallis Test. in *The Corsini Encyclopedia of Psychology*

1–1 (John Wiley & Sons, Ltd, 2010). doi:10.1002/9780470479216.corpsy0491.
29. Lundgren, D. H., Hwang, S.-I., Wu, L. & Han, D. K. Role of spectral counting in quantitative proteomics. *Expert Rev. Proteomics* **7**, 39–53 (2010).
30. Silva, J. C., Gorenstein, M. V., Li, G.-Z., Vissers, J. P. C. & Geromanos, S. J. Absolute quantification of proteins by LCMSE: a virtue of parallel MS acquisition. *Mol. Cell. Proteomics MCP* **5**, 144–156 (2006).
31. Gatto, L., Gibb, S. & Rainer, J. MSnbase, Efficient and Elegant R-Based Processing and Visualization of Raw Mass Spectrometry Data. *J. Proteome Res.* **20**, 1063–1069 (2021).
32. Wulff, J. E. & Mitchell, M. W. A Comparison of Various Normalization Methods for LC/MS Metabolomics Data. *Adv. Biosci. Biotechnol.* **09**, 339 (2018).
33. Bolstad, B. M., Irizarry, R. A., Åstrand, M. & Speed, T. P. A comparison of normalization methods for high density oligonucleotide array data based on variance and bias. *Bioinformatics* **19**, 185–193 (2003).
34. Brombacher, E., Schad, A. & Kreutz, C. Tail-Robust Quantile Normalization. *Proteomics* **20**, e2000068 (2020).
35. Huber, W., Heydebreck, A., Sültmann, H., Poustka, A. & Vingron, M. Variance Stabilization Applied to Microarray Data Calibration and to the Quantification of Differential Expression. *Bioinforma. Oxf. Engl.* **18 Suppl 1**, S96-104 (2002).
36. Willforss, J., Chawade, A. & Levander, F. NormalyzerDE: Online Tool for Improved Normalization of Omics Expression Data and High-Sensitivity Differential Expression Analysis. *J. Proteome Res.* **18**, 732–740 (2019).
37. Ritchie, M. E. *et al.* limma powers differential expression analyses for RNA-sequencing and microarray studies. *Nucleic Acids Res.* **43**, e47 (2015).
38. Välikangas, T., Suomi, T. & Elo, L. L. A systematic evaluation of normalization methods in quantitative label-free proteomics. *Brief. Bioinform.* **19**, 1–11 (2018).
39. Webb-Robertson, B.-J. M. *et al.* Review, evaluation, and discussion of the challenges of missing value imputation for mass spectrometry-based label-free global proteomics. *J. Proteome Res.* **14**, 1993–2001 (2015).
40. Jiang, Y. *et al.* Proteomics identifies new therapeutic targets of early-stage hepatocellular carcinoma. *Nature* **567**, 257–261 (2019).
41. Troyanskaya, O. *et al.* Missing value estimation methods for DNA microarrays. *Bioinforma. Oxf. Engl.* **17**, 520–525 (2001).
42. Shah, J. S. *et al.* Distribution based nearest neighbor imputation for truncated high dimensional data with applications to pre-clinical and clinical metabolomics studies. *BMC Bioinformatics* **18**, 114 (2017).
43. Buuren, S. van & Groothuis-Oudshoorn, K. mice: Multivariate Imputation by Chained Equations in R. *J. Stat. Softw.* **45**, 1–67 (2011).
44. Wei, R. *et al.* Missing Value Imputation Approach for Mass Spectrometry-based Metabolomics Data. *Sci. Rep.* **8**, 663 (2018).
45. Li, Q. *et al.* GMSimpute: a generalized two-step Lasso approach to impute missing values in label-free mass spectrum analysis. *Bioinforma. Oxf. Engl.* **36**, 257–263 (2020).
46. Oba, S. *et al.* A Bayesian missing value estimation method for gene expression profile data. *Bioinforma. Oxf. Engl.* **19**, 2088–2096 (2003).
47. Ibrahim, J. G., Chen, M.-H., Lipsitz, S. R. & Herring, A. H. Missing-Data Methods for

Generalized Linear Models. *J. Am. Stat. Assoc.* **100**, 332–346 (2005).
48. Verboven, S., Branden, K. V. & Goos, P. Sequential imputation for missing values. *Comput. Biol. Chem.* **31**, 320–327 (2007).
49. Branden, K. V. & Verboven, S. Robust data imputation. *Comput. Biol. Chem.* **33**, 7–13 (2009).
50. Choi, M. *et al.* MSstats: an R package for statistical analysis of quantitative mass spectrometry-based proteomic experiments. *Bioinforma. Oxf. Engl.* **30**, 2524–2526 (2014).
51. Ahlmann-Eltze, C. & Anders, S. proDA: Probabilistic Dropout Analysis for Identifying Differentially Abundant Proteins in Label-Free Mass Spectrometry. 661496 Preprint at https://doi.org/10.1101/661496 (2020).
52. Tusher, V. G., Tibshirani, R. & Chu, G. Significance analysis of microarrays applied to the ionizing radiation response. *Proc. Natl. Acad. Sci. U. S. A.* **98**, 5116–5121 (2001).
53. Robinson, M. D., McCarthy, D. J. & Smyth, G. K. edgeR: a Bioconductor package for differential expression analysis of digital gene expression data. *Bioinforma. Oxf. Engl.* **26**, 139–140 (2010).
54. Pavelka, N. *et al.* A power law global error model for the identification of differentially expressed genes in microarray data. *BMC Bioinformatics* **5**, 203 (2004).
55. Baggerly, K. A., Deng, L., Morris, J. S. & Aldaz, C. M. Differential expression in SAGE: accounting for normal between-library variation. *Bioinforma. Oxf. Engl.* **19**, 1477–1483 (2003).
56. Kalxdorf, M., Müller, T., Stegle, O. & Krijgsveld, J. IceR improves proteome coverage and data completeness in global and single-cell proteomics. *Nat. Commun.* **12**, 4787 (2021).
57. Paulovich, A. G. *et al.* Interlaboratory Study Characterizing a Yeast Performance Standard for Benchmarking LC-MS Platform Performance *. *Mol. Cell. Proteomics* **9**, 242–254 (2010).
58. Pursiheimo, A. *et al.* Optimization of Statistical Methods Impact on Quantitative Proteomics Data. *J. Proteome Res.* **14**, 4118–4126 (2015).
59. Ramus, C. *et al.* Spiked proteomic standard dataset for testing label-free quantitative software and statistical methods. *Data Brief* **6**, 286–294 (2016).
60. Lou, R. *et al.* Benchmarking commonly used software suites and analysis workflows for DIA proteomics and phosphoproteomics. *Nat. Commun.* **14**, 94 (2023).
61. Gotti, C. *et al.* Extensive and Accurate Benchmarking of DIA Acquisition Methods and Software Tools Using a Complex Proteomic Standard. *J. Proteome Res.* **20**, 4801–4814 (2021).
62. Navarrete-Perea, J., Gygi, S. P. & Paulo, J. A. HYpro16: A Two-Proteome Mixture to Assess Interference in Isobaric Tag-Based Sample Multiplexing Experiments. *J. Am. Soc. Mass Spectrom.* **32**, 247–254 (2021).
