# Supplementary material for: Optimizing differential expression analysis for proteomics data via high-performing rules and ensemble inference
Source: Nat Commun. 2024 May 9;15:3922. doi: 10.1038/s41467-024-47899-w (PMC11082229; doi:10.1038/s41467-024-47899-w)
Supplement: Supplementary file 2 — Reporting Summary [file 41467_2024_47899_MOESM2_ESM.pdf]

nature portfolio

Corresponding author(s): Wilson wen Bin Goh

Last updated by author(s): Apr 9, 2024

Reporting Summary

Nature Portfolio wishes to improve the reproducibility of the work that we publish. This form provides structure for consistency and transparency in reporting. For further information on Nature Portfolio policies, see our Editorial Policies and the Editorial Policy Checklist.

Statistics

For all statistical analyses, confirm that the following items are present in the figure legend, table legend, main text, or Methods section.

n/a	Confirmed	
☐	☒	The exact sample size (*n*) for each experimental group/condition, given as a discrete number and unit of measurement
☐	☒	A statement on whether measurements were taken from distinct samples or whether the same sample was measured repeatedly
☐	☒	The statistical test(s) used AND whether they are one- or two-sided<br>*Only common tests should be described solely by name; describe more complex techniques in the Methods section.*
☒	☐	A description of all covariates tested
☐	☒	A description of any assumptions or corrections, such as tests of normality and adjustment for multiple comparisons
☐	☒	A full description of the statistical parameters including central tendency (e.g. means) or other basic estimates (e.g. regression coefficient) AND variation (e.g. standard deviation) or associated estimates of uncertainty (e.g. confidence intervals)
☐	☒	For null hypothesis testing, the test statistic (e.g. *F*, *t*, *r*) with confidence intervals, effect sizes, degrees of freedom and *P* value noted<br>*Give P values as exact values whenever suitable.*
☒	☐	For Bayesian analysis, information on the choice of priors and Markov chain Monte Carlo settings
☒	☐	For hierarchical and complex designs, identification of the appropriate level for tests and full reporting of outcomes
☐	☒	Estimates of effect sizes (e.g. Cohen's *d*, Pearson's *r*), indicating how they were calculated

Our web collection on statistics for biologists contains articles on many of the points above.

Software and code

Policy information about availability of computer code

Data collection

FileZilla (3.46.3)

FragPipe (20.0)

MSFragger (3.8)

Philosopher (5.0.0)

MSBooster(1.1.11)

Percolator (3.06)

IonQuant (1.9.8)

MaxQuant (2.1.0.0)

MaxQuant (2.4.4.0)

DIA-NN (1.8.1)

Spectronaut 18

directlfq (0.2.11)

Data analysis	All codes is available at: https://github.com/PennHui2016/OpDENtree/mainkodes_DEA_benchmarking<br><br>Differential expression analysis:<br><br>OS: Windows 10-22H2<br><br>R 4.3.1<br><br>Rstudio 2023.06.2 Build 561<br><br>R packages:<br><br>NormalyzerDE-v1.18.1, limma-v3.56.2, ROTS-v1.28.0, MSnbase-v2.26.0, edgeR-v3.42.4, proDA-v1.14.0, DEqMS-v1.18.0, plgem-v1.72.0, DEP-v1.22.0, MSstats-v4.8.7, samr-v3.0, mice-v3.16.0, missForest-v1.5, SeqKnn-v1.0.1, reshape2-v1.4.4, dplyr-v1.1.3, tidyverse-v2.0.0, matrixStatsv1.0.0,<br>rrcovNA-v0.5.0, aggregation-v1.0.1, iq-v1.9.10 GMSimpute: see https://github.com/wangshisheng/NAguideR<br><br>workflow performance evaluation<br><br>OS: Windows 10-22H2<br><br>python_3.11.4<br><br>python packages:<br><br>pandas-v2.0.3 numpy-v1.24.3 sklearn-v1.3.0 scipy-v1.11.1 Anaconda3-2023.07-2-Windows-x86_64<br>machine learning and data mining for workflow analysis<br><br>OS: windows 10<br><br>python_3.11.4<br><br>python packages:<br><br>pandas-v2.0.3, numpy-v1.24.3, sklearn-v1.3.0, fpgrowth_py-v1.0.0, mlxtend-v0.23.0, catboost-v1.2.2<br><br>visualization<br><br>OS: Windows 11-23H2<br><br>R_4.3.1 2023.09.0 Build 463<br><br>R packages:<br><br>readxl-v1.4.3, ggplot2-v3.4.3, reshape2-v1.4.4, ggpubr-v0.6.0, cowplot-v1.1.1, openxlsx-v4.2.5.2, ggalluvial-v0.12.5, ggsci-v3.0.0, ggrepel ggthemes-v5.0.0,<br>ComplexHeatmap-v2.16.0, gridExtra-v2.3, UpSetR-v1.4.0, grid-v4.3.1, pROC-v1.18.4, ggradar-v0.2

For manuscripts utilizing custom algorithms or software that are central to the research but not yet described in published literature, software must be made available to editors and reviewers. We strongly encourage code deposition in a community repository (e.g. GitHub). See the Nature Portfolio guidelines for submitting code & software for further information.

Data

Policy information about availability of data

All manuscripts must include a data availability statement. This statement should provide the following information, where applicable:

- Accession codes, unique identifiers, or web links for publicly available datasets
- A description of any restrictions on data availability
- For clinical datasets or third party data, please ensure that the statement adheres to our policy

The raw proteomics data used in this work can be downloaded under the ProteomeXchange IDs or Proteomic Data Commons Study Identifiers listed in Table 1.
1. The datasets HYE5600735_DDA, HYE6600735_DDA, HYEqe735_DDA, HYEtims735_DDA and HYEtims735_DIA used in this study are available in the PRIDE database under accession code PXD028735 (https://proteomecentral.proteomexchange.org/cgi/GetDataset?ID=PXD028735).
2. The datasets HYtims134_DDA and HYtims134_DIA used in this study are available in the PRIDE database under accession code PXD036134 (https://

proteomecentral.proteomexchange.org/cgi/GetDataset?ID=PXD036134).
3. The dataset HEtims425_DDA used in this study is available in the PRIDE database under accession code PXD021425 (https://proteomecentral.proteomexchange.org/cgi/GetDataset?ID=PXD021425).
4. The dataset YUltq006_DDA used in this study is available in the Proteomic Data Commons database under accession code PDC000006 (https://proteomic.datacommons.cancer.gov/pdc/TechnologyAdvancementStudies/).
5. The dataset YUltq099_DDA used in this study is available in the PRIDE database under accession code PXD002099 (https://proteomecentral.proteomexchange.org/cgi/GetDataset?ID=PXD002099).
6. The dataset YUltq819_DDA used in this study is available in the PRIDE database under accession code PXD001819 (https://proteomecentral.proteomexchange.org/cgi/GetDataset?ID=PXD001819).
7. The datasets HEqe408_DDA and HEqe408_DIA used in this study is available in the PRIDE database under accession code PXD018408 (https://proteomecentral.proteomexchange.org/cgi/GetDataset?ID=PXD018408).
8. The datasets HYqfl683_DDA and HYqfl683_TMT11 used in this study is available in the PRIDE database under accession code PXD007683 (https://proteomecentral.proteomexchange.org/cgi/GetDataset?ID=PXD007683).
9. The dataset HYEtims777_DDA used in this study is available in the PRIDE database under accession code PXD014777 (https://proteomecentral.proteomexchange.org/cgi/GetDataset?ID=PXD014777).
10. The dataset MYtims709_DIA used in this study is available in the PRIDE database under accession code PXD034709 (https://proteomecentral.proteomexchange.org/cgi/GetDataset?ID=PXD034709).
11. The datasets HEof_n600_DIA and HEof_w600_DIA used in this study are available in the PRIDE database under accession code PXD026600 (https://proteomecentral.proteomexchange.org/cgi/GetDataset?ID=PXD026600).
12. The dataset HEqe777_DIA used in this study is available in the PRIDE database under accession code PXD019777 (https://proteomecentral.proteomexchange.org/cgi/GetDataset?ID=PXD019777).
13. The dataset HEqe277_TMT10 used in this study is available in the PRIDE database under accession code PXD013277 (https://proteomecentral.proteomexchange.org/cgi/GetDataset?ID=PXD013277).
14. The datasets HYms2faims815_TMT16, HYsps2815_TMT16, and HYms2815_TMT16 used in this study are available in the PRIDE database under accession code PXD020815 (https://proteomecentral.proteomexchange.org/cgi/GetDataset?ID=PXD020815).
More details of these datasets can be found in supp9. Tab1 in Supplementary Data 9.

All the quantification results, extracted expression matrices, and our benchmarking results are available at our website: http://www.ai4pro.tech:3838 or through Zenodo at https://doi.org/10.5281/zenodo.10482353 for raw quantification results, https://doi.org/10.5281/zenodo.10484253 for extracted expression matrices and https://doi.org/10.5281/zenodo.10484428 for benchmarking results.

Research involving human participants, their data, or biological material

Policy information about studies with human participants or human data. See also policy information about sex, gender (identity/presentation), and sexual orientation and race, ethnicity and racism.

Reporting on sex and gender	N/A
Reporting on race, ethnicity, or other socially relevant groupings	N/A
Population characteristics	N/A
Recruitment	N/A
Ethics oversight	N/A

Note that full information on the approval of the study protocol must also be provided in the manuscript.

Field-specific reporting

Please select the one below that is the best fit for your research. If you are not sure, read the appropriate sections before making your selection.

☒ Life sciences ☐ Behavioural & social sciences ☐ Ecological, evolutionary & environmental sciences

For a reference copy of the document with all sections, see nature.com/documents/nr-reporting-summary-flat.pdf

Life sciences study design

All studies must disclose on these points even when the disclosure is negative.

Sample size

1. The datasets HYE5600735_DDA, HYE6600735_DDA, HYEqe735_DDA, HYEtims735_DDA and HYEtims735_DIA contain 2 types of samples each with 9 replicates where sample A contains 5% of Ecoli weight for weight (w/w), 30% Yeast and 65% Human while sample B contains 20% Ecoli, 15% Yeast and 65% Human.
2. The datasets HYtims134_DDA and HYtims134_DIA contain three types of two-proteome spike-in samples created by mixing 50ng HeLa per sample with 12.5ng (conditionB), 15.625 (conditionC) or 18.75 Yeast (conditionD) respectively, each with 3 replicates.
3. The dataset HEtims425_DDA contains five groups of samples each with 3 replicates where the same HeLa samples were mixed with 0%, 3%, 4.5%, 6%, 7.5% or 9% (wt/wt) of E. coli sample.
4. In the dataset YUltq006_DDA, five concentrations of 0.25 (A), 0.74 (B), 2.2 (C), 6.7 (D) or 20 (E) fmol/μL of 48 UPS1 proteins were spiked in

yeast proteins and 3 replicates were generated for each of the five mixtures.
5. In the dataset YUltq099_DDA, five conditions of samples are available with 48 human UPS1 proteins with concentrations of 2 (A), 4 (B), 10 (C), 25 (D), and 50 (E) fmol/µL were added to trypsin-digested soluble yeast proteins, 3 replicates are available for each sample.
6. In the dataset YUltq819_DDA, 48 UPS1 proteins at concentrations 0.05 (A), 0.125 (B), 0.25 (C), 0.5 (D), 2.5 (E), 5 (F), 12.5 (G), 25 (H) or 50 (I) fmol/ml were mixed with yeast lysate, each sample has three replicates.
7. The datasets HEqe408_DDA and HEqe408_DIA were generated by spiking 100 µg (A) or 200 µg (B) of the E. coli lysate into 400 µg of the human lysate (HEK293 cell lysate) and then 8 replicates for each sample were generated.
8. In the datasets HYqfl683_DDA and HYqfl683_TMT11, Yeast lysate was spiked into human lysate to 10% of total protein concentration (1× group) (A, 3replicates), 5% (2× group) (B, 4 replicates), and 3.3% (3× group) (C, 4 replicates) for a total of 11 samples.
9. The dataset HYEtims777_DDA was generated by mixing tryptic protein digests of H. sapiens (HeLa), S. cerevisiae (Promega) and E.coli (Waters) in two different experiments leading to a ratio of 1:1 (HeLa), 1:2 (S. cerevisiae, Yeast) and 1:4 (E.coli, Ecoli) between the two samples.
10. In the dataset MYtims709_DIA, each replicate of mouse membrane protein digest was spiked into one replicate of yeast protein digest to generate one reference (containing 20% mouse membrane proteome) and six samples for comparison (containing 5% (A), 10% (B), 13% (D), 20% (C), 30% (E) and 40% (F) mouse membrane proteome), 5 replicates for each of the sample were generated.
11. The datasets HEof_n600_DIA and HEof_w600_DIA are generated by spiking the 48 human proteins of UPS1 (Sigma) into a whole cell extract of E.coli at 8 different concentrations ranging from 0.1 to 50 fmol of UPS1/ug of E.coli (A:0.1, B:0.25, C:1, D:2.5, E:5, F:10, G:25, H:50). Each sample has been trypsin-digested analyzed in triplicate.
12. In the dataset HEqe777_DIA, spike-in samples were prepared by mixing HeLa sample with 0%, 3%, 4.5%, 6%, 7.5% or 9% (wt/wt) of E. coli sample (n=3).
13. In the dataset HEqe277_TMT10 samples with different spiked in amounts of E. coli protein extract (3 replicates with 7.5 µg (A), 4 with 15 µg (B) and 3 with 45 ug (C)) in MCF-7 background (70 µg of protein extract) were prepared.
14. In the datasets HYms2faims815_TMT16, HYsps2815_TMT16, and HYms2815_TMT16, the HYpro16 standard consists of TMTpro-labeled human peptides at a 1:1 (A) ratio across all channels into were mixed with the TMTpro-labeled yeast peptides in triplicate at 20:1 (E), 10:1 (D), 4:1 (C), and 2:1 (B) ratios.

Data exclusions	No data were excluded from this study
Replication	This study is to benchmark proteomics data differential expression analysis workflows rather than biological finding. The quantification data, the benchmarking results and the codes used to conduct differential expression analysis and following benchmark result analysis are available to facilitate reproducibility of the computational analysis.
Randomization	For datasets with more than 3 contrasts available, we randomly chose contrasts for following workflow performance benchmarking for avoiding the performance being dominated by a single dataset. For each of the datasets YUltq006_DDA and YUltq099_DDA, 2 contrasts were chosen from 10 available contrasts. For dataset YUltq819_DDA, 3 contrasts were chosen from 36 available contrasts. For dataset MYtims709_DIA, 3 contrasts were chosen from 15 available contrasts. For each of the datasets HEof_n600_DIA and HEof_w600_DIA, 3 contrasts were chosen from the 28 available contrasts. For each of the datasets HYms2faims815_TMT16, HYsps2815_TMT16 and HYms2815_TMT16, 3 contrasts were chosen from 10 available contrasts.
Blinding	All the computational methods were blinded to the ground-truth. The output from the methods were compared to the groud-truth available

Reporting for specific materials, systems and methods

We require information from authors about some types of materials, experimental systems and methods used in many studies. Here, indicate whether each material, system or method listed is relevant to your study. If you are not sure if a list item applies to your research, read the appropriate section before selecting a response.

Materials & experimental systems

n/a	Involved in the study
☒	☐ Antibodies
☒	☐ Eukaryotic cell lines
☒	☐ Palaeontology and archaeology
☒	☐ Animals and other organisms
☒	☐ Clinical data
☒	☐ Dual use research of concern
☒	☐ Plants

Methods

n/a	Involved in the study
☒	☐ ChIP-seq
☒	☐ Flow cytometry
☒	☐ MRI-based neuroimaging

Plants

Seed stocks	N/A
Novel plant genotypes	N/A
Authentication	N/A
