# Supplementary material for: Optimizing differential expression analysis for proteomics data via high-performing rules and ensemble inference
Source: Nat Commun. 2024 May 9;15:3922. doi: 10.1038/s41467-024-47899-w (PMC11082229; doi:10.1038/s41467-024-47899-w)
Supplement: Supplementary file 15 — Description of Additional Supplementary Files [file 41467_2024_47899_MOESM15_ESM.pdf]

Description of Additional Supplementary Files

File Name: Supplementary Data 1

Description: The LODOCV results for measuring the generalizability of our benchmarking under different settings.

supp1. Tab1~supp1. Tab6 give the LODOCV results under settings of FG_DDA, MQ_DDA, DIANN_DIA, spt_DIA, FG_TMT and MQ_TMT respectively. In each table, the first column (dataset) shows the dataset name. The second column (contrast_index) is the contrast index for each dataset showing in the first column. The third column (mean_spearman) shows the LODOCV performances indicated by the spearman correlation coefficients, and a workflow's performance is indicated by the mean performance across all the testing datasets during the benchmarking. Similarly, the fourth column (median_spearman) presents the spearman correlation coefficients of the LODOCVs but the workflow's performance is calculated as the median performance across the testing datasets. The last two columns (mean_pearson and median_pearson) are the results of corresponding mean and median performance based LODOCVs indicated by the Pearson's correlation instead of the spearman correlation. Below the tables, we show the mean and median correlation coefficients.

File Name: Supplementary Data 2.

Description: The workflow lists, our benchmarking results and the Kruskal-Wallis test results.

supp2. Tab1-supp2. Tab6 give the workflow list, mean and median performance for each metric (e.g., mean_pauc001 refers to the average pAUC(0.01) score across the benchmark datasets), their ranking positions based on single metrics (e.g., rank_mean_pauc001 refers to the ranking of workflows based on their mean_pauc001 values), their average ranks across the five metrics (e.g., avg_rank_mean, calculated by averaging rank_mean_pauc001, rank_mean_pauc005, etc.), and their final rank positions (the last column in each table, obtained by ranking the workflows with their avg_rank_mean value). supp2. Tab7 shows the Kruskal-Wallis test results of the top 30 workflows under each of the settings.

File Name: Supplementary Data 3.

Description: Workflow performance levels and classification results with CatBoost.

In each sheet, three tables were used to show the workflow performance level classification results under a specific quantification setting. For example, in the sheet "FG_DDA", supp3. Tab1 shows the details of the workflows and their label and class information. We labelled the

workflows ranking at top 5% as the “H” class and have the label of 1. If the workflow is ranked between 5% and 25%, then it has the class of “RH” and label of 2. Similarly, the 25%-50% workflows are in “RL” class with labels of 3 and the remaining bottom 50% workflows are in “L” class and with the labels of 0. In supp3. Tab2, the performance level classification results of the 10-fold cross validation are shown. We used different performance indicators such as F1 score and MCC, the mean performances (mean) and their standard deviations (std) are shown in the last two rows. In supp3. Tab3, the feature importances are shown.

File Name: Supplementary Data 4.

Description: The results of using the linear model-based for checking the interactions between the workflow step options and the workflow ranking.

In each sheet, the results of the linear regression model for evaluating the interaction between predictive variables and the response values, and the extracted ANOVA table are shown. For example, in the sheet “FG_DDA”, the FG_DDA workflows related linear model results are shown in supp4. Tab1. In supp4. Tab2, the extracted ANOVA table is presented.

File Name: Supplementary Data 5.

Description: Frequent pattern mining results.

In each sheet, the frequent pattern mining results are shown for a specific setting. For example, in sheet “FG_DDA”, supp5. Tab1 gives the frequent patterns with support ration values higher than 0.1 and mined from “H” workflows under setting FG_DDA, while the supp5. Tab2 gives the frequent patterned with support ration values higher than 0.1 and mined from the “L” workflows of setting FG_DDA.

File Name: Supplementary Data 6.

Description: Detailed comparisons between options in each workflow step.

The sheets “FG_DDA”, “MQ_DDA”, “DIANN_DIA”, “spt_DIA”, “FG_TMT” and “MQ_TMT” store the pairwise comparison results of options in each workflow step. For example, in sheet “FG_DDA”, supp6. Tab1 lists the pairwise comparison of the available matrix types (intensities, the count matrix was excluded for comparision) from FragPipe’s outputs based on the workflows’ pAUC(0.01) scores. The supp6. Tab2-supp6. Tab5 compare the normalization methods, the imputation methods, the intensity-based DEA statistical tools and count-based DEA statistical tools respectively. Similarly, in remain tables of sheet “FG_DDA”, the comparison of options based on the other performance metrics, e.g., pAUC(0.05), pAUC(0.1), etc., are shown. In sheets “ranking_FG_DDA”,

“ranking_MQ_DDA”, “ranking_DIANN_DIA”, “ranking_spt_DIA”, “ranking_ FG_TMT”, and “ranking_MQ_TMT”, the ranking and cross-setting rankings of the options based on the pairwise comparison results are presented. For example, in sheet “ranking_FG_DDA”, the supp6. Tab126 lists the ranking of Matrix types of top0, top3, MaxLFQ (LFQ) and directLFQ (dlfq), including the single metric-based rankings, and the average ranking across the five metrics.

File Name: Supplementary Data 7.

Description: Ensemble inference results and the ranking of ensemble inference workflows.

In this file, we list the results of ranking the ens_multi-quant workflows and ens_topk workflows in sheets “rank_FG_DDA”, “rank_MQ_DDA”, “rank_DIANN_DIA”, “rank_spt_DIA”, “rank_FG_TMT” and “rank_MQ_TMT”. The performance metric values tested on different benchmark datasets are shown in the sheets with prefix of “metrics_” in their names.

File Name: Supplementary Data 8.

Description: Comparison results of ensemble inference and Top 1st single workflows.

In the sheet “summary_compared_top_workflows”, we list the best single workflows (TOP1) workflows, the best ens_multi-quant workflows and the best ens_topk workflows under each setting in supp8. Tab1-supp8. Tab6 to compare their DEA performances. In supp8. Tab7, we show the detail performance gains obtained by the best ens_multi-quant workflows and the best ens_topk workflows. In the sheets “FG_DDA”, “MQ_DDA”, “DIANN_DIA”, “spt_DIA”, “FG_TMT” and “MQ_TMT”, we give the detail metric values of the compared workflows under the six settings.

File Name: Supplementary Data 9.

Description: Detailed information of benchmarking datasets.

In supp9. Tab1 (in sheet “dataset”), the datasets used for workflow benchmarking are shown. In sheet “statistics” (supp9. Tab2-supp9. Tab4), we list quantification information for the benchmark datasets such as the protein numbers can be detected and the missing rates in the expression matrices from the datasets.

File Name: Supplementary Data 10.

Description: Cross-setting comparison results.

In this file, we present the cross-setting comparison results with four pairs of datasets. In sheet “TP_FP_numbers”, the tables show the identified numbers of true positive (TP), true negative (TN), false positive (FP) and false negative (FN). In sheets “cross_setting_DEA_HYEtims735”, “cross_setting_DEA_HYqfl683”, “cross_setting_DEA_HEqe408” and “cross_setting_DEA_HYtims134”, the detailed proteins, DEA results of different workflows are shown.

File Name: Supplementary Data 11.

Description: Cross-instrument comparison results.

Similar to above Supplementary Data 10, this file contains the results of the cross-instrument comparison. In sheet “TP_FP_numbers”, the supp11. Tab1 gives the numbers of identified TPs, FPs, TNs, FNs by different workflows. In sheet “cross_instrument_DEA_res”, we list the details of the DEA results obtained by the compared workflows.

File Name: Supplementary Information

Description: supplementary figures, supplementary tables and supplementary notes.

The supplementary figures and supplementary tables show the results having not been presented in the main text. The supplementary notes include Supplementary Note 1 for described our findings having not been discussed in our main text and Supplementary Note 2 for showing details of the methods we used but having not been described in the Methods part of our main text.
